# Structural and functional insights into a Zn^2+^-transporting P-type ATPase

**DOI:** 10.1038/s41467-026-77558-1

**Published:** 2026-09-16

**Authors:** Pin Lyu, Elena Longhin, Fatemeh Sabzian-Molaei, Fernando Montalvillo Ortega, Ping Li, Kaituo Wang, Annette Duelli, Tamim Al-Jubair, Hajira Ahmed Hotiana, Eva Ramos Becares, Tristan Croll, Gabriele Meloni, Magnus Andersson, Viktoria Bågenholm, Pontus Gourdon

**Affiliations:** 1 Department of Biomedical Sciences, University of Copenhagen, Copenhagen, Denmark; 2 Department of Chemistry, Umeå University, Umeå, Sweden; 3 Department of Chemistry and Biochemistry, University of Texas at Dallas, Richardson, TX USA; 4 Department of Experimental Medical Science, Lund University, Lund, Sweden; 5 Department of Haematology, University of Cambridge, Cambridge, UK

**Keywords:** Ion channels, X-ray crystallography, Protein transport, Permeation and transport

## Abstract

Zinc is essential for life yet toxic in excess, rendering homeostatic control vital for all organisms. Transition metal transporting P-type (P_IB_-) ATPase proteins are critical for this balance but remain poorly understood at the molecular level. Here, we present the inward-open structure of a dimeric zinc-transporting P_IB_-type ATPase, resolved at 3.6 Å resolution. Biochemical analyses and molecular dynamics simulations suggest that the N-terminal tail, harboring two so-called metal binding domains (MBDs), auto-inhibits the ATPase core and serves as a metal-sensitive sensor, releasing inhibition when intracellular metal levels rise. Contrary to the prevailing uptake models, the structure reveals an electronegatively charged uptake region that directly exposes a metal-binding cysteine of the invariant CPC-motif. We propose that a histidine-rich segment, positioned after the MBD closest to the core, donates ions to the CPC site. Consequently, an intricate interplay between the regulatory and ion-providing N-terminus and the ATPase core is present, safeguarding against zinc toxicity. Collectively, our findings shed light on the regulation and transport mechanism of P_IB_-ATPases, with implications for fundamental biology and the potential to inform future biotechnological and translational applications.

## Abstract (web summary)

Zinc is essential for cellular metal homeostasis, but the molecular mechanisms of zinc-transporters remain poorly understood. Here, the authors reveal the structure and regulatory mechanism of a zinc-transporting P-type ATPase, providing insights into zinc uptake and autoregulation.

## Introduction

Zinc represents an essential micronutrient, present in numerous proteins as a cofactor that participates in chemical catalysis or protein stabilization^1–3^. Nevertheless, excessive zinc is detrimental, and thus cells have evolved transport systems to maintain homeostasis^4–11^. Transition metal-transporting P-type (P_IB_-) ATPases are critical in this process, mediating transfer of metals across cellular membranes—from the cytoplasm to the extracellular space or into organelles^12^. These proteins handle not only essential metal ions such as zinc and copper but also toxic substances like lead and cadmium^12^. Based on cargo specificity that correlates with cargo-binding sequence motifs in the transmembrane domain, P_IB_-members are divided into groups (e.g., P_IB-1_-P_IB-7_)^13,14^. P_IB-1_-type ATPases, which facilitate the removal of Cu^+^ and related ions such as Ag^+^, are perhaps the best characterized^15^. The significance of P_IB_-ATPases is underscored by the fact that defects in the human P_IB-1_-proteins ATP7A and ATP7B are the direct cause of the severe Menkes’ and Wilson’s diseases, respectively^16^. Other P_IB-_subgroups confer resistance to different divalent transition metals, which primarily have been identified in bacteria and plants^17–19^. Of these, the P_IB-2_-type ATPases (or ZntA) mediate the pumping of Zn^2+^, Pb^2+^, and Cd^2+^, typified by the relatively well-studied SsZntA from *Shigella sonnei*^20^, while the P_IB-4_-type ATPases may have a broader ion transport range, as exemplified by sCoaT from *Sulfitobacter* sp.* NAS14-1*^21^.

**Fig. 1:**
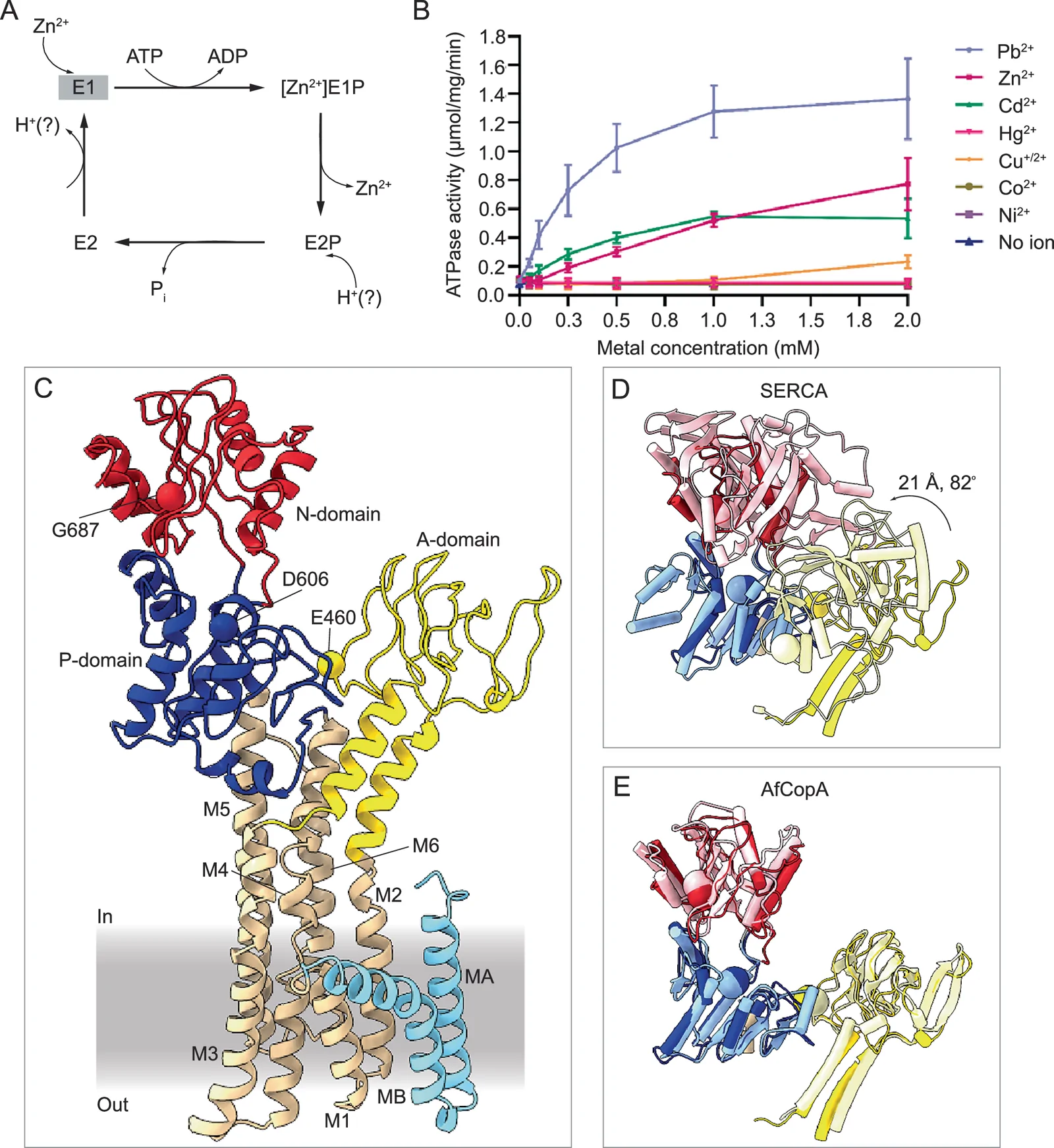
Overall structure and mechanism of MmCadA. **A** Schematic of P_IB-2_-ATPase (CadA) transport mechanism with the structurally determined state highlighted. **B** Wild-type MmCadA activity in lipid-detergent solution: The specific activity with Pb^2+^, Zn^2+^, or Cd^2+^ was 1.62 ± 0.21, 0.79 ± 0.05, and 0.55 ± 0.08 μmol mg^−1^ min^−1^, respectively; negligible activity was observed with other metals. Data are shown as mean ± SD. Error bars represent the standard deviation of three independent technical measurements performed using material from a single protein purification. **C** MmCadA adopts the classical P_IB_-type ATPase fold with soluble A- (actuator, yellow), P- (phosphorylation, red) and N- (nucleotide binding, blue) domains, as well as a transmembrane (M-) domain comprising helices MA and MB (cyan) and M1–M6 (wheat). **D** and **E** Structural comparison of MmCadA (colored) with SERCA in the [Ca_2_]E1-ATP state (PDB-ID: 1T5S, gray) and AfCopA in the E1 state (PDB-ID: 7R0I, gray). Catalytically critical residues (MmCadA numbering) are shown as spheres: P-domain D606, A-domain E460, N-domain G687. Relative to SERCA, the A-domain of MmCadA shifts away from the P-domain, but it aligns with the position of the A-domain in AfCopA.

P_IB_-ATPases belong to the P-type ATPase superfamily of proteins, which couple ATP hydrolysis to ion transport through the so-called Post-Albers cycle (Fig. 1A). This mechanism includes four consecutive cornerstone states^22^: E1, E1P, E2P, and E2, with P denoting ATP-induced phosphorylated conformations. E1 and E2 represent intermediates with high and low binding affinity for the exported cargo, allowing uptake and release, respectively. Transport is achieved by an overall conserved core of three intracellular A- (actuator), P- (phosphorylation) and N- (nucleotide binding) domains together with a transmembrane (M-) domain with a variable number of transmembrane spanning segments of which six (M1–M6) are maintained in all P-type ATPases^20,23^ (Supplementary Fig. 1). Adaptation of the topology permits transport of a range of different cargo. As an example, the M-domain of P_IB_-proteins carries two additional transmembrane helices in the N-terminus, MA and MB, as well as an amphiphilic helix at the membrane interface, MB’, that connects to M1. These structural features have been proposed to participate in ion uptake^20,21,23^. In contrast, P_IB_-ATPases lack the last four transmembrane segments present in the more classical P_II_-type ATPases, including SERCA (Sarco/endoplasmic reticulum Ca^2+^-ATPase, a P_2A_-ATPase) and the sodium pump (also known as the Na^+^/K^+^-ATPase, a P_2C_-ATPase)^24,25^.

Another class-specific feature of P_IB_-ATPases is the so-called metal binding domains (MBDs). These domains have been suggested to function as regulatory elements of the overall metal ion transport rate^26^, as metal-binding modules^27^, and/or as intermediates in metal delivery to the core^28^. The number of MBDs ranges from none to two in prokaryotes and up to six in eukaryotes, and they can occur at one or both termini flanking the core^16,17,26^. They typically adopt a βαββαβ ferredoxin-like fold with a solvent-exposed CXXC metal-binding motif, although alternative setups also exist^29^. The presence of an additional aspartate residue immediately upstream of the CXXC motif has been suggested to modulate metal selectivity in many ZntA-like ATPases^30,31^. In fact, it has even been proposed that the metal selectivity of P_IB_-ATPases can be changed by replacement of the MBD-containing N-terminus preceding the ATPase, or even by swapping the amino acids that surround the CXXC motifs^32,33^. In contrast, substitution of the N-terminus of ZntA with some or all six MBDs from ATP7B did not alter metal selectivity^34^. In P_IB-1_-ATPases, the MBDs seem to serve similar roles. The MBD closest to the core appears essential for transport, likely with a structural role, whereas more distal units serve autoinhibitory and metal-sensing roles^35^.

The termini of P_IB_-ATPases are frequently also rich in complementary metal-binding residues. For instance, the N-terminus of the P_IB-1_-ATPase LpCopA from *Legionella pneumophila* contains multiple histidine residues^23^ and the P_IB-2_- ATPase AtHMA4 harbors histidines and cysteines in the C-terminus that mediate Zn^2+^/Cd^2+^ binding and regulation^36^. In P_IB-3_- and P_IB-4_-type ATPases, histidines or combinations of histidines, cysteines, and aspartic acids are present before the MA transmembrane segment, while MBDs are rare^17,37^. As there are indications that MBDs serve similar roles based on their position from the core^35^, we will utilize a positional naming system: the MBD closest to the core is MBD^−1^, the one preceding that is MBD^−2^, and so on. This also applies to other features, such as stretches rich in metal-binding residues. Similarly, in the case of C-terminal features, they would be named MBD^1^, MBD^2^, and so on from the core.

Structural information on P_IB_-ATPases with an intact core is limited. Available high-resolution structures correspond to the E2P conformation and a transition state of dephosphorylation, E2.P_i_, of LpCopA^23^, SsZntA^20^, sCoaT^21^, and of copper-transporting ATP7B from frog^38^ and OsHMA4 (ref. ^35^) from rice. A few inward-open E1 structures exist, but only of P_IB-1_-ATPases transporting monovalent copper ions (AfCopA^39^, human ATP7B^40^, and OsHMA4 (ref. ^35^)), and no structure is available of an E1P conformation. Consequently, it remains poorly understood how regulation, ion uptake, and ion specificity and transport are accomplished in non-P_IB-1_-ATPases such as P_IB-2_-ATPases. This is relevant, considering the notable differences observed between P_IB-1_- and P_IB-2_-ATPases for metal release^20,23,41^. While these structures have been groundbreaking, one major limitation is that the termini have not been visible in most cases. The exceptions are the frog ATP7B structure, where the C-terminus, as well as MBD^−1^ and MBD^−2^ in the N-terminus were detected^38^, human ATP7B, with MBD^−1^ and MBD^−2^ (ref. ^40^), and OsHMA4, with MBD^−1^ (ref. ^35^). Thus, the functional role of the MBDs and other metal binding residues in the termini remain enigmatic, and it remains unknown if the N-termini of P_IB-2_-ATPases participate in providing metal selectivity, partake in metal transfer to the core, influence trafficking in eukaryotes and/or regulate metal transport rates.

Here we investigate MmCadA, a P_IB-2_-ATPase from *Mesorhizobium metallidurans*. We present the E1 structure of a P_IB_-ATPase involved in divalent ion transport and dissect the ion uptake principles. Moreover, the function of the N-terminus of MmCadA is studied using biochemical experiments, hinting at distinct roles of different parts of the N-terminus. This combined structural and functional analysis provides a framework to understand regulation and ion loading in P_IB_-ATPases.

## Results

### Metal specificity

To shed further light on the function of P_IB-2_-type ATPases, we first attempted to stabilize E1 states in SsZntA, but no crystals were obtained. We then screened five candidates with a varying number of MBDs and histidine-rich stretches in the N-terminus, MmCadA, TtCadA, TmCadA, PlCadA and MmZntA, assessing protein production yields by western blot (Supplementary Figs. 2 and 3). Out of these, MmCadA, derived from *Mesorhizobium metallidurans*, with two MBDs and two linking histidine-rich stretches displayed attractive protein levels (~8–10 mg/L) from *E. coli* and demonstrated favorable purity and stability following affinity and size-exclusion chromatography (Supplementary Fig. 4a). To investigate the transport cargo range of the purified protein, we exploited an established in vitro solution assay, detecting metal-stimulated release of inorganic phosphate in the presence of different transition metal ions (Fig. 1B, using three independent technical measurements from a single protein purification, as is the case for all subsequent functional data unless specified otherwise)^42^. This analysis reveals that MmCadA is stimulated by Pb^2+^, Zn^2+^, and Cd^2+^, but not Cu^+/2+^, Hg^2+^, Co^2+^, and Ni^2+^. As such, the findings are consistent with the related P_IB-2_-ATPases SsZntA and EcZntA, which previously have been shown to have a similar cargo specificity profile, although a broader transport range has also been reported for EcZntA^20,43,44^. The *V*_max_ of MmCadA is 1.62 ± 0.21, 0.79 ± 0.05, and 0.55 ± 0.08 µmol mg^−1^ min^−1^ in the presence of Pb^2+^, Zn^2+^, and Cd^2+^, respectively, similar to that previously shown for the equivalent activities of SsZntA^20^ and EcZntA^45^. Thus, MmCadA appears to have a similar cargo preference in vitro (Pb^2+^ > Zn^2+^ > Cd^2+^) as other more well-characterized P_IB-2_-type ATPases. Interestingly, at low metal concentrations, the Cd^2+^-stimulated activity is higher than Zn^2+^, while the pattern is the opposite at elevated levels (Fig. 1B). Since Zn^2+^ is an essential metal for cellular proteins, it is possible that evolution has favored internal sequestration at lower concentrations to ensure supply to metal-demanding macromolecules, while immediate export of Pb^2+^ and Cd^2+^ may be preferred due to their toxicity. Conversely, the detected inter-metal concentration dependence of transport rates is rather minor, and it may also reflect subtle differences in transport and coordination chemistry. Consistent with these results, a previous study suggested MmCadA primarily confers tolerance to Zn^2+^ at high concentrations in vivo^46^. Further validating the observed cargo promiscuity, a similar pattern was also observed in vivo with *E. coli* cells challenged with increasing Zn^2+^, Cd^2+^, and Pb^2+^ concentrations, where the presence of MmCadA, but not its inactive D606N mutant, protects against metal-induced toxicity when compared to cells transformed with a control vector (Supplementary Fig. 5a–l). These cells also show MmCadA-dependent resistance to intracellular Zn^2+^ accumulation (Supplementary Fig. 5m). Taken together, all the results are in agreement with MmCadA serving as a promiscuous Zn^2+^/Cd^2+^/Pb^2+^ exporter.

### Structure determination and overall structure

Next, we sought to determine the inward-facing structure of a P_IB-2_-ATPase. Considering other P_IB-_ATPases have recently been resolved using cryo-EM^35,38,40^, we initially applied this method, but these efforts were fruitless. Instead, we attempted crystallization of MmCadA in the presence of metal and phosphate or nucleotide mimics to stabilize an E1 conformation. Diffracting crystals were obtained in the presence of 2.5 mM Pb(CH_3_COO)_2_, 3 mM MgCl_2_, and 1 mM AMPPNP, in high amounts of lipids and detergent, bringing to mind conditions previously employed to recover a transition state of phosphorylation ([Ca_2_]E1-ATP) for the well-characterized P_2A_-ATPase SERCA (Supplementary Fig. 4b)^47,48^. While attempts to recover initial phases were unsuccessful using previously determined, homologous P_IB_-type ATPase structures, the structure was determined using molecular replacement with a search model obtained using AlphaFold^49^, and the final structure was refined to 3.6 Å resolution with *R*_work_/*R*_free_ of 27.7/31.5 (Fig. 1C, Supplementary Table 1, Supplementary Figs. 6–8). Overall, the architecture of MmCadA is preserved compared to previously characterized P_IB_-ATPases^20,23^, with the three soluble A-, P- and N-domains, as well as a membrane spanning M-domain formed by eight helices, MA, MB and M1–M6 (Fig. 1B). The P- and M-domains are well-defined in the final electron density map, while the other two cytoplasmic domains are poorly resolved, likely due to inherent flexibility (Supplementary Fig. 7). The generated model includes residues P291 to the carboxy-terminal L908, thus lacking the two predicted MBDs and histidine-rich regions, features that have also not been detected in most previously determined P_IB_-ATPase structures (Fig. 1C and Supplementary Fig. 7)^20,23,35,40^.

Unexpectedly, a tight dimer arrangement between two MmCadA monomers (chains A and B, respectively) is observed in the crystal packing, with interactions between the transmembrane and the P-domains of the separate monomers (Supplementary Figs. 8 and 9). Indeed, native-PAGE of the purified MmCadA does show a band indicative of a dimer, as well as a band corresponding to a tetramer, and the SEC profile of purified protein is also suggestive of a dimer (Supplementary Figs. 4 and 8). This is of potential interest as certain P-type ATPases, such as the sodium pump, have been proposed to be stabilized as a higher oligomeric assembly^50–52^, and recently the cryo-EM structure of the magnesium transporting P-type ATPase MgtA demonstrated a dimeric arrangement^53–55^. Moreover, the P_IB-1_-ATPase AfCopA has been shown to form monomer-monomer interactions in a low-resolution cryo-EM structure determined from tubular crystals^56^. However, the interfaces between the two MmCadA monomers are not conserved, seemingly precluding an important functional relevance of the observed dimer form (Supplementary Fig. 10). Supporting this notion, we note the rather intimate monomer-monomer setup in the crystal packing seemingly disfavors the coupling between the P- and M-domains, which is necessary for ATP turnover and ion transport. However, we cannot exclude that the assembly serves a purpose for stabilization or for allosteric regulation. Notably, the two monomers are similar but not identical (RMSD 0.9 Å) due to subtle shifts of the A- and N-domains, features that are closer to the P-domain in chain B (Supplementary Fig. 8). We speculate these differences refer to conformational flexibility of the captured intermediate and the effects of crystal packing, similar to what has been observed for other P-type ATPases^20,23^. The relatively weak electron density of the N-domain further supports this interpretation.

### An inward-open E1 conformation similar to P_IB-1_-ATPases

To incorporate the recovered MmCadA structure in the transport cycle, we first assessed classical hallmark features of the transport mechanism of P-type ATPases. Investigation of the surface accessibility of conserved metal-binding residues of transmembrane helices M4 and M6 suggests the MmCadA structure is inward-open, exposing several of these amino acids to the intracellular environment (see details below). No indication of bound or loosely attached metal is detected in the M-domain in the final maps, as is characteristic of early E1 states of SERCA^24,57^ (Supplementary Fig. 7). Presence of the nucleotide mimic AMPPNP was also not observed, although it cannot be excluded that this relates to the relatively low resolution of the N-domain.

We next compared the MmCadA structure with determined conformations of P_IB_-type ATPases. Inspection of structural alignments, based on both soluble domains and full-length models, and the corresponding RMSD values, suggests the structure is highly similar to that of AfCopA in an inward-open E1 conformation (PDB-ID 7R0I)^39^ (Fig. 1D, E and Supplementary Table 2). Both MmCadA monomers closely resemble this reference, with only minor chain-to-chain differences (Supplementary Fig. 8). In contrast, previously available P_IB_-ATPase structures representing the E2P and E2.P_i_ intermediates, including those of the P_IB-2_-ATPase SsZntA, are clearly distinct. Altogether, these observations support our assignment of the MmCadA structure to an early inward-open, E1-like state, primed for metal-ion uptake (Fig. 1C).

**Fig. 2:**
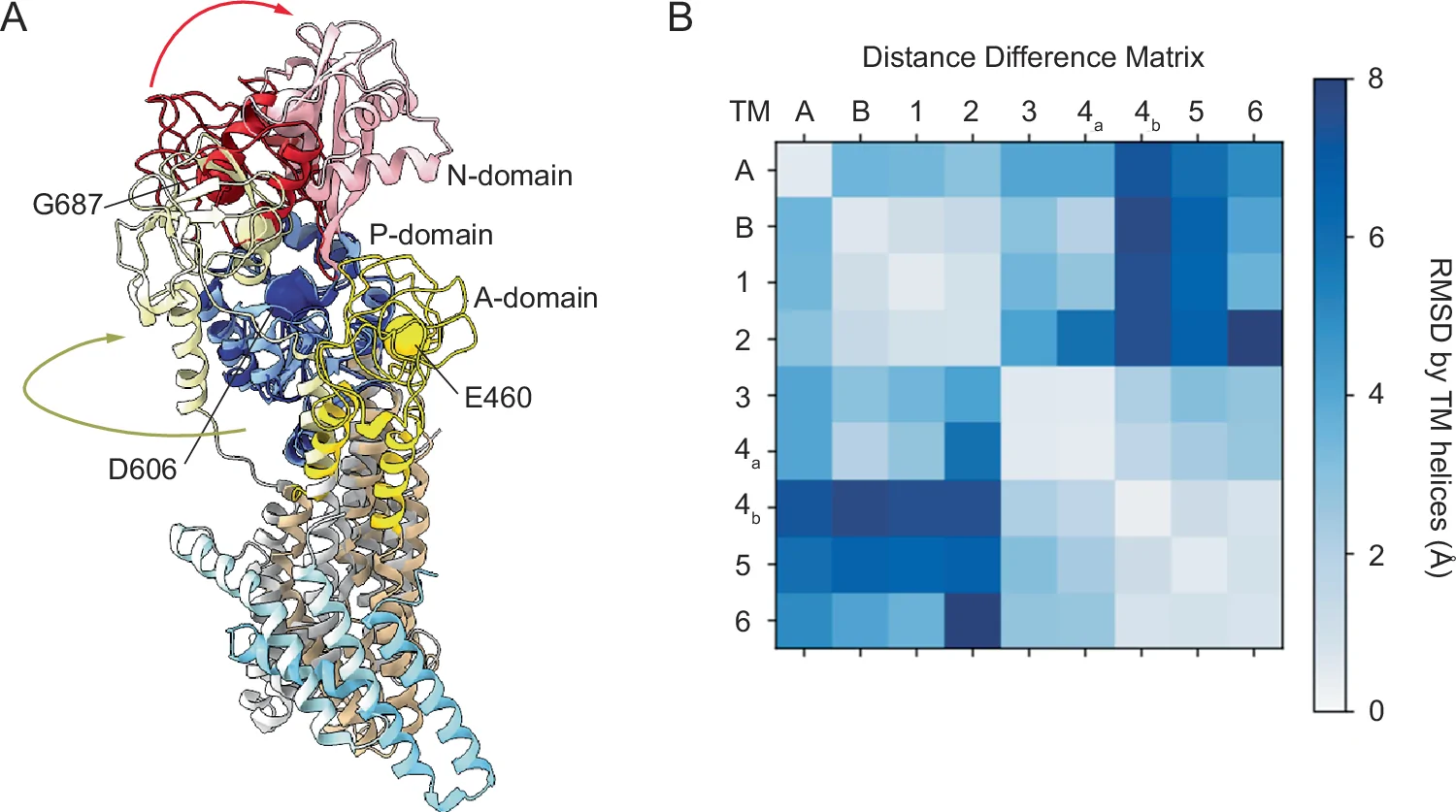
Conformational changes from E1 to E2P state. **A** Structural comparison of the E1 state of MmCadA (dark colors) with the previously characterized E2P state of SsZntA (light colors), aligned on the P-domain. Catalytically critical residues (MmCadA numbering) are shown as spheres: P-domain D606, A-domain E460, N-domain G687. The approximate location of the membrane is indicated. The A-domain undergoes a large rotation with the E1 to E2P transition. **B** Distance difference matrix of the M-domain (full-protein alignment) comparing MmCadA with an E2P SWISS-MODEL homology model based on SsZntA. Small intragroup distance changes were recorded for MA-M2 (upper left quadrant) and M3–M6 (bottom right quadrant) TM helices groups, but high intergroup distance rearrangements were observed between the aforementioned groups (bottom left and upper right quadrants as they are mirror images). The greatest displacements (~8 Å) are M4b and M6 relative to MB and M2, respectively^81–83^.

To analyze overall structural transitions in P_IB-2_-ATPases, we then compared our E1 structure with the metal-release E2P state of SsZntA (Supplementary Table 2)^20^. As expected, we detect considerable interdomain rearrangements that accompany the E1 to E2P shift: the N-domain separates from the P-domain (~15 Å, ~60°), leaving space for repositioning of the A-domain towards the P-domain to enable dephosphorylation (~30 Å, ~22°) (Fig. 2A). These changes result in a reorganization of the transmembrane domain that permits metal transport. Unbiased analysis of TM helix displacements between these two states suggests that MA-M2 and M3–M6 move as two independent bundles, similarly to what has been observed for P_IB-1_-ATPases^35,39^ (Fig. 2B). This segregation can be explained by the missing A-domain/M1 linker in P_IB_-ATPases (compared to P_II_-ATPases), leaving MA-M2 movements dependent mainly on the M2/A-domain connection (Supplementary Fig. 1). In contrast, SERCA exhibits a more intricate helix rearrangement pattern without such sub-domain partitioning. Notably, metal release is, however, not reliant on these hypothetical bundles in P_IB-2_-ATPases, as this occurs via a cleft in-between M4, M5, and M6^20^, thereby contrasting to the situation in P_IB-1_-ATPases^58^. Thus, the two-bundle model may be oversimplified, or, at least, the second subdomain undergoes internal conformational changes.

Subsequently, we contrasted the MmCadA configuration with determined structures of SERCA using a similar approach as for the comparison to the P_IB_-ATPase structures (Supplementary Table 2). The MmCadA structure displays the highest similarity to the [Ca_2_]E1ATP intermediate state of SERCA (PDB-ID: 1T5S or 3N8G)^47,57,59^. However, while the domain positions of MmCadA and the SERCA [Ca_2_]E1ATP state align relatively well overall, the A-domain is significantly displaced (~23 Å, ~91°, Fig. 1D). The divergence in domain arrangement between P_IB-2_-ATPases and SERCA likely reflects fundamental differences in intradomain communication. First, as noted above, the A/M1 linker is absent in P_IB_-type ATPases, rendering the A-domain less constrained (Supplementary Fig. 1). Second, the molecular size of the A-domain differs between P_IB_- and P_II_-type ATPases, altering the interacting interface with the P-domain (Fig. 1D). These factors likely contribute significantly to the unique orientation observed here in the E1 state. Importantly, the A-domain orientation seen in MmCadA has also been observed in P_IB-1_ ATPases^35^, suggesting that this positioning is likely characteristic of P_IB_-type ATPases in general (Fig. 1E).

The distinct A-domain location in P_IB-1_-ATPases has been proposed to provide space for interactions with Cu^+^-donating chaperones, which sequester cellular copper, since the corresponding A-domain position in SERCA would obstruct such binding. In contrast, P_IB-2_-type ATPases do not rely on such metal-binding proteins for ion supply, as Zn^2+^ is available at comparatively higher concentrations in cells. Instead, we propose that the unique position of the A-domain in the P_IB_-ATPases is potentially to minimize its influence on reshaping the M-domain conformational changes during the E1 and E2 transport cycle. This may explain why the M-domain appears more inert in P_IB_-type ATPases compared to P_II_-ATPases, where the ion uptake is orchestrated by movements of M1 and M2 (ref. ^57^). The distinct placement of the A-domain may also relate to positioning the MBDs in this subfamily. Alternatively, differences in cargo and nucleotide-binding events between P_IB_- and P_II_-ATPases may exist, partly due to the higher cellular abundance of Ca^2+^ compared to Zn^2+^. However, it has been demonstrated that SERCA turnover can be initiated by either Ca^2+^ binding followed by association of nucleotide, or the reverse^60^.

### Metal uptake via a wide electronegative pathway directly to the CPC-motif

Despite the shortage of structural information on metal-uptake and -binding in P_IB_-type ATPases, previous biochemical studies have identified a single high-affinity Zn^2+^ binding site^61,62^. The metal is likely coordinated by the cysteine thiolates of the conserved CPC motif of the M4 segment and two oxygen ligands from an invariant aspartate of M6, corresponding to C562, C564, and D889 in MmCadA (Supplementary Fig. 10a)^61,63^. In addition, a conserved lysine, equivalent to K868 in MmCadA, and located adjacent to D899, is critical for function but appears not to contribute to high-affinity ion coordination, as substitutions at this position leave the metal binding unaffected^20^. Nonetheless, it remains elusive how metal ions are delivered to the transmembrane binding site. For P_IB-1_-ATPases, an entry site coordinated by a conserved methionine has been suggested, providing a pathway from the intracellular environment to the high-affinity binding region^23^. In contrast, P_IB-2_-ATPases have been proposed to utilize a negatively charged funnel, gated by two conserved residues (M357 and F380 in MmCadA), which orchestrate metal uptake and release^20^. This funnel would be linked to the high-affinity binding residues via a conserved ion shuttling methionine, M357.

**Fig. 3:**
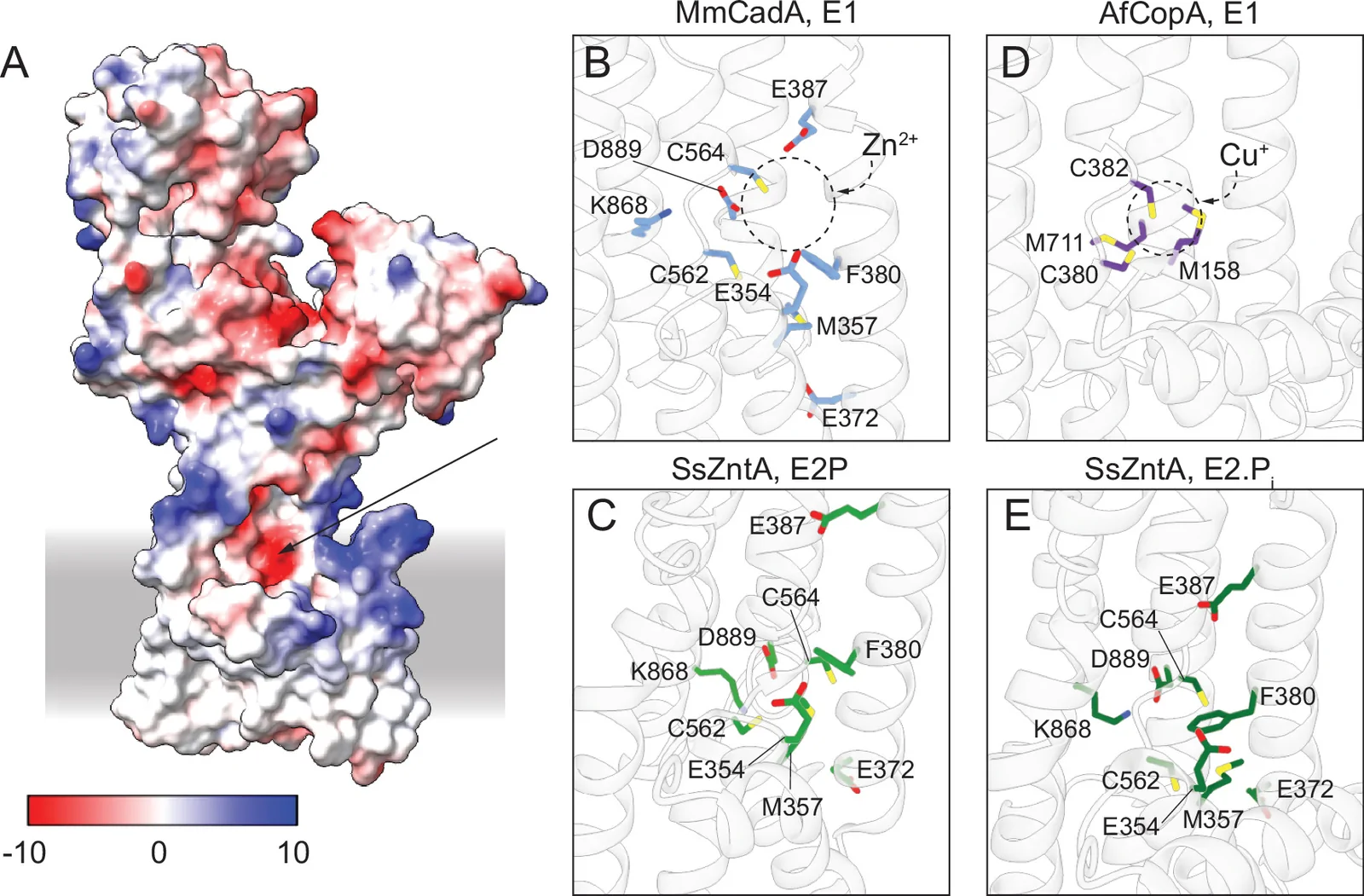
Details of Zn^2+^ uptake. **A** Surface charge of MmCadA reveals a highly electronegative entry region (arrow). The approximate location of the membrane is indicated. The range of electrostatic potential is measured in kcal/(mol*e) at 298 K (calculated in ChimeraX^70^). **B–E** cartoon close-views of the ion-uptake region during the E1 to E2.P_i_ transition of P_IB-2_-ATPases. Key functional residues (MmCadA numbering, except for AfCopA) are shown as sticks. The conserved CPC motif is exposed to the intracellular side in the E1 (MmCadA and AfCopA, PDB-ID 7R0I (ref. ^39^)), but not in the E2P (PDB-ID: 4UMV) and E2.P_i_ (PDB-ID: 4UMW) states^20^.

In our structure, we observe a wide, surface-exposed, electronegatively charged cavity lined by residues of M1, M2, and M4 (Fig. 3A). The pathway extends to the high-affinity metal-binding site, most prominently C564 of the CPC motif, which hence is directly exposed to the surrounding milieu (Fig. 3A and B). This arrangement contrasts with the E2P state of SsZntA, as can be expected with the shift from an inward-open to an outward-open configuration (Fig. 3C, E). However, the proposed gate between M357 and F380 is located far from the electronegative metal uptake region, and thus the MmCadA structure appears inconsistent with the funnel hypothesis (Fig. 3B). We suggest that these residues are not directly involved in metal uptake, a view supported by the minor functional effect of their substitution in SsZntA^20^. Instead, our structure indicates that C564 plays a primary role in ion acquisition, and likely provides the metal to C562 and D889 in subsequent E1 states.

Interestingly, we identify two additional residues capable of Zn^2+^ coordination, E354 (in M1) and E387 (M2), positioned peripherally to C564 at a distance of about 7 Å (Fig. 3B). As these are relatively well-conserved in P_IB-2_ and P_IB-4_-type ATPases (Supplementary Fig. 11), it is possible that they form a transition site for metal uptake in the electronegative pathway. This arrangement resembles but is not identical to the entry site proposed for LpCopA, where M148 (M1), E205 (M2), and D337 (M3) may coordinate Cu^+^ (Fig. 3D)^23^. However, we note that uptake of Cu^+^ typically relies on chaperones such as CopZ^64^ or ATOX1^65^ that sequester all ‘free’ copper, whereas no equivalent Zn^2+^ chaperones exist^20^. We therefore propose that P_IB-2_-ATPases employ a similar uptake mechanism to P_IB-1_-ATPases, but in a chaperone-independent manner.

K868, located adjacent to D889, has previously been suggested to promote metal release in the outward-open E2P state via charge repulsion, and to serve as an internal counter ion in the subsequent re-occluded E2.P_i_ state through charge complementation with D889^20^. Strikingly, the K868–D889 interaction is retained in our structure, despite the substantial conformational changes from the E2 and the E1 configurations (Fig. 3B–E, Supplementary Fig. 7). However, we speculate that the K868–D889 interaction is disrupted with the E1 to E1P shift associated with metal binding. There may be an indication of such a disruption in the determined structure, as the density for the K868 sidechain is poor in the B chain, indicating an increased flexibility (Supplementary Fig. 7j). Such a mechanism would directly couple metal uptake in the M-domain with ATP turnover in the soluble domains via M5, where K868 is located, and its direct connection to the P-domain.

### An N-terminus with multifaceted roles

**Fig. 4:**
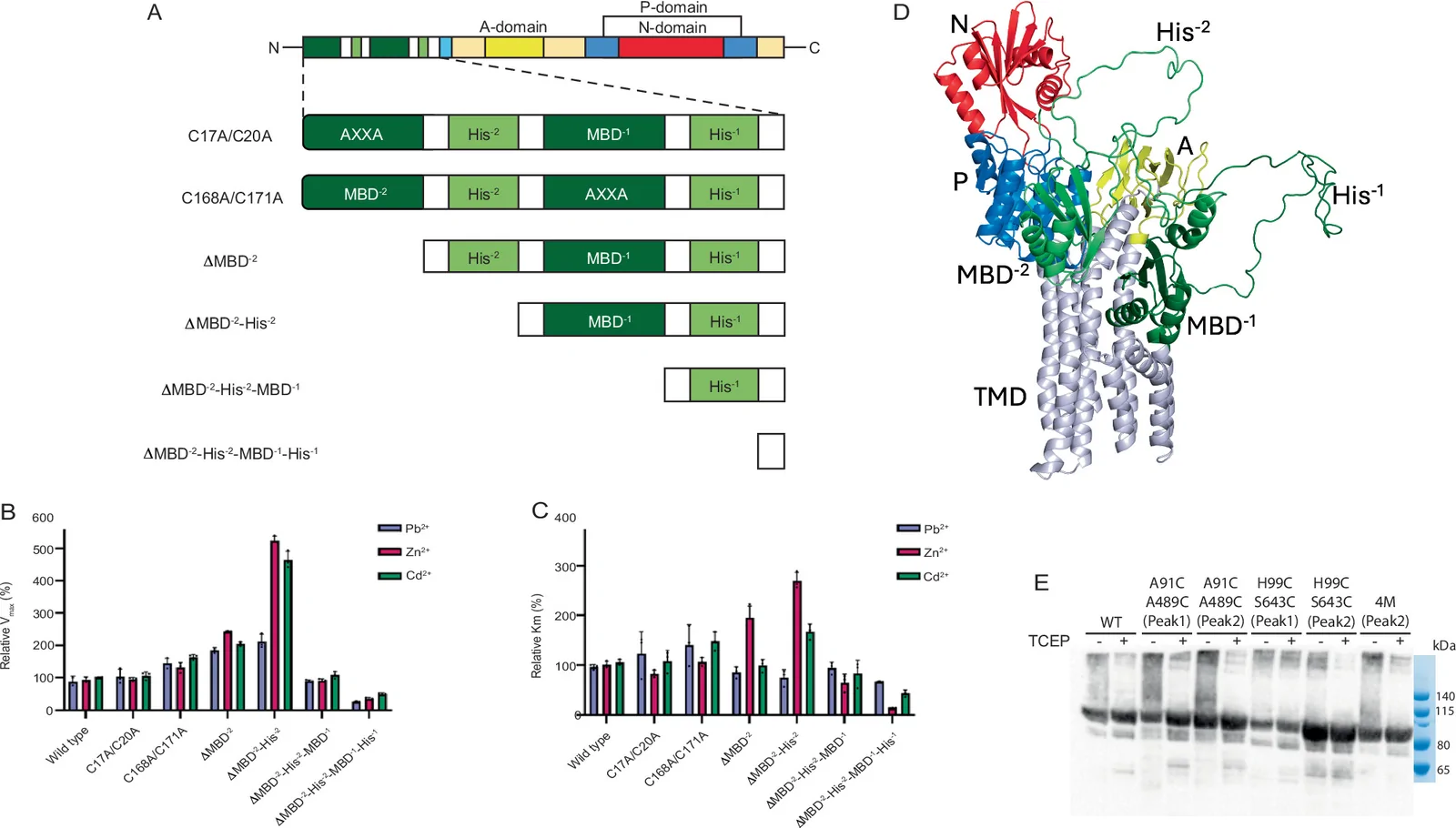
Functional analysis of the N-terminus. **A** MmCadA forms used for characterization. Relative ATPase activity (**B**) and metal affinity (**C**) of constructs in detergent-lipid solution, normalized to wild-type activity in the presence of Pb^2+^, Zn^2+^, or Cd^2+^. Data points in panels **B** and **C** represent three independent measurements using material from a single protein purification, and error bars represent the standard deviation. **D** A centroid MmCadA structure from the RMSD-based cluster corresponding to 20% of the molecular dynamics (MD) simulation frames. This structure corresponds to the frame with the lowest RMSD relative to all other frames in the cluster, highlighting the structural basis of autoinhibition. The A-domain is shown in yellow, the P-domain in blue, the N-domain in red, the MBD^−2^ and His^−2^ domains in light green, and the MBD^−1^ and His^−1^ segments in dark green. **E** Western blot of MmCadA with and without TCEP, to break cysteine bridges, with wild-type (WT) MmCadA, the double mutants A91C/A489C and H99C/S643C, as well as the quadruple mutant A91C/H99C/A489C/S643C (4M). The experiment was repeated twice using material from a single purification (different size-exclusion fractions). The SDS–PAGE ladder provides an approximate estimate of molecular weight.

As the N-terminus was unresolved in our structure, we opted to investigate its role using biochemical analysis, revisiting the functional assay previously employed for the wild-type enzyme (Fig. 1B). We generated a panel of MmCadA variants with incremental deletions (∆MBD^−2^, ∆MBD^−2^-His^−2^, and ∆MBD^−2^-His^−2^-MBD^−1^) as well as complete removal of the N-terminus (∆MBD^−2^-His^−2^-MBD^−1^-His^−1^). Separately, cysteine-to-alanine substitutions were introduced in MBD^−2^ (C17A/C20A) or in MBD^−1^ (C168A/C171A) (Fig. 4A and Supplementary Table 3). All N-terminally modified variants retained measurable activity and were stimulated by the same set of ions (Pb^2+^, Zn^2+^, and Cd^2+^) as the wild-type protein. However, ∆MBD^−2^-His^−2^-MBD^−1^-His^−1^ also exhibited low activity at high Cu^2+^ concentrations, hinting at a less stringent metal specificity (Fig. 4B, C and Supplementary Fig. 12). When removing only MBD^−2^-His^−2^ a shift in affinity and activity is seen, with lower *V*_max_ and *K*_m_ for Pb^2+^ (Fig. 4B, C). This indicates that the apparent affinity for Pb^2+^ may be too high for efficient transport, and does hint at a limited effect on metal specificity. Collectively, these results indicate that the N-terminus as a whole preceding the ATPase core is not essential for transport function, but it clearly modulates transport activity.

What, then, is the functional role of the N-terminus? Compared to the wild-type and even ∆MBD^−2^-His^−2^-MBD^−1^, deletion of the entire N-terminus (∆MBD^−2^-His^−2^-MBD^−1^-His^−1^) markedly reduces ATPase activity, while at the same time increasing the apparent affinity, most notably for Zn^2+^ (Fig. 4B, C). This highlights the functional importance of the histidine-rich segment located immediately upstream of the ATPase core (His^−1^). The elevated apparent metal affinity observed in ∆MBD^−2^-His^−2^-MBD^−1^-His^−1^ is most likely due to enhanced exposure of the high-affinity residues embedded in the ATPase core. Conversely, when this histidine stretch is retained (∆MBD^−2^-His^−2^-MBD^−1^), the activity and the affinity resemble those of the wild-type (Fig. 4B, C). One possible explanation is that the His^−1^ histidine segment, while having lower affinity than the high-affinity M-domain binding site, enables more efficient shuttling of metal to said high-affinity binding site. Physiologically, the histidines may thus serve to facilitate the higher activity seen in the wild-type compared to ∆MBD^−2^-His^−2^-MBD^−1^-His^−1^ and participate directly in metal delivery.

By contrast, deletion of MBD^−2^ (∆MBD^−2^) or MBD^−2^ together with its following histidine segment (∆MBD^−2^-His^−2^) increases the activity 2–5-fold relative to wild-type (Fig. 4B). This stimulation is independent of the canonical CXXC-motif classically considered integral to the function of these domains, as the C17A/C20A mutant behaves similarly to wild-type (Fig. 4B, C). We interpret these data as indicating that MBD^−2^ and, in particular, its adjacent histidine segment (His^−2^) have a structural role in inhibiting metal transport, perhaps through binding to the ATPase core structure, interfering with rearrangement of the soluble domains and/or metal transfer to the ATPase core. We note that such a CXXC-independent role of an MBD has been proposed previously, for example, for MBD^−1^ of OsHMA4 (where the CXXC-motif is even missing), although in OsHM4 the MBD^−1^ serves a different structural and functional role^35^.

How, then, is this autoinhibition relieved? We find that compared to ∆MBD^−2^-His^−2^ and ∆MBD^−2^-His^−2^-MBD^−1^-His^−1^, ∆MBD^−2^-His^−2^-MBD^−1^ displays reduced and increased activity, respectively (Fig. 4B, C). This supports a stimulatory role for MBD^−1^, although the modest effect of mutating its CXXC motif (C168A/C171A) indicates MBD^−1^ operates in a CXXC-independent manner, similar to what we proposed for MBD^−2^. We suggest that metal sensing and delivery are mediated by the histidine stretch adjacent to the ATPase core (His^−1^), triggered by augmented local metal concentrations. MBD^−1^ amplifies the sensing effect of His^−1^, facilitating the release of autoinhibition exerted by MBD^−2^ and its associated histidine stretch (His^−2^). At low Zn^2+^ levels, the distal N-terminal modules (MBD^−2^ and His^−2^) suppress transport activity, whereas at higher concentrations this inhibition is lifted, allowing uptake via the histidine stretch next to the ATPase core. Moreover, the increased *K*_m_ for Zn^2+^ and Cd^2+^ when MBD^−2^-His^−2^ is removed, despite the improved activity, hints that this part is involved in metal handling in the wild-type protein.

### A structural model for the autoinhibition

To elucidate the structural basis of autoinhibition, a structural model of full-length MmCadA was generated using AlphaFold (Supplementary Fig. 6). The predicted aligned error (PAE) indicated good overall confidence for the structure, except for the His^−1^ and His^−2^ linker regions (Supplementary Fig. 13a). To assess the prediction of the flexible linker regions, we performed five independent AlphaFold predictions (seeds) and selected the top-ranked model from each run for comparison. The resulting pLDDT (predicted Local Distance Difference Test) showed similar scores for the His^−1^ and His^−2^ linker regions (Supplementary Fig. 13b), and their RMSD varied within 5 Å (Supplementary Fig. 13c), which indicates inter-model consistency despite their inherent flexibility. The best-scoring MmCadA model adopted an E1-configuration comparable to our MmCadA structure (RMSD 2.3 Å), with only minor displacements of the A- and N-domains (Supplementary Fig. 14a), and indicating flexibility of the N-terminal region through low confidence scores and lack of structure in the histidine-rich segments. In this model, MBD^−1^ is associated with MB’ and the soluble segments of M2 and M3 adjacent to the proposed ion-uptake region, whereas His^−1^, MBD^−2^, and His^−2^ were positioned more peripherally to the ATPase core (Fig. 4D, Supplementary Fig. 14b).

We next used this full-length wild-type MmCadA model and a mutant ∆MBD^−2^-His^−2^ truncation (lacking MBD^−2^ and its associated histidine-rich segment) to perform replica-exchange molecular dynamics simulations in lipid bilayers and solvated in the absence of metal. The simulation results showed extensive loss of contacts (defined as atom pairs within 3.5 Å) between the N-terminal regulatory region and the A- and P-domains upon ∆MBD^−2^-His^−2^ truncation (Supplementary Fig. 14c, e), while contacts to the N-domain were relatively transient for the wild-type protein and absent for the mutant (Supplementary Fig. 14g). The RMSD showed an approximately similar trend in both the wild-type and the ΔMBD^−2^-His^−2^ truncation, with only minor differences between them (Supplementary Fig. 14d, f, h); however, RMSF analysis revealed higher residue-level fluctuations in the ΔMBD^−2^-His^−2^ truncation compared to the wild-type.

The overall flexibility of the N- and A-domains increases upon the ΔMBD^−2^-His^−2^ truncation; removal of these stabilizing contacts allows greater flexibility of both domains (Supplementary Fig. 14i, j). This increased flexibility presumably improves the efficiency of the catalytic cycle, leading to enhanced ATP hydrolysis activity. The simulations were supported by double cysteine mutations. The A91C/A489C mutant (located on the surface interface between the His^−2^ and A-domains) showed increased smearing on native PAGE western blot in the absence of TCEP, indicating interaction between these two regions. In contrast, no similar smearing was observed for the H99C/S643C mutant (His^−2^/P-domains), but was seen in the quadruple mutant (A91C/H99C/A489C/S643C) (Fig. 4E).

Taken together, these results support a model in which the distal N-terminal region (MBD^−2^-His^−2^) inhibits ATPase activity by constraining the cytosolic domains through stabilizing contacts. Truncation of this region releases these restraints, allowing greater flexibility and thereby stimulating catalysis. However, it should be noted that the predictive models for His^−1^ and His^−2^ in the N-terminal region have low confidence scores; consequently, any interpretations involving these residues should be made with caution.

## Discussion

**Fig. 5:**
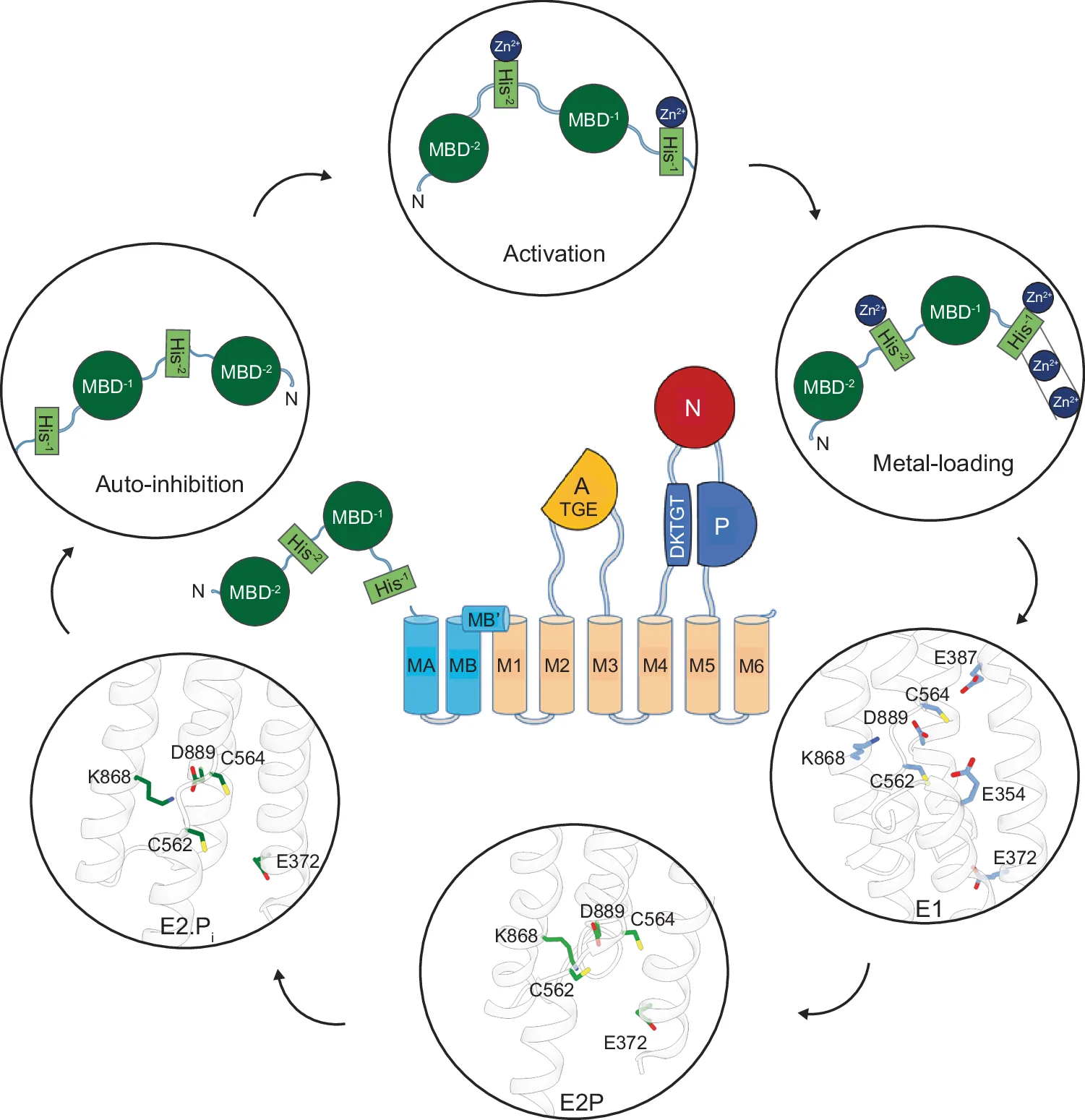
Proposed regulation and ion uptake mechanism. *Auto-inhibition*: The first N-terminal part sequesters Zn^2+^ and autoinhibits ATPase turnover, likely by interfering with the soluble domains of the ATPase core. *Activation*: At elevated metal levels, a histidine stretch in immediate connection to the ATPase core (His^−1^) acts as a sensor, releasing the N-terminus from the core to activate transport, a process that is assisted by MBD^−1^. *Metal-loading*: His^−1^ aids metal delivery through the electronegative uptake region, lined by E354, E387, and C564. E1: C564 of the CPC-motif is exposed to the intracellular milieu and accepts the incoming metal. In the subsequent E1P intermediate, high-affinity metal binding in the M-domain is established by C562, C564, and D889. E2P: The outward-facing configuration permits metal release, stimulated by K868 with subsequent transfer via E372. E2.P_i_: K868 reorients towards D889, serving as an internal counterion to charge stabilize D889 in the absence of metal. Once intracellular metal levels normalize, auto-inhibition is restored.

In this work, we present the inward-open structure of a P_IB-2_-type ATPase, complemented by functional analysis. A Zn^2+^ uptake pathway from the intracellular milieu is proposed, which includes involvement of the N-terminal tail, preceding the ATPase core, as well as the M-domain (Fig. 5). At low metal levels, the most N-terminal portion (MBD^−2^ and His^−2^) interferes with the ATPase turnover, likely through interaction with the A- and N-domains, primarily in a CXXC-independent manner. At elevated metal levels, the first portion of the N-terminus (MBD^−2^ and His^−2^) is released, as induced by the binding to metal-sensing His^−1^ and amplified by MBD^−1^ in a CXXC-independent manner. The release of the N-terminal tail permits the histidine stretch adjacent to the ATPase core (His^−1^) to assist in the metal-loading process, analogously to a metallochaperone.

Once the metal ions reach the M-domain, the exposed C564 of the CPC-motif likely represents the initial metal binding residue, perhaps assisted by E354 and E387, or the latter may establish a transient uptake site. In the next step, along with the formation of the E1P state, it can be expected that D889 is liberated from K868, and that the high-affinity ion binding site is formed between C562, C564, and D889. ATP hydrolysis triggers rearrangement of the N- relative to the P-domain, which leads to the outward-open configuration, as has been revealed previously, and metal extrusion is perhaps stimulated by K868 and E372^20^. Next, the protein becomes re-occluded with the transition to the E2.P_i_ intermediate in which K868 rebinds to D889, presumably serving as an internal counter-ion, charge stabilizing the M-domain in late E2 stages. Eventually, with normalized intracellular metal concentrations because of numerous ATP cycles, the N-terminal tail will re-autoinhibit the ATPase core. Complementing structures of E1P and E2 states will be necessary to further resolve how ion specificity and the complete transport mechanism are achieved.

Putting our findings in a larger context, the repertoire of proposed functional roles attributed to the MBD-bearing tails surrounding the catalytic core of P_IB_-ATPases is steadily expanding. These domains have been implicated in a remarkable variety of tasks: from directing subcellular localization and trafficking in eukaryotes, to imposing autoinhibition in order to suppress unwanted turnover, to functioning as sensors that can relieve such inhibition, to actively participating in metal transfer pathways, or alternatively playing purely structural roles, such as expanding the A-domain, conceptually paralleling the so-called NTD of P_V_-ATPases, or in amplification of certain of these processes, as suggested here for MmCadA^66^.

Traditionally, the presence of metal-binding CXXC motifs was thought to be a defining hallmark of MBDs, conferring essential functionality. Yet this notion has proven overly rigid. Increasing evidence demonstrates that several MBD-associated roles can be carried out independently of direct metal coordination. This is underscored by the fact that not all MBDs contain the canonical CXXC residues, and yet they remain functionally relevant, as for example observed for MBD^−1^ of HMA4. Most MBDs indeed adopt a glutaredoxin-like fold, but nature has produced a diverse spectrum of MBD types, and their complexity is heightened further by the sequences flanking them - including the linkers between consecutive MBDs - which themselves may contribute to metal handling, as is also proposed here^13^.

Studies on ATP7B and on rice HMA4 illustrate how multiple functional dimensions can be integrated within a single polypeptide chain: enlargement of the A-domain through MBD^−1^, likely mediation of metal delivery by MBD^−2^, and autoinhibitory functions through MBD^−3^ (and perhaps additional downstream MBDs)^35,38,40^. On top of that, subcellular localization signals may also be encoded within these tails, though the precise contribution of individual MBDs to this process remains unresolved. Moreover, it cannot be ruled out that certain tail segments combine multiple activities. In contrast, work on the bacterial model protein LpCopA has revealed a somewhat different organizational logic. There, the atypical MBD^−1^ acts primarily as a metal sensor, while its preceding tail region autoinhibits the pump^41^. These findings highlight the architectural and functional plasticity of MBD-containing tails.

For MmCadA, evidence suggests that many of the above-mentioned functions are bundled into its N-terminal region. Intriguingly, these activities appear largely independent of the canonical CXXC motifs found in its two MBDs. Instead, histidine-rich sequences flanking the MBDs take center stage: His^−2^ plays a key role in autoinhibition, whereas His^−1^ is crucial for metal delivery, especially noteworthy considering the high flexibility of these regions. Thus, we are only beginning to unravel the true mechanistic depth of these tails. Even MBDs that appear similar can fulfill very different roles depending on context. This is evident, for instance, when comparing MBD^−1^ of MmCadA with the corresponding MBDs in eukaryotic copper transporters. In eukaryotes, both MBD^−1^ and MBD^−2^ are indispensable for proper function, whereas many prokaryotic P_IB_-ATPases appear capable of functioning with fewer MBDs or even without them altogether. Nevertheless, in MmCadA, His^−2^ emerges as relatively important, hinting at a nuanced functional spectrum rather than a binary requirement.

Whether the same overarching principles govern both prokaryotic and eukaryotic systems also remains unresolved. Given that prokaryotic P_IB_-ATPases possess far fewer MBDs*—*or even none*—*it would be reasonable to speculate that they contribute less to overall ATPase activity in these organisms. However, the multifunctional roles of MBDs are likely fine-tuned by a wide range of factors: the particular fold type (e.g., ferredoxin-like), the phase of the ATPase catalytic cycle, the chemical and structural environment, the length and flexibility of linkers connecting MBDs both to each other and to the ATPase core, as well as the three-dimensional spatial relationships between the ensemble of MBDs and the ATPase machinery. These features have likely co-evolved with the ATPase core, ensuring functional adaptation to diverse ecological and cellular contexts.

At present, structural insights into the placement of MBDs relative to the ATPase core remain sparse. No high-resolution data are available for MBD^−6^ through MBD^−3^ in eukaryotic P_IB_-ATPases, nor for any MBDs in prokaryotic systems. Consequently, downstream structural and mechanistic studies will be indispensable for fully dissecting how the enigmatic tails contribute to metal transport and regulation in P_IB_-ATPases. Nevertheless, our results help to close a substantial gap in the current understanding of how P_IB_-type ATPases orchestrate the uptake, handling, and regulatory control of transition-metal ions. By shedding light on these molecular principles, the work not only advances fundamental knowledge but also opens the door to applied perspectives. In particular, such insights may be harnessed in biotechnology—for instance, in the rational design of plants or bacteria with enhanced hyperaccumulation capacity. These engineered organisms could serve as tools for phytoremediation and microbial bioremediation strategies of contaminated environments. More broadly, unraveling these mechanisms may inspire innovative approaches to biogeochemical cycling and green technologies aimed at reducing the ecological footprint of metal pollution.

## Methods

### Overproduction and purification of MmCadA

Five P_IB-2_-type ATPase genes, each encoding a variable number of MBDs, were synthesized with an N-terminal Strep-tag and introduced into the pET52b(+) vector using SacI and KpnI restriction sites (Supplementary Fig. 2). The constructs were transformed into *E. coli* BL21-CodonPlus(DE3) cells, and expression was assessed by western-blotting using strep-tag antibody, which identified MmCadA as a promising research target (Supplementary Fig. 3). MmCadA point mutations and truncations were achieved using the QuikChange Lightning Site-Directed Mutagenesis Kit (Agilent) or the Phusion Site-Directed Mutagenesis Kit (Thermo Fisher Scientific). Primers were designed to introduce the following modifications (C17A/C20A, C168A/C171A, (∆MBD^−2^, ∆MBD^−2^-His^−2^, and ∆MBD^−2^-His^−2^-MBD^−1^ and ∆MBD^−2^-His^−2^-MBD^−1^-His^−1^) (Supplementary Table 4). All wild-type, mutant, and truncated constructs were verified by DNA sequencing.

For protein expression, cells were cultured in LB medium at 37 °C until reaching an optical cell density (OD_600nm_) of 1, induced with 0.5 mM IPTG, and further incubated for 20 h at 16 °C. Cells were harvested by centrifugation at 6000×*g* for 15 min, resuspended in buffer (20 mM Tris–HCl, pH 7.5, 200 mM KCl, 20% (v/v) glycerol, 5 mM β-mercaptoethanol (βME)) at 5 mL/g wet cell weight, and stirred for 16 h at 4 °C. DNAase I (5 μg/mL), MgCl_2_ (1 mM), and Sigma protease inhibitor tablets (1 per 6 L culture) were added to the cell suspension. Cells were disrupted by two passes through an Avestin high-pressure homogenizer at 25,000 p.s.i., with 1 mM phenylmethylsulphonyl fluoride (PMSF) added immediately after lysis. Cell debris was cleared by centrifugation at 20,000×*g* for 1 h, and membranes were isolated by ultracentrifugation at 185,000×*g* for 3 h. Membranes were resuspended using a homogenizer in solubilization buffer (20 mM MOPS–KOH, pH 7.5, 100 mM KCl, 20% (v/v) glycerol, 3 mM MgCl_2_, 5 mM βME, and 1 mM PMSF) at 1 mL buffer per 0.2 g wet membrane weight. The suspension was diluted with an equal volume of solubilization buffer containing 2% (w/v) octaethylene glycol monododecyl ether (C_12_E_8_) detergent, and stirred for 2 h at 4 °C. Insoluble material was removed by ultracentrifugation at 185,000×*g* for 1 h.

The C_12_E_8_ solubilized membrane extract was loaded twice onto a StrepTactin XT superflow high-capacity 5 mL prepacked column (IBA Lifesciences), pre-equilibrated with Strep buffer (20 mM MOPS–KOH, pH 8.0, 200 mM KCl, 20% (v/v) glycerol, 3 mM MgCl_2_, 5 mM βME, 0.015% (w/v) C_12_E_8_). Bound protein was eluted using Strep buffer also containing 50 mM biotin. Eluted fractions were assessed by SDS–PAGE, and the protein was concentrated to ~10 mg/mL using Vivaspin 20 concentrators (MWCO 50 kDa). For final purification and buffer exchange, 10 mg of concentrated protein was applied onto a Superose 6 size-exclusion column pre-equilibrated with SEC buffer (20 mM MOPS–KOH, pH 8.0, 80 mM KCl, 20%(v/v) glycerol, 3 mM MgCl_2_, 5 mM βME, 0.015% (w/v) C_12_E_8_). Fractions containing MmCadA were pooled, concentrated to 3 or 10 mg/mL (see below) using Vivaspin 6 tubes (MWCO 50 kDa), flash-frozen in liquid nitrogen, and stored at −80 °C for downstream efforts.

### In vivo metal challenge assays

*E. coli* BL21-Gold(DE3) cells were transformed with plasmids encoding wild-type MmCadA, the inactive mutant D606N (substitution of the catalytic aspartate required for autophosphorylation in the Post-Albers cycle), or a control vector (expressing no protein). Transformants were cultured overnight at 37 °C in LB media supplemented with 50 μg/mL ampicillin. Overnight cultures were diluted 100-fold into fresh LB media containing ampicillin and grown at 37 °C to OD_600_ ≈ 2.0. Protein expression was induced by the addition of 0.5 mM IPTG, followed by incubation at 16 °C for 20 h. Prior to inoculation into final assays, cultures were normalized to OD_600_ ≈ 2.0 to ensure comparable initial cell densities across all conditions. Expression cultures were then subjected to two assays: (i) metal susceptibility and (ii) intracellular zinc quantification. For the metal susceptibility assay, cells were inoculated at 10% (v/v) into 96-well plates containing LB media supplemented with ampicillin, 0.5 mM IPTG, and varying concentrations of ZnCl_2_ (0–3 mM), CdCl_2_ (0–1 mM), or Pb(CH_3_COO)_2_ (0–10 mM). Cell growth (OD_600_) was monitored every 10 min for 9 h at 37 °C using a plate reader (Tecan Spark 20 M). To compare phenotypic differences between constructs, OD_600_ values at 5 h were analyzed. Each condition was measured in three technical replicates, and statistical significance was evaluated using an unpaired *t*-test relative to the control vector. Second, for the intracellular zinc quantification assay, cultures were inoculated at 10% (v/v) into pre-weighed tubes containing 25 mL LB media supplemented with ampicillin, 0.5 mM IPTG, and ZnCl_2_ (0–2 mM). Cells were grown for 48 h at 16 °C with shaking. To remove excess extracellular zinc, cells were washed three times by cell harvesting by centrifugation (4000×*g* for 20 min at 4 °C) followed by resuspension in 20 mL metal-free LB media. After a final centrifugation step, pellets were dried overnight in a vacuum chamber, and cell mass was determined by subtracting the tube weight. Dried cell pellets were digested in 25% (v/v) nitric acid and incubated at 80 °C overnight, and subsequently diluted to 3% (v/v) nitric acid for analysis. Zinc content was quantified via inductively coupled plasma mass spectrometry (ICP-MS, Agilent 7900 ICP mass spectrometer connected to a CETACASX-500 autosampler for sample injection). Intracellular zinc levels were normalized to cell mass and reported as μg of Zn per mg of cells. Three biological replicates, each with three technical replicates, were recorded per condition, and statistical significance was assessed with an unpaired *t*-test relative to the control vector.

### State stabilization, crystallization, and data processing

MmCadA (100 μL, 10 mg/mL) was supplemented with 4 μL C_12_E_8_ (100 mg/mL) and incubated for 16 h at 4 °C. The mixture was clarified by centrifugation at 20,000×*g* for 15 min and diluted to 8 mg/mL in SEC buffer. The diluted protein was incubated with Pb(CH_3_COO)_2_ at a final concentration of 2.5 mM, kept on ice for 30 min, and centrifuged again at 20,000×*g* for 15 min. Subsequently, AMPPNP and MgCl_2_ (3 and 1 mM final concentration, respectively) were added, incubated with the protein sample for 30 min, and the sample was centrifuged once more at 20,000×*g* for 20 min.

Crystallization trials were set up by mixing 1 μL of the prepared MmCadA solution and 1 μL of reservoir solution on siliconized cover slides for a final protein concentration of 4 mg/mL. Crystals grew for 2−4 weeks at 18 °C. The highest-quality crystals were obtained from reservoirs containing 30% (v/v) Jeffamine and 0.1 M sodium citrate, pH 5.2. Crystals were harvested using mounted litholoops, flash-cooled in liquid nitrogen, and screened at synchrotron sources. Final diffraction data were collected at the Swiss Light Source (Switzerland) on the X06SA beamline. Data were processed using XDS (Supplementary Table 1), with model building and refinement performed iteratively using Phenix refine^67^ and Coot^68^. Figures were generated using Pymol^69^ and ChimeraX^70^.

### Functional characterization

A series of N-terminal constructs (C17A/C20A, C168A/C171A, ∆MBD^−2^, ∆MBD^−2^-His^−2^, and ∆MBD^−2^-His^−2^-MBD^−1^ and ∆MBD^−2^-His^−2^-MBD^−1^-His^−1^) were expressed and purified following the same protocol as for full-length wild-type MmCadA. For functional characterization, all constructs were concentrated to 3 mg/mL, flash-frozen in liquid nitrogen, and stored at −80 °C until use.

ATPase activity was measured using the so-called Baginski assay, which quantifies inorganic phosphate released during metal-stimulated ATP hydrolysis^42^. In brief, 50 μL of reaction buffer (40 mM MOPS–KOH, pH 8.0, 150 mM NaCl, 80 mM KCl, 3 mM MgCl_2_, 20 mM (NH_4_)_2_SO_4_, 20 mM L-cysteine, 0.25 mM Na_2_MoO_4_, 1.2 mg/mL soybean lipids, 3 mg/mL C_12_E_8_) was mixed with MmCadA at 0.04 mg/mL. The reaction was initiated by the addition of 3 mM ATP and incubated at 37 °C for 10 min. The reaction was terminated by adding 50 μL of freshly prepared stop solution (2.5% (w/v) ascorbic acid, 0.4 M HCl, 0.48% (NH_4_)_6_Mo_7_O_24_, 0.8% SDS), followed by 10 min incubation. Subsequently, 75 μL of arsenic solution (2% (w/v) arsenite, 2% (v/v) acetic acid, 3.5% (w/v) sodium citrate) was added, and absorbance was measured at 860 nm.

Under these assay conditions, the metal ion specificity and selectivity of MmCadA were assessed with Pb^2+^, Zn^2+^, Cd^2+^, Hg^2+^, Cu^+^/Cu^2+^, Co^2+^, and Ni^2+^, across a concentration range of 0–2 mM (0, 0.05, 0.1, 0.25, 0.5, 1, and 2 mM). The *V*_max_ values of the mutated and truncated MmCadA variants were determined in the presence of Pb^2+^, Zn^2+^, or Cd^2+^ at 0.05, 1, and 2 mM, keeping the molar protein concentration constant. Free phosphate release was quantified against a standard curve generated from phosphate concentrations.

### Blue native PAGE and western blot

All mutants were generated through overlap PCR with individual primer pairs (Supplementary Table 4). The plasmid was transformed into the C43 (DE3) *E. coli* strain. Several colonies were inoculated in 10 mL LB medium supplied with 100 µg/mL ampicillin at 37 °C with shaking at 180 rpm for 3 h. The preculture was then transferred into a 500 mL TB culture supplied with 2 mM MgCl_2_, 100 µg/mL ampicillin, 0.4% glycerol (v/v), and incubated at 37 °C with shaking at 180 rpm. Protein expression was induced with 0.8 mM isopropyl β-D-1-thiogalactopyranoside at 20 °C for 20 h, when OD_600nm_  =  0.6. The cells were harvested through centrifugation at 6000×*g* for 30 min. The cell pellets were then washed with buffer and resuspended with lysis buffer (25 mM Tris, pH = 7.5, 150 mM NaCl, 5% (v/v) glycerol, 5 mM EDTA). The cells were disrupted by X-press high pressure, and crude membranes were collected through ultracentrifugation at 160,000 × g for 1 h. The pelleted membranes were solubilized at 18 °C for 30 min in the presence of buffer A (50 mM Tris pH = 7.5, 150 mM NaCl, 10% (v/v) glycerol, 1 mM EDTA) supplemented with 1% (w/v) DDM. The insoluble material was removed by ultracentrifugation at 190,000×*g* for 30 min. The supernatant was loaded on a prepacked 5 mL Streptrap XT prepacked column (Cytiva), equilibrated with 30 mL buffer A containing 0.03% (w/v) DDM. Contaminations and unbound protein were removed through the washing of the column with 10 column volumes of buffer A, supplemented with 0.03% (w/v) DDM. The target protein was eluted with 1× Buffer E (Strep-Tactin elution buffer with desthiobiotin, IBA) diluted with buffer A containing 0.03% (w/v) DDM. Fractions containing the target protein were pooled and concentrated using 100 kDa MWCO concentrators (Sigma). The sample was further polished using size-exclusion chromatography (SEC) using a Superose 6 column (Cytiva) equilibrated with buffer (50 mM HEPES, pH = 7.5, 150 mM NaCl, 10% (v/v) glycerol, 0.02% (w/v) C_12_E_8_). The peak fractions were used for downstream applications individually. The protein purity was verified using SDS–PAGE.

WT SEC peak fractions were loaded onto a 4–16% Bis–Tris Mini Native PAGE gel (Invitrogen) for analysis. Peak fractions were diluted to 0.1 mg/mL in SEC buffer supplemented with or without 5 mM TCEP. For each sample, 1  μg protein was loaded onto 4–12% Bis–Tris SDS–PAGE gel (Invitrogen) and run for 50 min. Proteins were transferred to a membrane using the iBlot^TM^ 3 Western Blot Transfer Device. Immunoblotting was performed using the iBand^TM^ Flex solution kit (Invitrogen) with a Strep-Tag II monoclonal antibody (HRP-conjugated; Cat. No. BSM-33166M-HRP).

### Molecular dynamics simulations

To assess the consistency of the AlphaFold model (ID: I4IY19) with other AlphaFold predictions, an ensemble of additional structures was generated using five independent prediction runs. For each run, the best-ranked model (ranked 0) was selected for comparison. These predictions utilized all available databases to generate deep multiple sequence alignments, maximizing the evolutionary information for structure prediction. No templates were used, ensuring that the resulting models were primarily guided by sequence-derived features. The results were analyzed to evaluate the reproducibility of the predicted structure, with particular attention to regions of lower confidence.

The AlphaFold model of wild-type MmCadA was retrieved from UniProt (ID: I4IY19)^71^. To generate the ∆MBD/His^−2^ variant, the N-terminal segment containing MBD^−2^ and its associated histidine-rich region (His^−2^) was removed. Both the wild-type and the truncated structures were then embedded in lipid bilayers containing 915 POPC molecules using the CHARMM-GUI bilayer builder module^72^. The wild-type system contained 154,765 TIP3P water molecules, 438 K^+^ ions, and 426 Cl^−^ ions, corresponding to ~0.15 M KCl, with an additional 12 K⁺ ions added to neutralize the system; the simulation box dimensions were 18.01 × 18.01 × 19.57 nm. The ∆MBD/His^−2^ system contained 146,296 TIP3P water molecules, 408 K^+^ ions, and 403 Cl^−^ ions, corresponding to ~0.15 M KCl, with the excess 5 K^+^ ions added for charge neutralization; the simulation box dimensions were 18.01 × 18.01 × 18.69 nm^73^. A summary of the molecular dynamics simulation setup and parameters is provided in Supplementary Table 5.

All molecular dynamics (MD) simulations were performed with GROMACS 2023.3 (ref. ^74^). For both the wild-type and the truncation, three-replica exchange simulations were carried out for 500 ns each. The systems were energy minimized using the steepest-descent algorithm until the maximum force on any atom was <1000 kJ mol^−1^ nm^−1^, followed by a six-step equilibration process with progressively reduced atom-position restraints. The first two equilibration stages (125 ps each, 1 fs time step) were carried out under constant temperature (303.15 K) and volume (NVT ensemble). The following four steps were run under constant temperature (303.15 K) and pressure (1 bar, NPT ensemble): step three for 125 ps with a 1 fs time step, and steps four to six for 500 ps each with a 2 fs time step. Backbone RMSD, potential energy, temperature, and pressure across the six equilibration stages are shown in Supplementary Fig. 15.

To improve sampling, three-replica exchange simulations were conducted at narrowly spaced temperatures (303.15–303.25 K)^75^ and 1 bar using a V-rescale thermostat and a C-rescale barostat. Long-range electrostatic interactions were treated with the smooth particle mesh Ewald (PME) method using a 12 Å cutoff^76,77^, and bond constraints involving hydrogen atoms were enforced with the LINCS algorithm^78,79^. Trajectory analysis was carried out with GROMACS 2023.3^74^, while structural visualization and plotting were performed using PyMOL and Python^80^. Clustering analysis of the simulation trajectory was performed using an RMSD cutoff of 0.2 nm. The largest cluster was identified as the most populated conformational state (20% of MD frames). The representative structure of this cluster was defined as the frame with the minimum average RMSD relative to all other frames within the cluster. This structure was used for visualization (Fig. 4D). For the MD simulations checklist, please see Supplementary Table 5.

### Reporting summary

Further information on research design is available in the Nature Portfolio Reporting Summary linked to this article.

## Supplementary information

Supplementary Information

Reporting Summary

Transparent Peer Review file

## Source data

Source Data

## Data Availability

The data that support this study are available from the corresponding author upon reasonable request. The atomic coordinates have been deposited in the Protein Data Bank (PDB) under accession codes 9T44. The following published structures were used for comparisons: 4H1W, 2C9M, 3N8G, 1T5S, 1T5T, 3BA6, 3B9B, 3N5K, 3FGO, 3NAL, 4BBJ, 3RFU, 4UMV, 4UMW and 7R0I. All files required for producing the trajectories of the MD simulations can be found in Zenodo [https://doi.org/10.5281/zenodo.21476325]. The source data underlying Figs. 1, 4, and Supplementary Fig. 5 as well as uncropped gels and blots are provided as a Source Data file. Source data are provided with this paper.
