# Supplementary material for: Structural and functional insights into a Zn2+-transporting P-type ATPase
Source: Nat Commun. 2026 Sep 16;17:10886. doi: 10.1038/s41467-026-77558-1 (PMC13677743; doi:10.1038/s41467-026-77558-1)
Supplement: Supplementary file 1 [file 41467_2026_77558_MOESM1_ESM.pdf]

Supplementary information for:

Structural and functional insights into a Zn^{2+}-transporting P-type ATPase

Pin Lyu[1], Elena Longhin[1], Fatemeh Sabzian-Molaei[2], Fernando Montalvillo Ortega[3], Ping Li[4], Kaituo Wang[1], Annette Duelli[1], Tamim Al-Jubair[1], Hajira Ahmed Hotiana[1], Eva Ramos Becares[1], Tristan Croll[5], Gabriele Meloni[3], Magnus Andersson[2], Viktoria Bågenholm[1,4] & Pontus Gourdon[1,4*]

[1] Department of Biomedical Sciences, University of Copenhagen, Copenhagen, Denmark

[2] Department of Chemistry, Umeå University, Umeå, Sweden

[3] Department of Chemistry and Biochemistry, University of Texas at Dallas, Richardson, TX, USA

[4] Department of Experimental Medical Science, Lund University, Lund, Sweden

[5] Department of Haematology, University of Cambridge, Cambridge, UK

[*] Correspondence to: PG (pontus.gourdon@med.lu.se)

Supplementary Fig. 1.

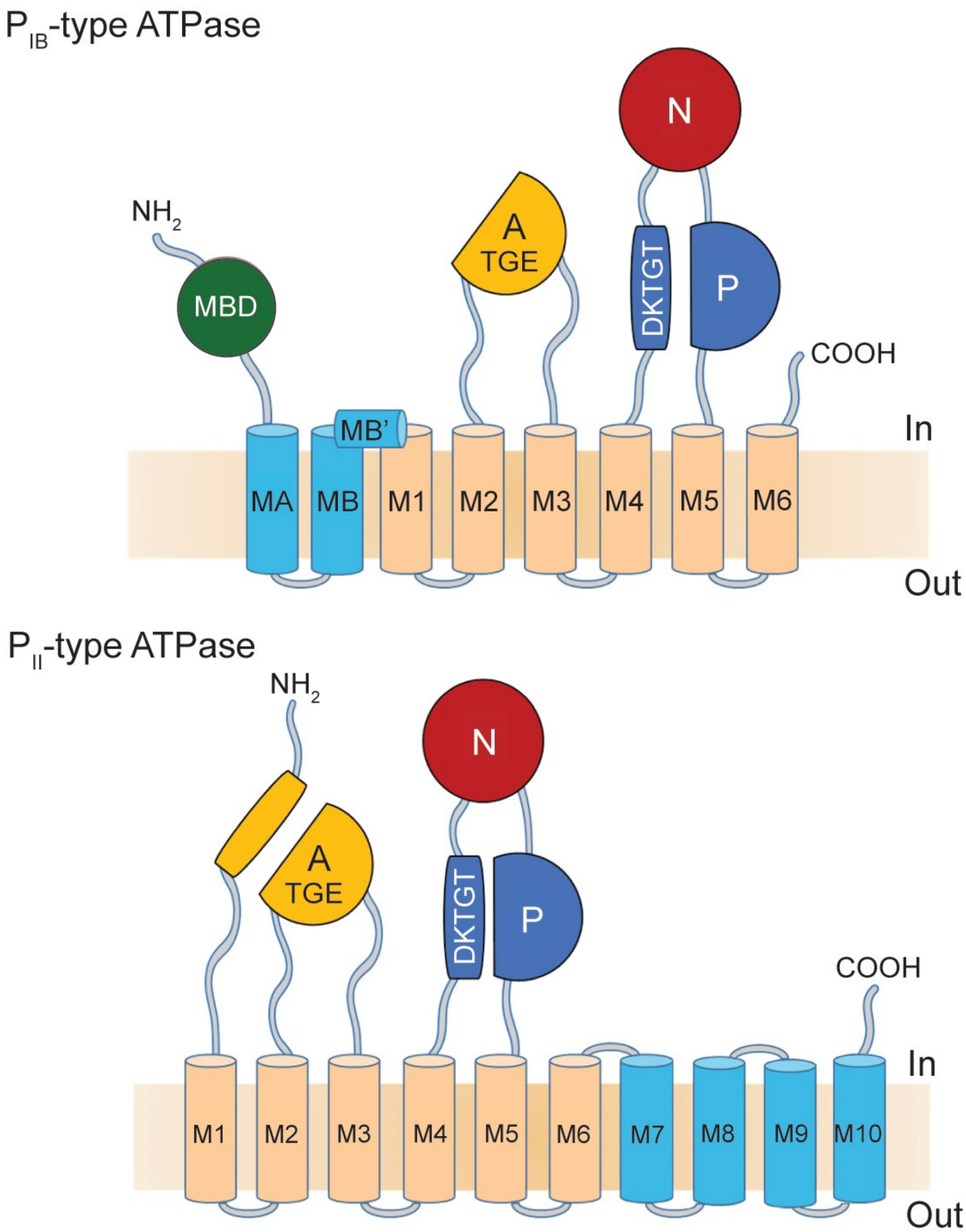

Supplementary Figure 1. Topology of P_{IB}- and P_{II}-type ATPases. The conserved cytosolic A-, P-, and N-domains are shown in yellow, blue, and red, respectively, while the core transmembrane helices are depicted in wheat and additional transmembrane helices unique to each family are colored cyan (MA-MB-MB' for P_{IB}-ATPases and M7-M10 for P_{II}-ATPases). Other unique features include the metal-binding domain in P_{IB}-type ATPases (MBD, green) and a characteristic A-domain/M1 linker in P_{II}-type ATPases (yellow).

Supplementary Fig. 2.

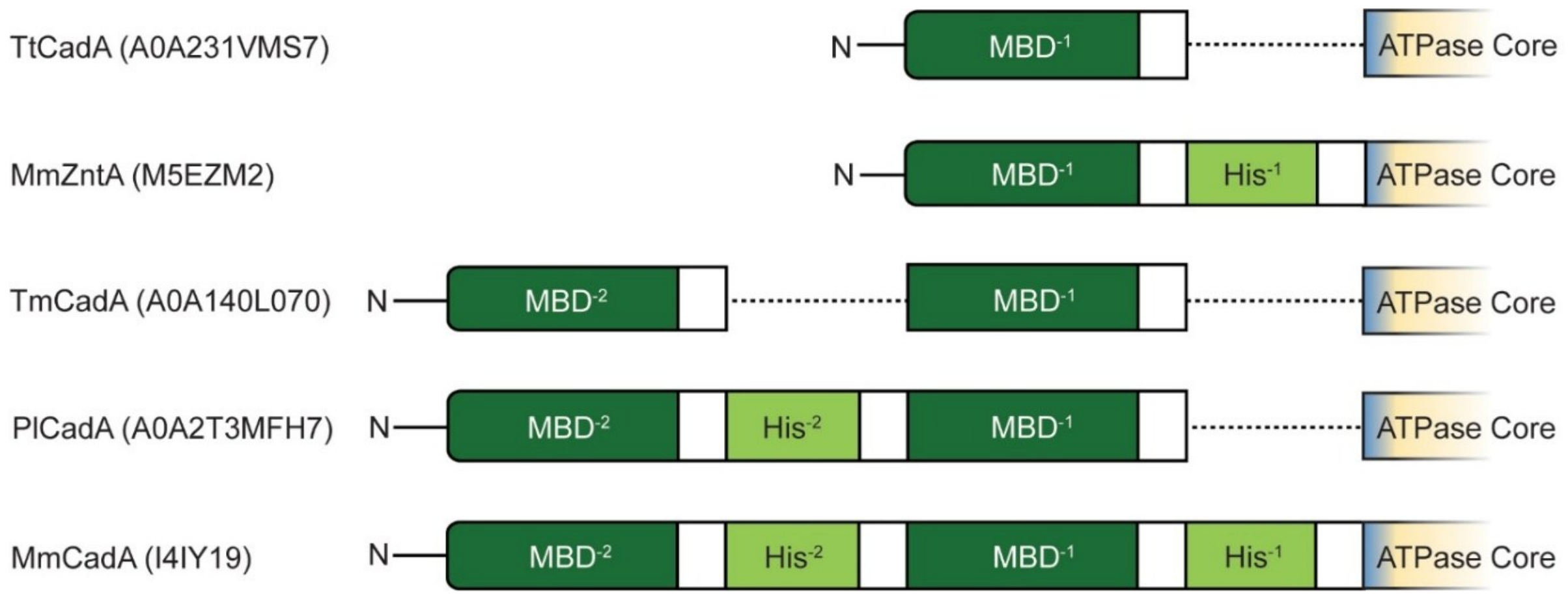

Supplementary Figure 2. N-terminal features of the five selected P_{IB-2}-type ATPases. The N-terminal regions are shown for the following genes (from the top): CadA from *Thermoanaerobacterium thermosaccharolyticum* (UniProt A0A231VMS7), ZntA from *Mesorhizobium metallidurans* (M5EZM2), CadA from *Thermotaela metallivorans* (A0A140L070), CadA from *Photobacterium leiognathi* (A0A2T3MFH7), and CadA from *Mesorhizobium metallidurans* (I4IY19). The metal-binding domains (MBD) are highlighted in dark green, and the histidine-rich regions (His^{-1} and His^{-2}) in light green.

Supplementary Fig. 3.

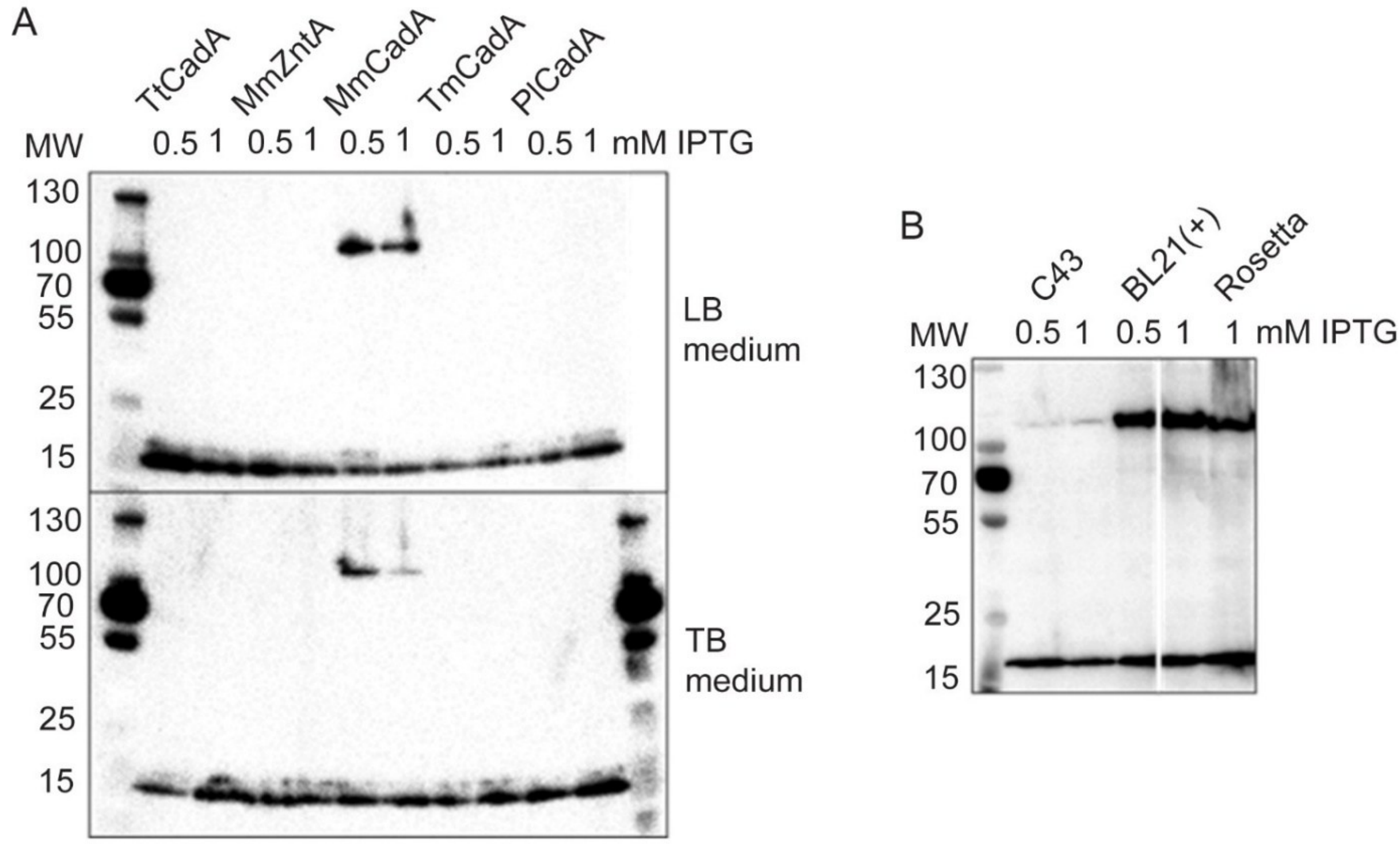

Supplementary Figure 3. Evaluation of production yields for the five selected P_{IB-2}-type ATPases by western blot. a, Small-scale cultures of *E. coli* BL21(+) grown in LB and TB media expressing P_{IB-2}-ATPases from *Thermoanaerobacterium thermosaccharolyticum* (TtCadA) induced with 0.5 mM (lane 2) or 1 mM (lane 3) IPTG, *Mesorhizobium metallidurans* ZntA (MmZntA, lanes 4-5) and CadA (MmCadA, lanes 6-7), *Thermotaela metallivorans* (TmCadA, lanes 8-9), and *Photobacterium leiognathi* (PlCadA, lanes 10-11). Among these, only MmCadA (band at approximately 110 kDa) displays robust protein expression under the tested conditions. **b,** Overproduction test of MmCadA in *E. coli* C43 induced with 0.5 mM (lane 2) or 1 mM (lane 3) IPTG, in *E. coli* BL21(+) (lanes 4-5) and in *E. coli* Rosetta (lane 6). The highest expression levels were obtained with strain BL21(+) cells grown in LB medium.

Supplementary Fig. 4.

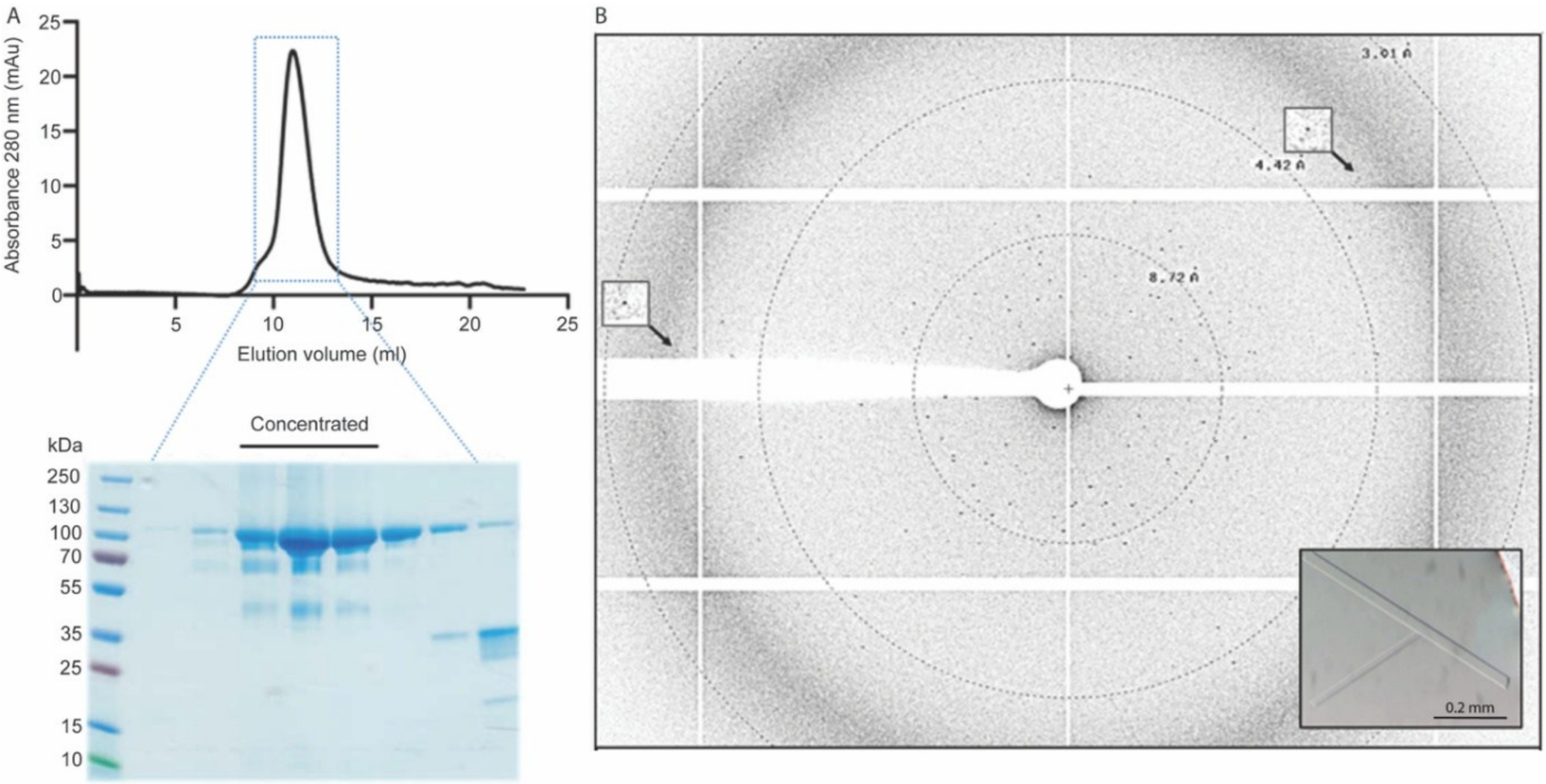

Supplementary Figure 4. Purification and diffraction quality of MmCadA. a, Size-exclusion chromatography profile of purified MmCadA using a Superose 6 Increase 10/300 column (Cytiva). Fractions corresponding to the region highlighted by the blue rectangle were analyzed by SDS-PAGE. The peak fractions were pooled, concentrated, and subsequently used for crystallization. **b,** The dataset from the highest-quality crystal (large inset in bottom right corner) diffracts to 3.6 Å. Shown are the diffraction pattern and a close-view of the highlighted spots at around 3.6 Å resolution (smaller insets, connected to black arrows). See also Supplementary Table 1.

Supplementary Fig. 5.

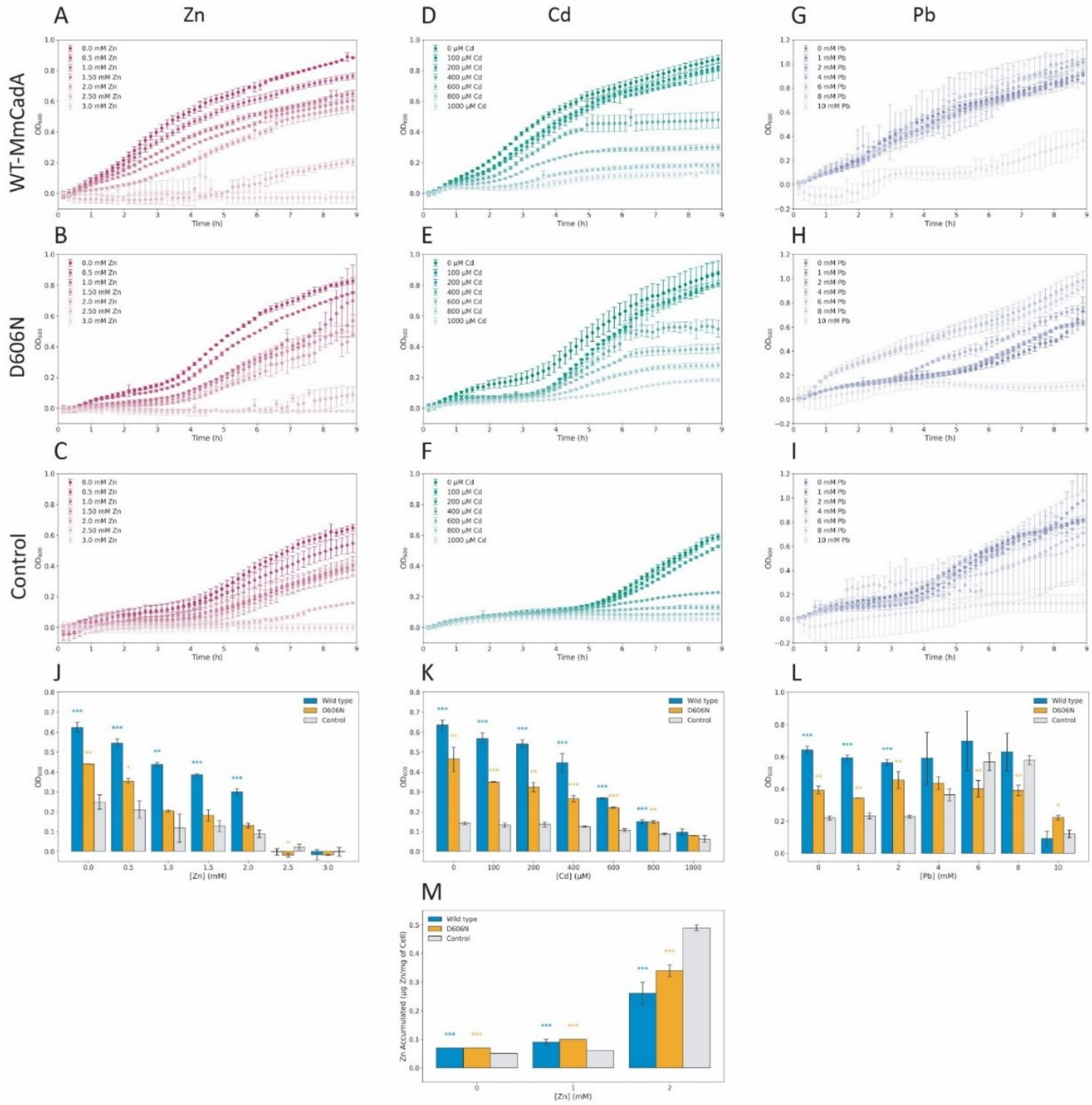

Supplementary Figure 5. ***In vivo* metal challenge assays for MmCadA**. **a-i,** Growth curves from metal susceptibility assays with *E. coli* cells expressing wild-type MmCadA, the inactive mutant D606N, or a control pET vector encoding no protein, cultured in LB media supplemented with Zn^{2+} (**a-c**), Cd^{2+} (**d-f**), or Pb^{2+} (**g-i**). Cells were grown for 9 hours at 37 °C in the presence of 0.5 mM IPTG. **j-l,** Bar plots summarizing OD_{600} values measured at 5 hours of metal exposure for Zn^{2+} (**j**), Cd^{2+} (**k**), and Pb^{2+} (**l**). **m,** Intracellular zinc quantification by ICP-MS in cells expressing the same constructs following exposure to 0-2 mM Zn^{2+} for 20 h at 16 °C in the presence of 0.5 mM IPTG. Cell mass was recorded prior to digestion with nitric acid to normalize intracellular zinc accumulation. Notably, in all experiments, precultures used for inoculation were normalized to identical OD_{600} (2.0) to ensure comparable initial cell densities across all conditions. Statistical significance was calculated by unpaired Student's *t*-test (*** for $p < 0.001$, ** for $p < 0.01$, and * for $p < 0.05$).

Supplementary Fig. 6.

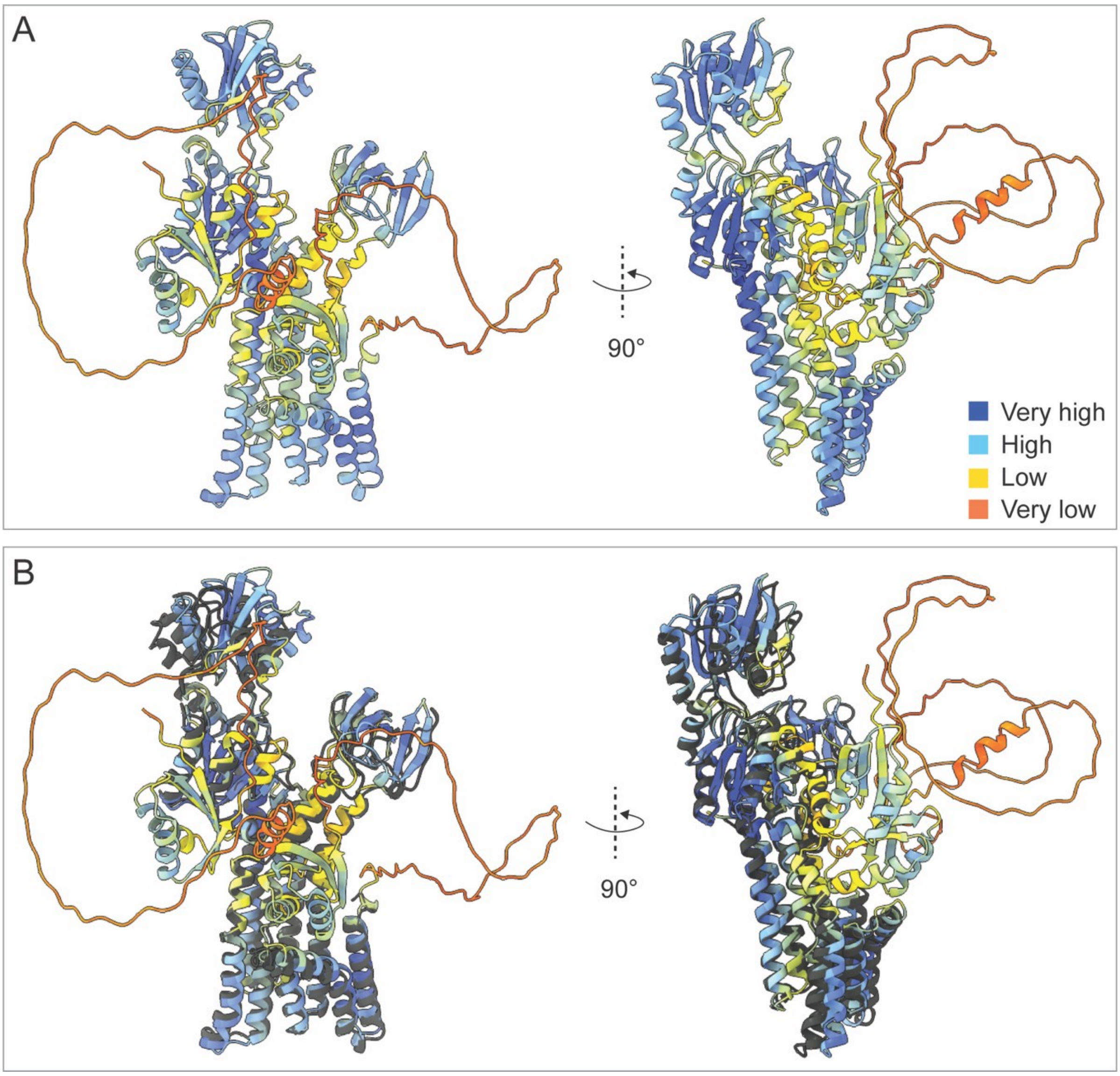

Supplementary Figure 6. Overview of the AlphaFold model. The AlphaFold model based on UniProt ID I4IY19 used for molecular replacement and MD simulations, showing the model on its own (A, colored by AlphaFold confidence score) and superposed with the final MmCadA crystal structure (B, crystal structure in dark gray, RMSD 2.3).

Supplementary Fig. 7.

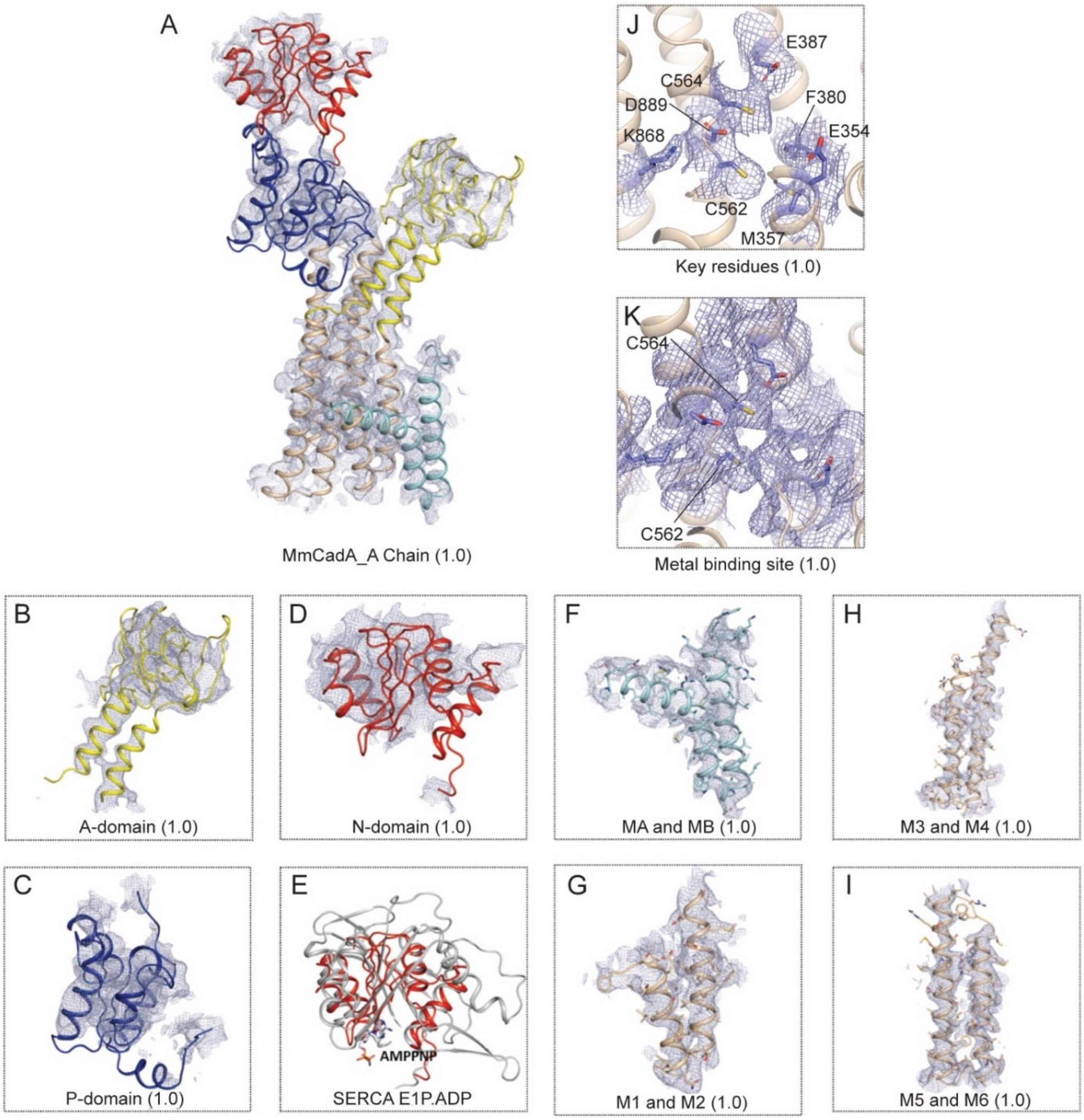

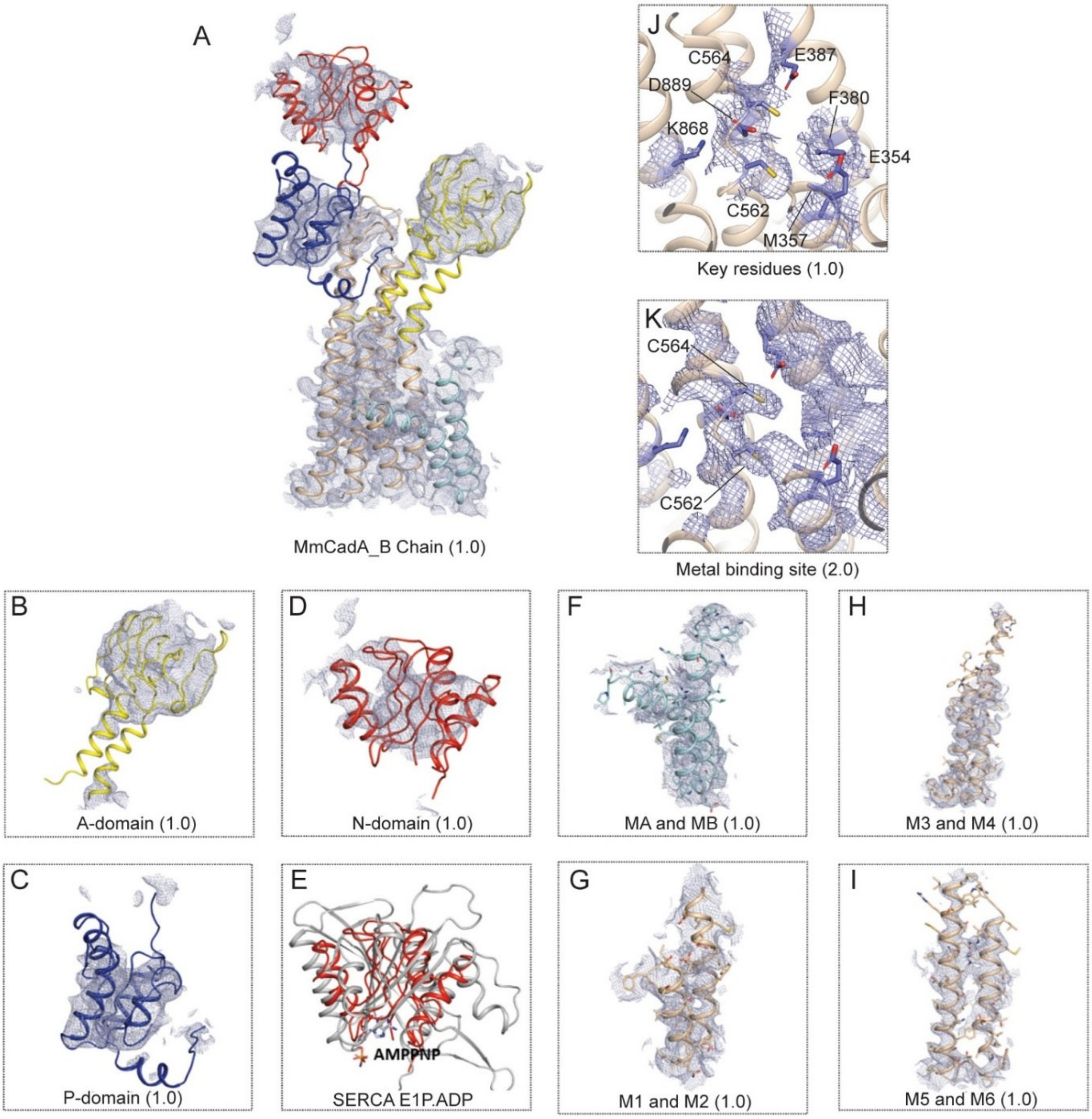

Supplementary Figure 7. Quality of the final electron density maps. All densities represent the final $2F_o$-F_c maps (blue mesh), contoured at σ=1.0. Monomer A is displayed on the top page, and monomer B on the page below. **a,** Overall structure of MmCadA. **b-d,** The three cytosolic (A-, P-, and N-) domains. **e,** Close-view of the N-domain (as in panel d) of SERCA (PDB-ID: 3BA6[47]), determined in the presence of AMPPNP, analogous to MmCadA. **f-i,** The transmembrane helices. **j-k,** Close-views of the entry region, showing density within 2.5 Å of key residues (j) or within 8 Å of those key residues, using a view from the cytoplasm of the metal binding site (k, contoured at σ=2.0 for the B chain).

Supplementary Fig. 8.

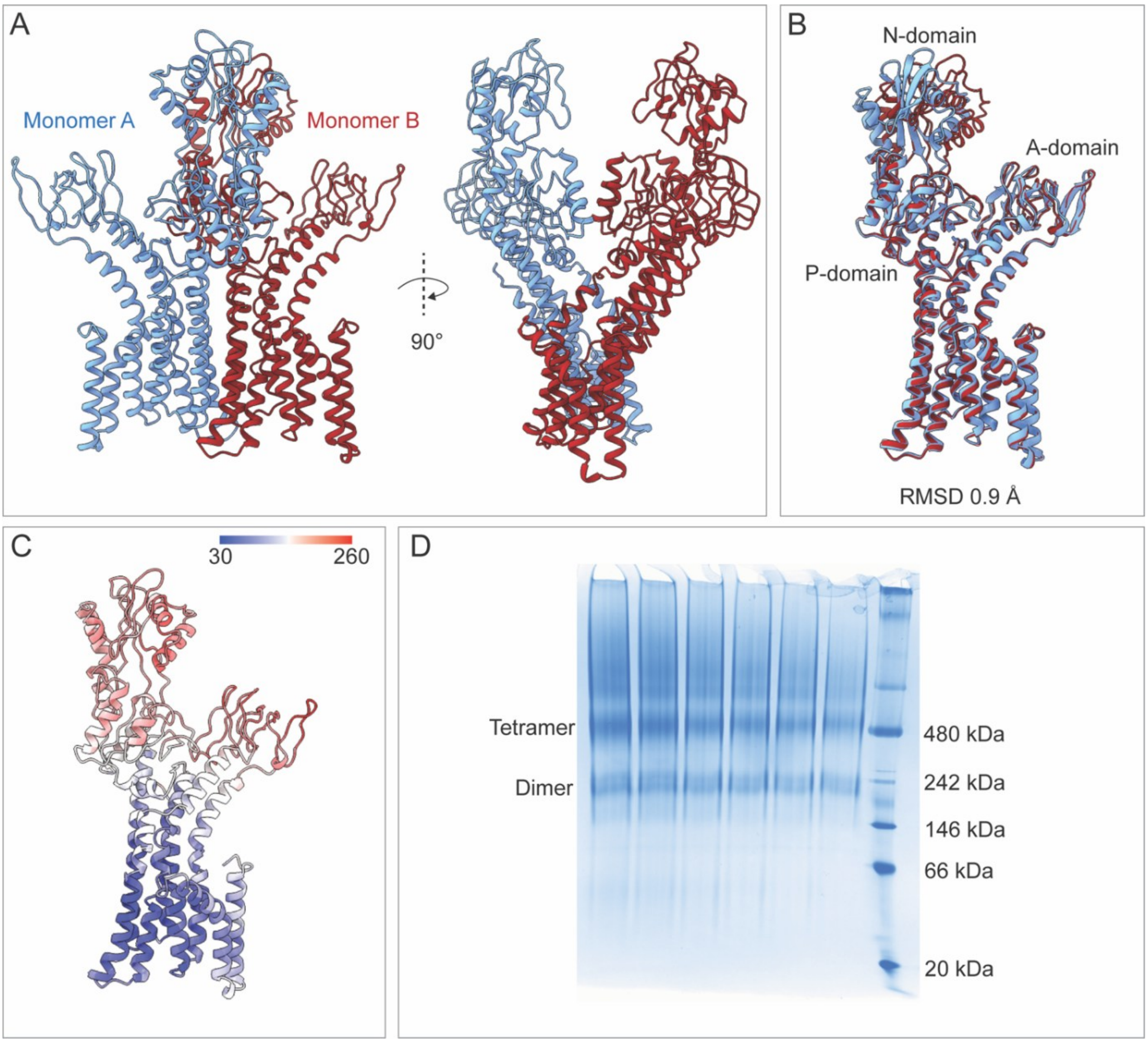

Supplementary Figure 8. Dimer arrangement of MmCadA. a, Monomers A and B are colored blue and red, respectively. **b,** Structural alignment of monomers A and B, with the corresponding root-mean-square deviation (RMSD) of atomic positions indicated. **c,** Structure of MmCadA colored according to b-factor, by ChimeraX[70]. **d,** Native-PAGE gel of MmCadA.

Supplementary Fig. 9.

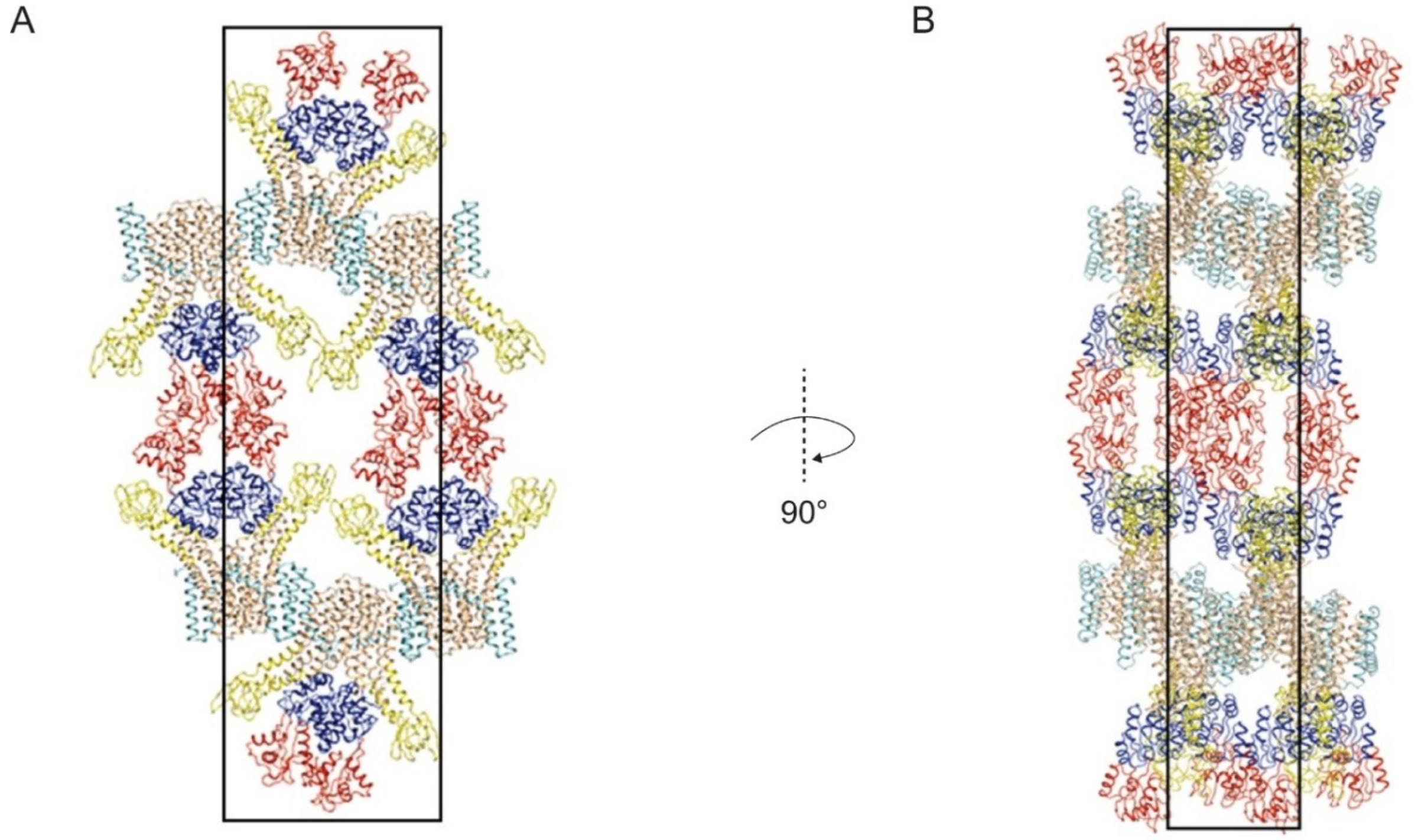

Supplementary Figure 9. Crystal packing of MmCadA. a, The soluble A-, P- and N-domains are colored yellow, blue, and red, respectively. The transmembrane helices are shown in cyan (MA and MB) and wheat (M1-M6). The crystal adopts the $P2_12_12$ space group. Black rectangles indicate the unit cell. **b,** View rotated 90° relative to panel a.

Supplementary Fig. 10.

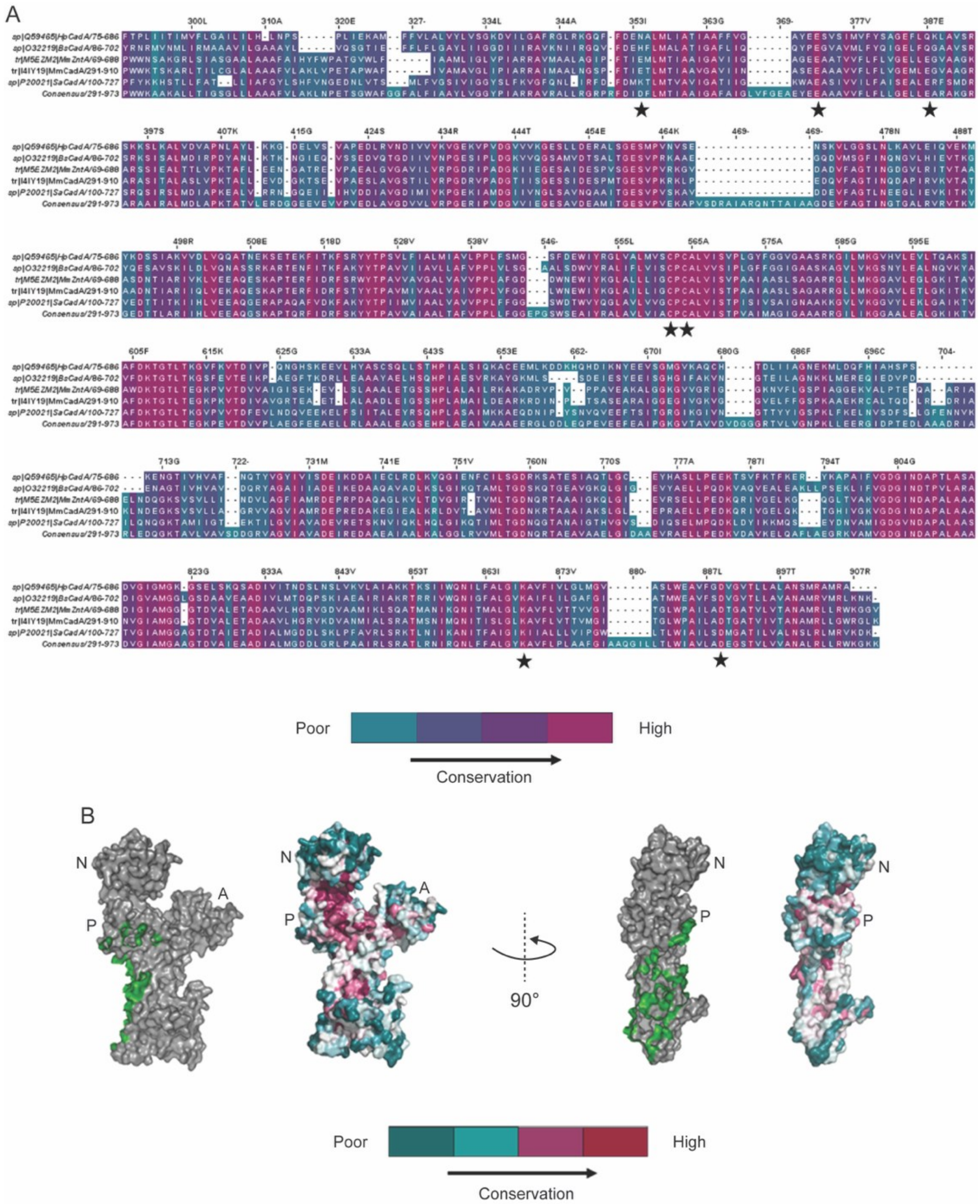

Supplementary Figure. 10. Sequence and surface conservation of P_{IB-2}-type ATPases. a, Multiple sequence alignment of selected P_{IB-2}-type ATPases. Conservation patterns were derived from 26 P_{IB-2}-type ATPase sequences with less than 95 % sequence identity. Five representative P_{IB-2}-type ATPases are shown: HpCadA (UniProt Q59465), BsCadA (O32219), MmZntA (M5EZM2), MmCadA (I4IY19), and SaCadA (P20021). Alignment was performed using Clustal Ω[84]. Black stars under the alignment indicate residues highlighted in this study. **b,** Conservation of the monomer-monomer interface surface, analyzed using the Consurf server[85]. Light green highlights the interface, with dark red and teal representing highly and poorly conserved residues, respectively. The soluble A-, N- and P-domains have been labeled.

Supplementary Fig. 11.

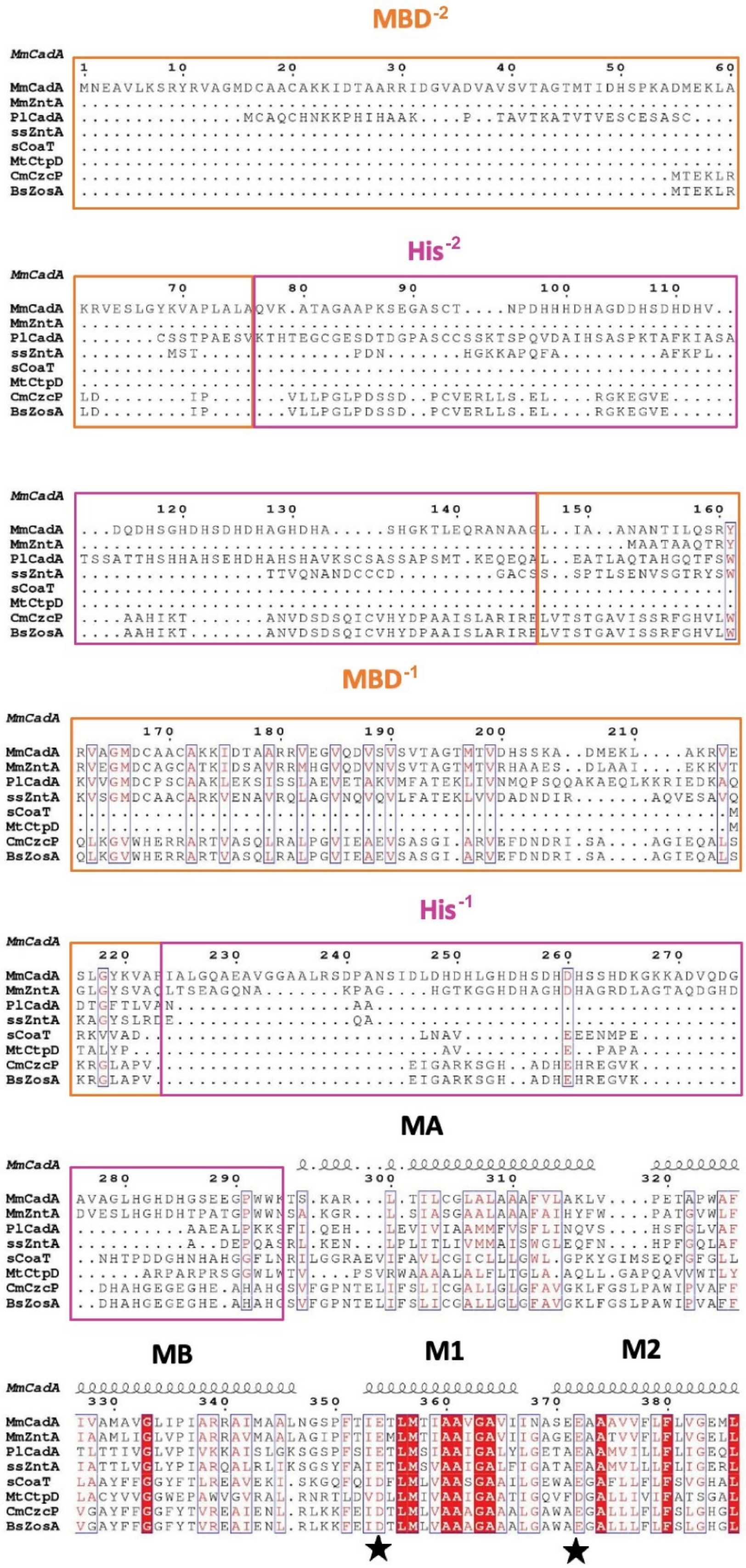

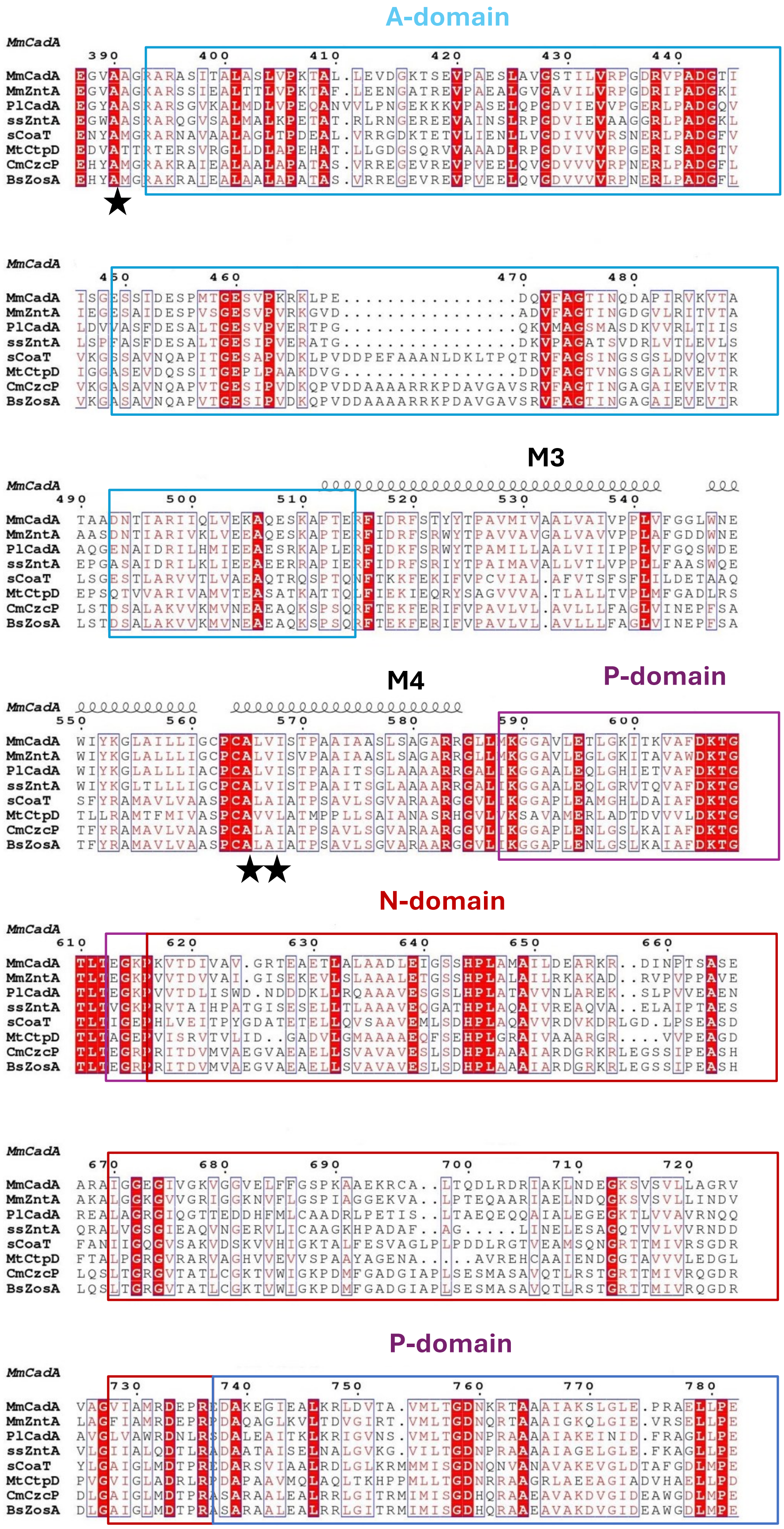
A-domain
M3
M4
P-domain
N-domain
P-domain
MmCadA
MmZntA
PlCadA
ssZntA
sCoaT
MtCtpD
CmCzcP
BsZosA

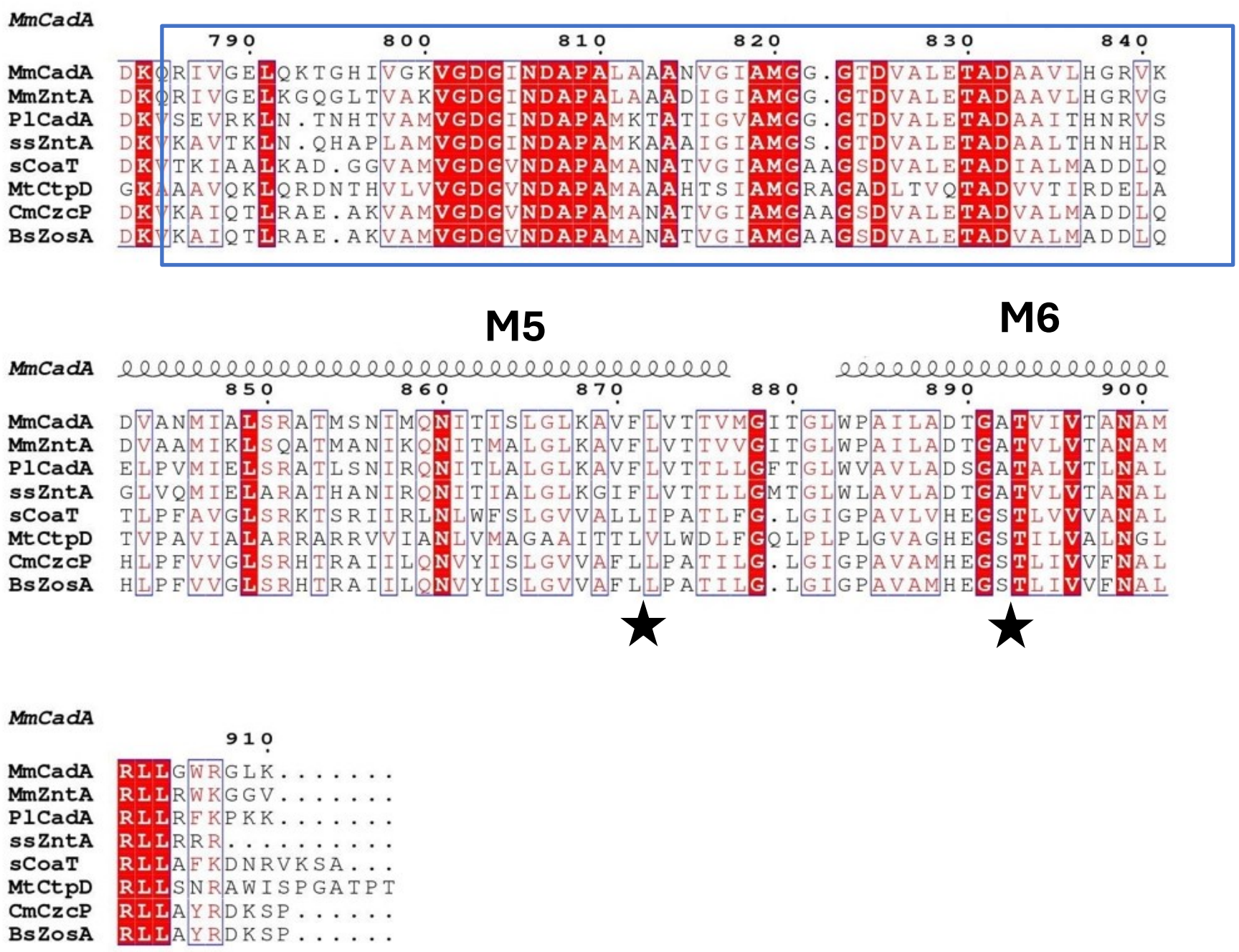

Supplementary Figure 11. Sequence alignment of selected P_{IB-2} and P_{IB-4}-type ATPases. The alignment was performed using Clustal Ω[84] and visualized with ESPript 3.0 (Ref [86]). The P_{IB-2}-type ATPases include MmCadA (UniProt I4IY19), MmZntA (M5EZM2), PlCadA (A0A2T3MFH7), and SsZntA (Q3YW59). The P_{IB-4}-type ATPases include sCoaT (A3T2G5), MtCtpD (P9WPT2), CmCzcP (Q1LAJ7), and BsZosA (O31688). Black stars beneath the alignment indicate residues discussed in this study.

Supplementary Fig. 12.

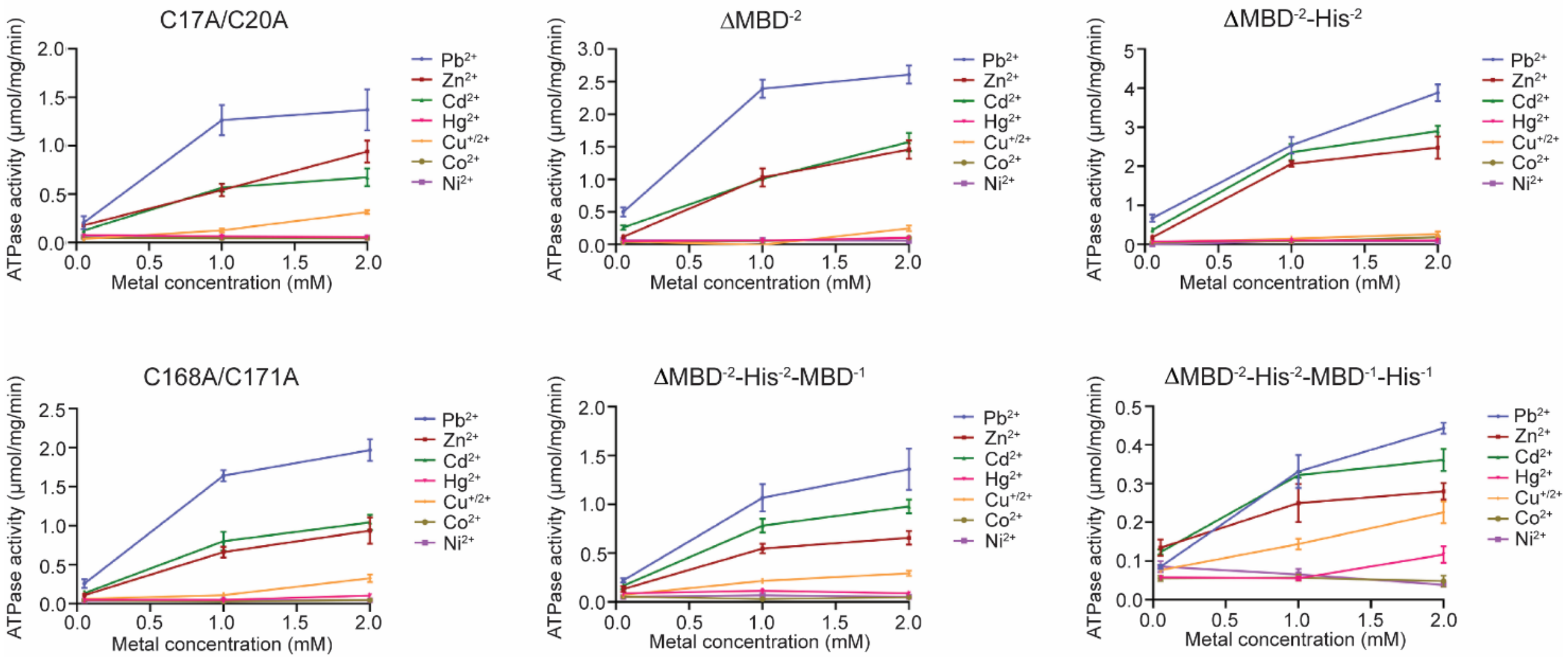

Supplementary Figure. 12. Survey of N-terminal modifications of MmCadA as a function of metal ion concentration. The N-terminal variants (C17A/C20A, ΔMBD^{-2}, ΔMBD^{-2}-His^{-2}, C168A/C171A, ΔMBD^{-2}-His^{-2}-MBD^{-1} and ΔMBD^{-2}-His^{-2}-MBD^{-1}-His^{-1}) were produced and purified using the same protocol as wild-type MmCadA. Functional assays were conducted in a lipid-detergent mixture at 37 °C, in the presence of 20 mM L-cysteine, 5 mM $MgCl_2$, and 3 mM ATP. Data are shown as mean ± SD. Error bars represent the standard deviation of three independent technical measurements performed using material from a single protein purification. Note the varying scales of the y-axes, for clearer representation of each plot.

Supplementary Fig. 13.

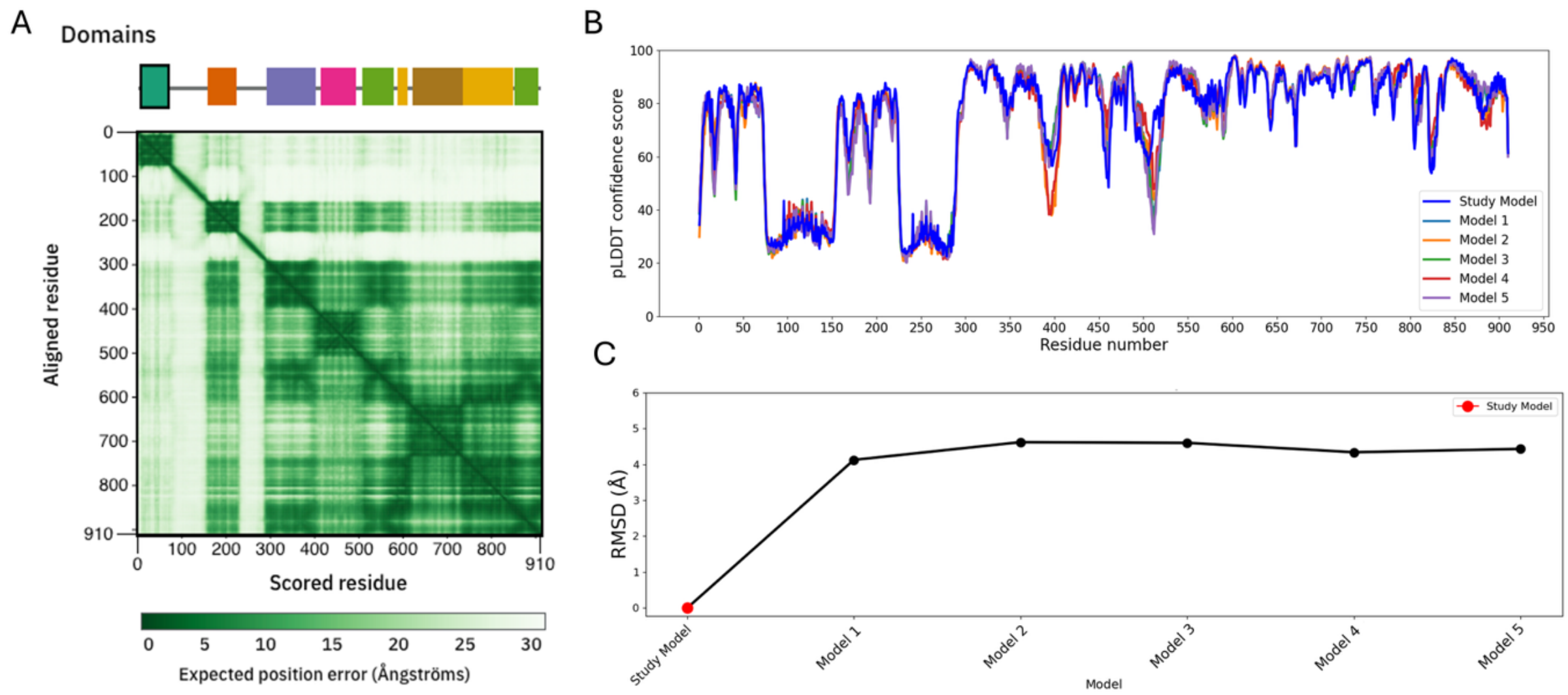

Supplementary Figure 13. a, Predicted Aligned Error (PAE) of the AlphaFold model of wild-type MmCadA retrieved from UniProt (ID: I4IY19), representing the reference structure used in this study (study model). **b,** Per-residue pLDDT confidence scores comparing the study model with five AlphaFold-predicted models generated from independent seeds (best ranked model from each seed). **c,** Root-mean-square deviation (RMSD) of full-length MmCadA models relative to the study model, which is used as the reference (0 Å). RMSD values are shown for the top-ranked models obtained from five AlphaFold predictions (different random seeds), providing a measure of structural variation across generated models.

Supplementary Fig. 14.

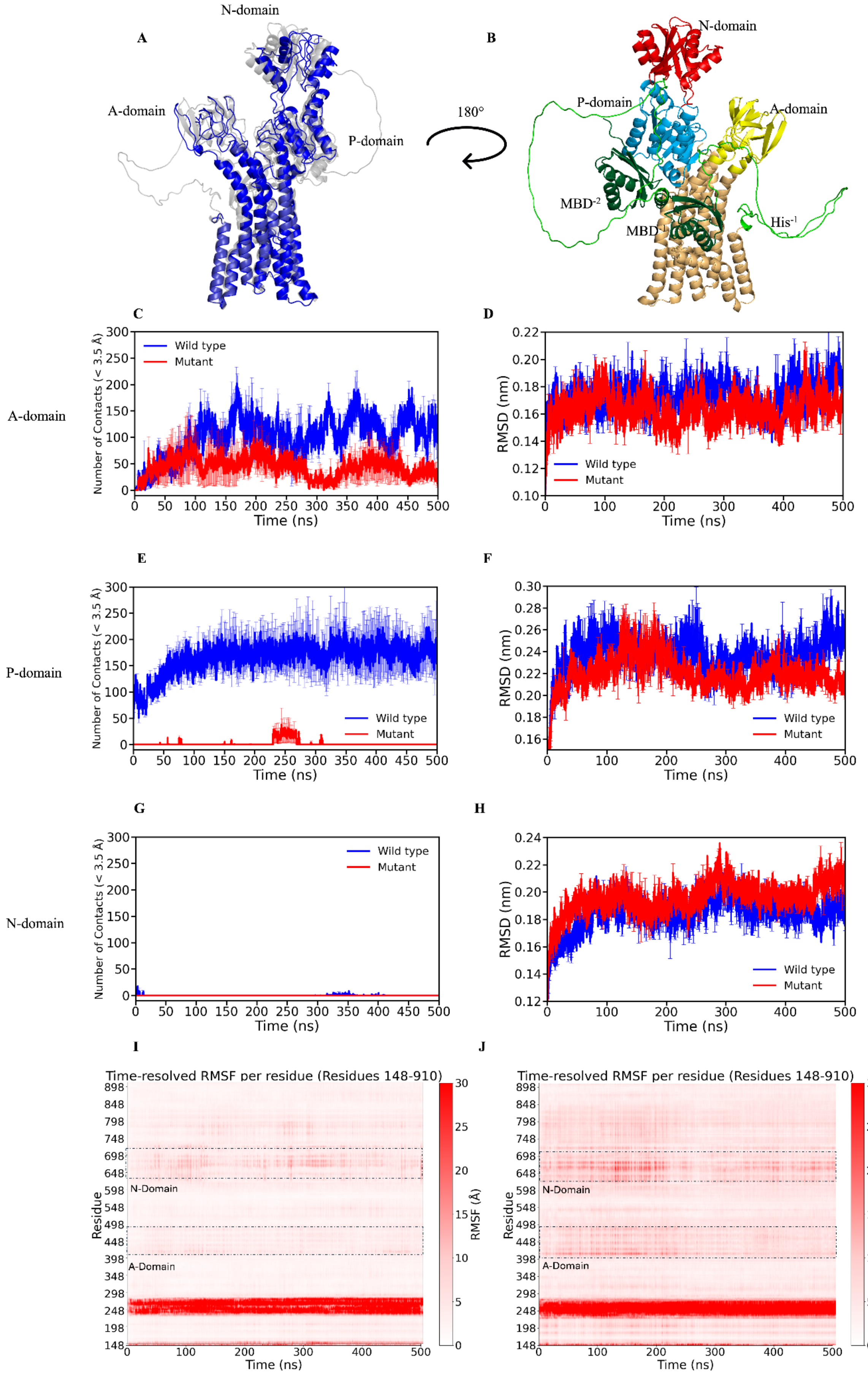

Supplementary Figure 14. a, Structural comparison of the here determined structure of of MmCadA (blue) with the AlphaFold model (grey). **b,** AlphaFold model of MmCadA. **c-j,** Comparison of molecular dynamics simulation results for wild-type MmCadA and the ΔMBD^{-2}-His^{-2} mutant across three simulation replicas. For the wild-type, the N-terminal region corresponds to residues 1-299, while in the ΔMBD^{-2}-His^{-2} mutant it spans residues 148-299. **c,** Average number of contacts between the A-domain and the N-terminal region. **d,** Average RMSD values for the A-domain. **e,** Average number of contacts between the N-domain and the N-terminal region. **f,** Average RMSD values for the N-domain. **g,** Average number of contacts between the P-domain and the N-terminal region. **h,** Average RMSD values for the P-domain. All contact numbers were calculated based on atom pairs. Error bars shown in panels c-h represent the standard error of the mean (SEM). **i,** Time resolved RMSF comparison between the wild-type (**i**) and ΔMBD^{-2}-His^{-2} mutant (**j**) from the combined trajectories of three replicas.

Supplementary Fig. 15.

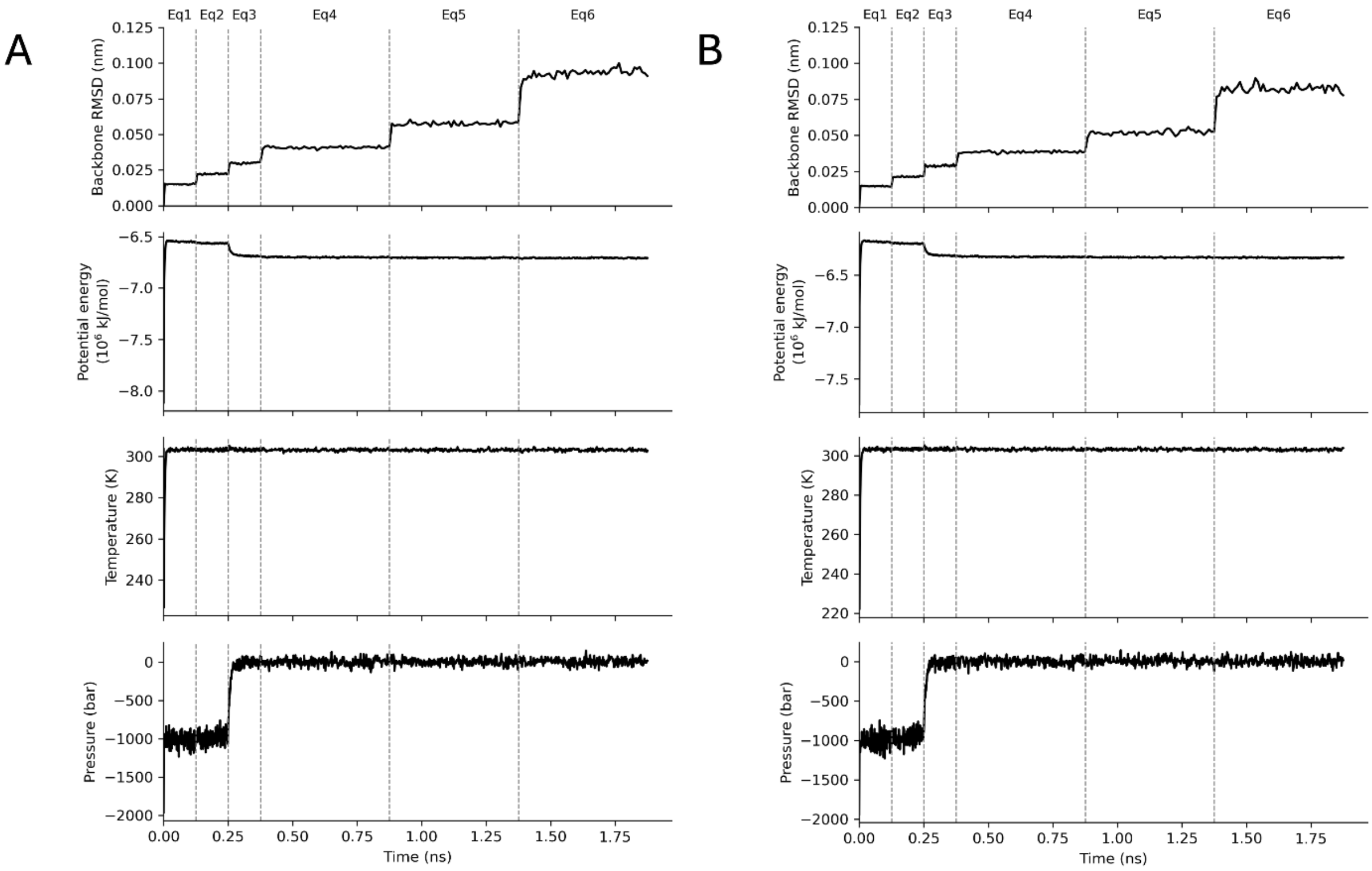

Supplementary Figure 15. Equilibration of the wild-type and ΔMBD/His^{-2} systems. Backbone RMSD, potential energy, temperature, and pressure are shown across the six equilibration steps for the wild-type system (panel **a**) and the ΔMBD/His^{-2} system (panel **b**). Vertical dashed lines indicate transitions between equilibration steps Eq1-Eq6. The stepwise shifts in backbone RMSD coincide with the progressive reduction of positional restraints, whereas fluctuations within each equilibration step remain small. During the final equilibration step, Eq6, the backbone RMSD remains stable, supporting that both systems are sufficiently equilibrated for the production simulations. Temperature, pressure, and potential energy remain stable following the initial system relaxation.

Supplementary Table 1.

	MmCadA-E1
**Data collection**	
Temperature (K)	100
Wavelength (Å)	1.0
Space group	$P2_12_12$
Cell dimensions	
a, *b*, *c* (Å)	100.2 338.0 56.4
α, β, γ (°)	90, 90, 90
Resolution (Å)	50-3.58 (3.80-3.58)*
R_{merge}	33.6 (193.4)
$I / \sigma I$	5.7 (1.0)
Completeness (%)	99.8 (99.2)
Redundancy	7.87 (6.85)
CC(1/2)	99.5(60.8)
Wilson B-factor ($Å^2$)	105.5
**Refinement**	
Resolution (Å)	50-3.58 (3.74-3.58)
No. reflections	23484 (2860)
R_{work} / R_{free}	27.7/31.5 (38.5/50.3)
R.m.s. deviations	
Bond lengths (Å)	0.003
Bond angles (°)	0.75
**Model statistics**	
Residues in favored region (%)	76.7
Residues in allowed region (%)	19.4
Rotamer outliers (%)	3.9
Clash score	30.3
Average B-factor ($Å^2$)	146.5

Supplementary Table 1. X-ray data collection and refinement statistics for MmCadA.

Supplementary Table 2.

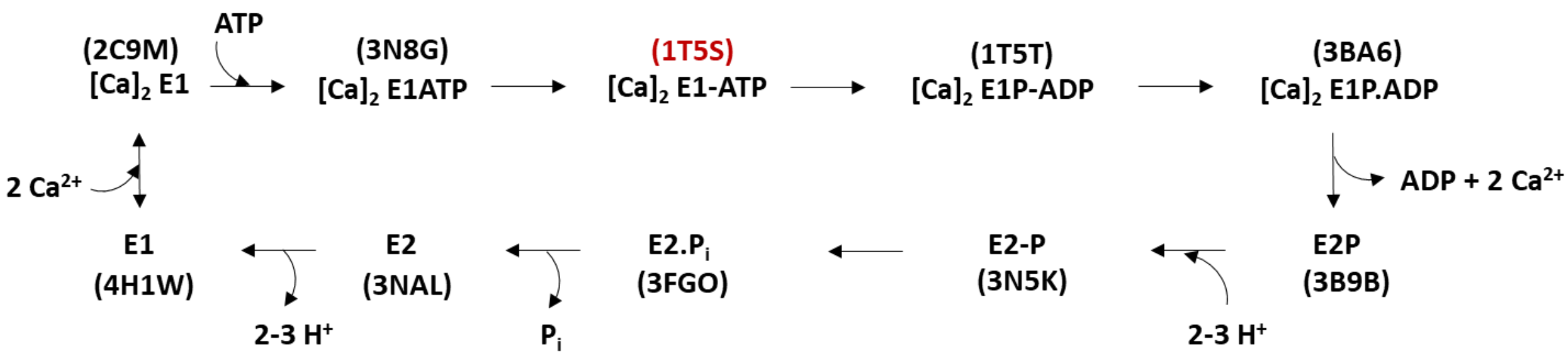

SERCA State	SERCA PDB	MmCadA (full length)	MmCadA (A+P+N)	MmCadA (P+N)	MmCadA (A+P)
E1	4H1W	5.8	5.2	3.8	6.1
$[Ca]_2$ E1	2C9M	9.6	9.3	9.6	3.5
$[Ca]_2$ E1ATP	3N8G	3.9	4.6	2.5	8.1
$[Ca]_2$ E1-ATP	1T5S	4.0	4.6	2.4	7.6
$[Ca]_2$ E1P-ADP	1T5T	4.2	5.4	2.5	7.7
$[Ca]_2$ E1P.ADP	3BA6	4.3	4.8	2.5	7.7
E2P	3B9B	14.2	16.6	7.7	8.9
E2-P	3N5K	14.1	14.5	5.8	9.5
E2.P_i	3FGO	13.9	14.7	5.6	9.5
E2	3NAL	11.7	12.2	3.6	8.9

P_{IB} State	P_{IB} PDB	MmCadA (full length)	MmCadA (A+P+N)	MmCadA (P+N)	MmCadA (A+P)
LpCopA (E2P)	4BBJ	11.1	13.3	7.5	8.6
LpCopA (E2-P)	3RFU	12.8	14.2	6.2	8.9
SsZntA (E2P)	4UMV	10.8	13.5	6.3	9.0
SsZntA (E2-P)	4UMW	12.1	13.7	5.5	8.7
AfCopA (E1)	7R0I	2.3	2.1	1.7	1.9

Supplementary Table 2. Structural comparison with RMSDs of the obtained MmCadA structure compared to available structures of SERCA and P_{IB}-ATPases. Domain numbering follows the references indicated: SERCA A 1-40 + 128-241, N 357-600, P 330-356 + 601-738 (ref [87]), LpCopA A 206-334, N 431-553, P 403-430 + 554-663 (ref [23]), SsZntA A 222-344, N 444-557, P 415-443 + 558-664, (ref [20]), AfCopA A 211-327, N 433-548, P 403-432 + 550-653 (ref [39]). The red numbers indicate the structures that are most similar to the experimentally determined MmCadA structure. For simplicity only comparisons with the A chain are shown, results were similar for the B chain.

Supplementary Table 3.

Variant	Modifications	Features
MmCadA	None	Wild-type
C17A/C20A	C17A,C20A	Metal-binding site of distal MBD^{-2} is inactivated
C168A/C171A	C168A,C171A	Metal-binding site of MBD^{-1} is inactivated
ΔMBD^{-2}	Deletion 1-71	MBD^{-2} is removed
ΔMBD^{-2}-His^{-2}	Deletion 1-147	MBD^{-2} and the first histidine-rich region are removed
ΔMBD^{-2}-His^{-2}-MBD^{-1}	Deletion 1-223	The MBDs are removed
ΔMBD^{-2}-His^{-2}-MBD^{-1}-His^{-1}	Deletion 1-299	The MBDs and the histidine-rich regions are removed

Supplementary Table 3. N-terminal variants of MmCadA employed in functional and structural experiments.

Supplementary Table 4.

Variant	Sequence (5'- 3')
C17A/C20A fwd	CGTGTTGCGGGTATGGATGCCGCGGCGGCCGCGAAGAAAATCGACAC CGCGGCGCGTCGTATTGAT
C17A/C20A rev	ATCAATACGACGCGCCGCGGTGTCGATTTTCTTCGCGGCCGCCGCGGC ATCCATACCCGCAACACG
ΔMBD^{-2} fwd	CTGGCGCTGGCGCAGGTTAAAGCG
ΔMBD^{-2} rev	CATCTGGTACCCGGGTCCCTGAAA
ΔMBD^{-2}-His^{-2} fwd	ACCAGATGCTGATTGCGGCGAACGCG
ΔMBD^{-2}-His^{-2} rev	CATCTGGTACCCGGGTCCCTGAAA
C168A/C171A fwd	CGTGTTGCTGGCATGGACGCCGCTGCTGCCGCGAAGAAAATCGATACC GCGGCGCGTCGTGTGGAG
C168A/C171A rev	CTCCACACGACGCGCCGCGGTATCGATTTTCTTCGCGGCAGCAGCGGC GTCCATGCCAGCAACACG
ΔMBD^{-2}-His^{-2}-MBD^{-1} fwd	ATTGCGCTGGGTCAGGCGGAAGCG
ΔMBD^{-2}-His^{-2}-MBD^{-1} rev	CATCTGGTACCCGGGTCCCTGAAA
ΔMBD^{-2}-His^{-2}-MBD^{-1}-His^{-1} fwd	GGTAGCGAGGAAGGTCCGTGGTGG
ΔMBD^{-2}-His^{-2}-MBD^{-1}-His^{-1} rev	CATCTGGTACCCGGGTCCCTGAAA
A91C fwd	CGAGGGTTGCAGCTGCACCAACC
A91C rev	GCAGCTGCAACCCTCGCTTTTCGG
A489C fwd	AGGTTACCTGCACCGCGGCGGATAAC
A489C rev	CGCGGTGCAGGTAACCTTCACACGAATC
H99C fwd	AACCCGGATCACTGCCATGACCATG
H99C rev	TCATGGCAGTGATCCGGGTTGGTG
S643C fwd	GAAATT GGTAGCTGCCACCCGCTG
S643C rev	GGTGGCAGCTACCAATTTCCAGGTC

Supplementary Table 4. List of the primers used in this work. The primers were purchased from ThermoFisher and Eurofins.

Supplementary Table 5.

Category	Wild-type MmCadA	ΔMBD/His-2 variant
Starting structure	AlphaFold model from UniProt, ID I4IY19	Generated from wild-type AlphaFold model by removing the N-terminal MBD^{-2}/His^{-2} region
Membrane builder	CHARMM-GUI bilayer builder	CHARMM-GUI bilayer builder
Lipid composition	915 POPC molecules	915 POPC molecules
Water model	TIP3P	TIP3P
Number of water molecules	154,765	146,296
Ions/salt	438 K^+, 426 Cl^-; ~0.15 M KCl plus 12 K^+ for neutralization	408 K^+, 403 Cl^-; ~0.15 M KCl plus 5 K^+ for neutralization
Initial box dimensions	18.01 × 18.01 × 19.57 nm	18.01 × 18.01 × 18.69 nm
MD software	GROMACS 2023.3	GROMACS 2023.3
Energy minimization	Steepest descent, maximum force <1000 kJ mol-1 nm-1	Same
Equilibration protocol	Six-step equilibration with progressively reduced atom position restraints. Steps 1-2 were 125 ps each with a 1 fs timestep at 303.15 K; step 3 was 125 ps with a 1 fs timestep at 303.15 K and 1 bar; steps 4-6 were 500 ps each with a 2 fs timestep at 303.15 K and 1 bar.	Same
Production simulations	Three-replica exchange MD simulations, 500 ns per replica	Same
Replica temperatures	303.15-303.25 K	303.15-303.25 K
Thermostat	V-rescale thermostat	V-rescale thermostat
Barostat	C-rescale barostat	C-rescale barostat
Pressure	1 bar	1 bar
Electrostatics	Smooth particle mesh Ewald, 12 Å cutoff	Same
Bond constraints	LINCS algorithm for bonds involving hydrogen atoms	Same
Trajectory analysis	GROMACS 2023.3	Same
Visualization/plotting	PyMOL and Python	Same

Supplementary Table 5. Summary of molecular dynamics simulation setup.

Supplementary References.

84. Madeira, F. et al. The EMBL-EBI search and sequence analysis tools APIs in 2019. *Nucleic Acids Res* **47**, W636–W641 (2019).
85. Ashkenazy, H., Erez, E., Martz, E., Pupko, T. & Ben-Tal, N. ConSurf 2010: calculating evolutionary conservation in sequence and structure of proteins and nucleic acids. *Nucleic Acids Res* **38**, W529–33 (2010).
86. Robert, X. & Gouet, P. Deciphering key features in protein structures with the new ENDscript server. *Nucleic Acids Res* **42**, W320–4 (2014).
87. Aguayo-Ortiz, R. & Espinoza-Fonseca, L.M. Linking Biochemical and Structural States of SERCA: Achievements, Challenges, and New Opportunities. *Int J Mol Sci* **21**(2020).
