# Supplementary material for: Structural and functional insights into a Zn2+-transporting P-type ATPase
Source: Nat Commun. 2026 Sep 16;17:10886. doi: 10.1038/s41467-026-77558-1 (PMC13677743; doi:10.1038/s41467-026-77558-1)
Supplement: Supplementary file 2 [file 41467_2026_77558_MOESM2_ESM.pdf]

nature portfolio

Corresponding author(s): Pontus Gourdon

Last updated by author(s): Oct 10, 2025

Reporting Summary

Nature Portfolio wishes to improve the reproducibility of the work that we publish. This form provides structure for consistency and transparency in reporting. For further information on Nature Portfolio policies, see our Editorial Policies and the Editorial Policy Checklist.

Statistics

For all statistical analyses, confirm that the following items are present in the figure legend, table legend, main text, or Methods section.

n/a	Confirmed	
☐	☒	The exact sample size (*n*) for each experimental group/condition, given as a discrete number and unit of measurement
☐	☒	A statement on whether measurements were taken from distinct samples or whether the same sample was measured repeatedly
☐	☒	The statistical test(s) used AND whether they are one- or two-sided<br>*Only common tests should be described solely by name; describe more complex techniques in the Methods section.*
☒	☐	A description of all covariates tested
☒	☐	A description of any assumptions or corrections, such as tests of normality and adjustment for multiple comparisons
☐	☒	A full description of the statistical parameters including central tendency (e.g. means) or other basic estimates (e.g. regression coefficient) AND variation (e.g. standard deviation) or associated estimates of uncertainty (e.g. confidence intervals)
☐	☒	For null hypothesis testing, the test statistic (e.g. *F*, *t*, *r*) with confidence intervals, effect sizes, degrees of freedom and *P* value noted<br>*Give P values as exact values whenever suitable.*
☒	☐	For Bayesian analysis, information on the choice of priors and Markov chain Monte Carlo settings
☒	☐	For hierarchical and complex designs, identification of the appropriate level for tests and full reporting of outcomes
☒	☐	Estimates of effect sizes (e.g. Cohen's *d*, Pearson's *r*), indicating how they were calculated

Our web collection on statistics for biologists contains articles on many of the points above.

Software and code

Policy information about availability of computer code

Data collection	Data was collected at the Swiss Light Source (Switzerland) on the X06SA beamline.
Data analysis	Collected data was processed using XDS v20190315, and refined using Phenix refine v1.14-3260 and Coot v0.8.9.1. The MD simulations used CHARMM-GUI v3.8 and GROMACS 2021. Figures were generated using Pymol v2.3, ChimeraX v1.10 and Python Matplotlib 3.10.7.

For manuscripts utilizing custom algorithms or software that are central to the research but not yet described in published literature, software must be made available to editors and reviewers. We strongly encourage code deposition in a community repository (e.g. GitHub). See the Nature Portfolio guidelines for submitting code & software for further information.

Data

Policy information about availability of data

All manuscripts must include a data availability statement. This statement should provide the following information, where applicable:

- Accession codes, unique identifiers, or web links for publicly available datasets
- A description of any restrictions on data availability
- For clinical datasets or third party data, please ensure that the statement adheres to our policy

The data that support this study are available from the corresponding author upon reasonable request. The atomic coordinates have been deposited in the Protein Data Bank (PDB) under accession codes XXXX [https://doi.org/XXXX]. All files required for producing the trajectories of the MD simulations can be found at repository: https://doi.org/10.5281/zenodo.21476325.

Research involving human participants, their data, or biological material

Policy information about studies with human participants or human data. See also policy information about sex, gender (identity/presentation), and sexual orientation and race, ethnicity and racism.

Reporting on sex and gender	Study did not involve human participants.
Reporting on race, ethnicity, or other socially relevant groupings	Study did not involve human participants.
Population characteristics	Study did not involve human participants.
Recruitment	Study did not involve human participants.
Ethics oversight	Study did not involve human participants.

Note that full information on the approval of the study protocol must also be provided in the manuscript.

Field-specific reporting

Please select the one below that is the best fit for your research. If you are not sure, read the appropriate sections before making your selection.

[x] Life sciences [ ] Behavioural & social sciences [ ] Ecological, evolutionary & environmental sciences

For a reference copy of the document with all sections, see nature.com/documents/nr-reporting-summary-flat.pdf

Life sciences study design

All studies must disclose on these points even when the disclosure is negative.

Sample size	No formal statistical method was used to predetermine sample size. For quantitative biochemical experiments, a minimum of three independent measurements was performed for each condition, which is standard practice for assessing the reproducibility of biochemical assays and estimating experimental variability. Sample sizes were chosen based on established practice in the field and were sufficient to provide consistent and reproducible results. Structural analyses were based on all high-quality data collected and processed according to established crystallographic workflows, rather than on predetermined sample sizes.
Data exclusions	No data were excluded from this study.
Replication	Structural data is derived from averaging of a large number of individual measurements, and so does not require replication. Functional assays used replicates to ensure reproducibility.
Randomization	Samples were not randomly allocated to experimental groups. Randomization was not relevant because the study involved predefined protein constructs and experimental conditions rather than biological subjects. Each sample was assigned to its respective experimental condition based on the study design, and all measurements were performed using standardized protocols.
Blinding	Investigators were not blinded to group allocation during data collection or analysis. Blinding was not considered relevant because the study involved predefined protein samples analyzed using standardized biochemical, biophysical, and structural methods, with data collected and analyzed using objective experimental and computational procedures.

Reporting for specific materials, systems and methods

We require information from authors about some types of materials, experimental systems and methods used in many studies. Here, indicate whether each material, system or method listed is relevant to your study. If you are not sure if a list item applies to your research, read the appropriate section before selecting a response.

Materials & experimental systems

n/a	Involved in the study	
[ ]	[x]	Antibodies
[x]	[ ]	Eukaryotic cell lines
[x]	[ ]	Palaeontology and archaeology
[x]	[ ]	Animals and other organisms
[x]	[ ]	Clinical data
[x]	[ ]	Dual use research of concern
[x]	[ ]	Plants

Methods

n/a	Involved in the study	
[x]	[ ]	ChIP-seq
[x]	[ ]	Flow cytometry
[x]	[ ]	MRI-based neuroimaging

Antibodies

Antibodies used	Strep-Tag II monoclonal antibody (HRP-conjugated; Cat. No. BSM-33166M-HRP).
Validation	*Describe the validation of each primary antibody for the species and application, noting any validation statements on the manufacturer's website, relevant citations, antibody profiles in online databases, or data provided in the manuscript.*

Plants

Seed stocks	The study did not involve plants
Novel plant genotypes	The study did not involve plants
Authentication	The study did not involve plants
