# Supplementary material for: Structural and functional insights into a Zn2+-transporting P-type ATPase
Source: Nat Commun. 2026 Sep 16;17:10886. doi: 10.1038/s41467-026-77558-1 (PMC13677743; doi:10.1038/s41467-026-77558-1)
Supplement: Supplementary file 3 [file 41467_2026_77558_MOESM3_ESM.pdf]

nature portfolio

Peer Review File

Structural and functional insights into a Zn2+-transporting P-type ATPase

Corresponding Author: Dr Pontus Gourdon

This file contains all reviewer reports in order by version, followed by all author rebuttals in order by version.

Version 0:

Reviewer comments:

Reviewer #1

(Remarks to the Author)
The authors present a low-resolution X-ray structure of a metal transporting P1B-ATPAse from Mesorhizobium metallidurans in E1 state. They also conduct activity assays with several truncation variants of the N-terminal region of the protein, which contains two metal-binding domains that are not resolved in the structure. These measurements together with molecular dynamics simulations lead them to the conclusion that the N-terminus plays an autoinhibitory role through the interactions with the A- or N-domain. The structure misses the metal-binding domains and does not show a metal bound in the transmembrane site. This, together with the low resolution of 3.6 A, limits the novelty of the structure. In fact, it seems that the same conclusions could be obtained simply by analyzing the AlphaFold model of the protein, which only differs by 2.3 A RMSD from the experimental structure, and most of the difference is due to a slight turn of the N-domain. Considering the generally high fidelity of AlphaFold models and already known architecture of P1B-ATPases, I don't think an experimental confirmation of the model is of much value by itself in this case.

1. Why is the protein called Zn2+ ATPase, if a much stronger affinity stimulation is observed with Pb2+ (Fig. 1B)?
2. A more detailed comparison of the experimental structure with the AlphaFold model used for molecular replacement is needed. What is the new knowledge gained from the experimental structure compared to the model, keeping in mind the low resolution of the structure and the fact that the general architecture of P1B-ATPases is well known from several previously published X-ray and Cryo-EM structures?
3. The structure shows an ATPase dimer. Does the protein purify as a dimer? SEC-MALS may be informative, even though the detergent may complicate accurate MW determination.
4. The most interesting aspect of the data shown in Fig. 4B is that the deletion of MBD-2-His-2 fragment appears to change metal specificity: unlike the wild type and other mutation and truncation variants, the activity of this construct is stimulated by Cd2+ and Zn2+ much stronger than by Pb2+. How can the autoinhibition model explain this?
5. What antibody was used for the Western blot in Sup. Fig. 3?
6. Sup. Fig. 5: The inset appears to show crystals, rather than diffraction spots. Please check the legend.
7. Fig. 1B does not show a protein structure (p.5). Please check figure numbering.
8. What do the numbers shown in Sup. Table 2 mean? A more informative table title and/or legend is needed.
9. "Despite the shortage of structural information on metal-uptake and -binding in PIB-type ATPases, previous biochemical studies have identified a single high-affinity Zn2+ binding site [60]". Reference 60 is incorrect here.
10. "The metal likely coordinated by the cysteine thiolates of the conserved CPC motif of the M4 segment and two oxygen ligands from an invariant aspartate of M6, corresponding to C562, C564 and D889 in MmCadA (Supplementary Fig. 9a)" Figure reference is wrong.
11. "In this work we present the first metal-bound structure of a PIB-2-type ATPase, complemented by functional analysis." This is a misleading statement. There is indeed a bound lead atom in the structure, but judging from its location, it is of no functional significance.

Reviewer #2

(Remarks to the Author)
Reviewer comments _ 663116_0

Elements of a reviewer report

Key results
Current models posit that bacterial P-type Zn^{2+}-ATPases operate through an alternating-access mechanism in which Zn^{2+} binds the conserved CPC motif from the cytosolic side, followed by ATP-driven phosphorylation events that occlude and subsequently outwardly expose the ion-binding site to enable Zn^{2+} release and prevent cytosolic toxicity. This work advances that framework by providing a coherent set of structural and functional data, most notably the first well-resolved inward-open conformation of a Zn^{2+}-transporting P-type ATPase, thereby experimentally clarifying how Zn^{2+} gains access to the CPC motif (Mesorhizobium metallidurans MmCadA). The integration of biochemical assays and molecular dynamics supports a regulatory model in which N-terminal metal-binding domains act as Zn^{2+}-responsive auto-inhibitory elements, and the identification of an electronegative uptake region together with a plausible histidine-mediated ion-donation pathway introduces regulatory and mechanistic features not captured in prevailing models. Although the manuscript overstates potential translational implications, the mechanistic insights are substantial, internally consistent, and scientifically meaningful.

Validity
The experimental evidence presented, including targeted mutagenesis, detailed transport assays, molecular dynamics simulations, and supporting biophysical measurements, substantially reinforces the proposed mechanistic model and lends strong validity to the authors' conclusions. The structural and biochemical analyses are executed with commendable rigor, and the convergence of multiple, independent methodological approaches greatly enhances the robustness and credibility of the findings. Overall, the mechanistic framework advanced by the authors is both coherent and compelling, and it aligns convincingly with the structural features and functional behaviors documented throughout the study.

Significance
The manuscript presents conclusions with strong potential and substantial impact on the field of P-type ATPases. By identifying and characterizing structural features specific to P1B-2 ATPases, the study makes a meaningful contribution to our understanding of metal-transport pathways and the molecular determinants underlying substrate specificity and functional regulation.

Importantly, the proposal of a new transport mechanism represents a particularly valuable aspect of the work. Introducing a mechanistic model of this kind can significantly advance the field, as it provides novel hypotheses for how these proteins coordinate metal translocation and how structural elements modulate activity. Such mechanistic insights are especially relevant given that P1B-type ATPases are emerging as key molecular targets in the study of pathogenic organisms that rely on similar transporters during infection. By offering a refined structural and functional framework, the manuscript opens opportunities to better understand the role of these proteins in host–pathogen interactions and to explore their potential as targets for therapeutic intervention.
Overall, the findings have broad implications for structural biochemistry, membrane-transport biology, and the study of metal homeostasis, and they establish a strong foundation for future work on P-type ATPase function and evolution.

Data and methodology
The overexpression and purification strategy is broadly appropriate for studying bacterial Zn^{2+}-transporting P-type ATPases. Screening multiple PIB-2 candidates and selecting MmCadA based on expression is sound, and the membrane preparation, C12E8 solubilization, and Strep-based purification are methodologically solid. The mutagenesis strategy is well designed for probing regulatory elements. Expression in E. coli may also alter metalation or folding relative to the native system. Despite these concerns. Please, clarify this item. The strategy is conceptually appropriate and likely sufficient for structural and biochemical interrogation.
The functional characterization strategy is, in principle, appropriate for probing metal-stimulated ATP hydrolysis in PIB-2-type ATPases. The Baginski assay is a valid choice for quantifying phosphate release, and the authors examine a broad panel of metal ions and use a systematic set of N-terminal mutants and truncations, which is conceptually aligned with dissecting regulatory and metal-handling roles.
The metal-stimulated ATPase data indicate that MmCadA responds to Pb^{2+}, Zn^{2+} and Cd^{2+}, broadly matching the substrate profiles previously reported for PIB-type ATPases. However, as ATP hydrolysis can be activated independently of productive ion transport. Indeed, the activity pattern in Fig. 1B suggests that Pb^{2+} yields the highest Vmax, a behavior typical of soft metals that bind strongly to cysteine-rich motifs such as CPC, and thus potentially reflecting high-affinity but non-physiological interactions. The responses to Zn^{2+} and Cd^{2+} also show shallow activation curves that may reflect enzymatic stimulation rather than efficient translocation. We strongly suggest that confirmative determination of the transported ion(s) will require intracellular metal quantification, such as atomic absorption or ICP-MS, following controlled metal challenges in whole cells.
Regarding the protein crystallization, the protocol is generally sound and appropriate for membrane-protein crystallization, but key details are missing. Index, the SEC buffer composition, and final protein concentration in crystallization drops should be specified. Clarification of the rationale for Pb^{2+} incubation would also improve reproducibility and methodological clarification.
The MD workflow is generally appropriate for exploring conformational differences between the wild-type protein and the ΔMBD/His-2 variant. The use of CHARMM-GUI for bilayer construction, POPC membranes of adequate size, TIP3P water, and GROMACS 2021 is technically sound. Replica exchange across closely spaced temperatures improves sampling, albeit modestly. The multi-step equilibration scheme is standard and correctly documented. However, several limitations reduce the mechanistic strength of the conclusions. First, relying on an AlphaFold model, particularly for a large membrane ATPase, introduces structural uncertainty that should be acknowledged or validated (e.g., via available homologous

structures). Second, the 100 ns trajectories, even with three replicas, are relatively short for capturing domain rearrangements relevant to P-type ATPase regulation.
The structural characterization of the PIB-2 ATPase MmCadA combines experimental crystallography with complementary bioinformatic modelling to elucidate its architecture and conformational state. Crystals obtained under conditions favoring an inward-open E1 conformation, high lipid and detergent content, Pb^{2+}, Mg^{2+} and AMPPNP, enabled the determination of the structure at 3.6 Å via molecular replacement using an AlphaFold-predicted model. The resulting structure reveals a conserved P-type ATPase core comprising the A-, P- and N-domains and an eight-helix transmembrane region (MA–M6), whereas the flexible N-terminal MBDs and histidine-rich segments are unresolved, consistent with their intrinsic disorder. The well-defined P- and M-domains support the assignment of an early metal-uptake state, while subtle intra-domain shifts suggest conformational heterogeneity typical of transport intermediates. Although crystal packing generated a non-physiological dimer, the monomeric fold aligns with known PIB and SERCA structures, reinforcing confidence in the biological relevance of the captured state.
The combined structural and comparative analyses strongly support that the crystallized MmCadA adopts an inward-open E1 conformation closely resembling that of PIB-1 ATPases. (Figures 1 and 2). The exposure of the conserved metal-binding residues in M4 and M6 toward the cytoplasm, together with the absence of bound metal in the M-domain, is characteristic of early E1 states and mirrors the behavior of SERCA in analogous intermediates. Structural superpositions further reinforce this assignment: both MmCadA monomers align closely with the inward-open E1 structure of AfCopA, displaying low RMSD values and nearly identical orientations of the A-, N-, and P-domains, whereas they diverge substantially from outward-open E2P states such as that of SsZntA. The pronounced interdomain rearrangements required for the E1/E2P transition, as well as the helix displacement patterns revealed by the transmembrane distance-difference matrix, match previously described two-bundle dynamics in PIB-1 ATPases, contrasting with the more complex behaviour of PII pumps like SERCA. The displacement of the A-domain relative to SERCA further reflects the absence of the A/M1 linker and the distinct communication logic of PIB enzymes. Together, these structural, topological, and comparative data coherently indicate that MmCadA is captured in an E1 inward-open state primed for metal uptake, consistent with the mechanistic architecture of PIB-1-type ATPases.
The structural data presented in Figure 3 also provide compelling evidence that MmCadA employs a wide, surface-exposed electronegative pathway to deliver Zn^{2+} directly to the conserved CPC motif, thereby refining previous mechanistic models of metal uptake in PIB-2 ATPases. The electrostatic surface representation (Fig. 3A) clearly reveals a continuous negatively charged cavity extending from the cytosolic environment toward the canonical binding site defined by C562, C564, and D889. In contrast to the “funnel” mechanism previously proposed for SsZntA, the putative gate residues M357 and F380 (Fig. 3B) are positioned far from this electronegative corridor and do not appear structurally capable of regulating ion access, which is consistent with mutagenesis data indicating minimal functional impact. Moreover, the identification of additional acidic residues (E354 and E387) adjacent to the CPC-site and positioned within coordination distance suggests the existence of a transitional uptake site, analogous, but not equivalent, to the Cu^{+}-entry platform described in PIB-1 ATPases such as AfCopA. Because Zn^{2+} trafficking lacks dedicated metallochaperones, the observed architectural features plausibly support a chaperone-independent diffusion-driven uptake mechanism. The persistence of the K868–D889 ion-pair across E1/E2 states (Fig. 3C–E) further hints at a direct structural coupling between early metal entry and catalytic transitions. Collectively, these findings convincingly support an inward-open, CPC-directed metal-uptake model specific to PIB-2 ATPases.
The combined biochemical, structural, and molecular dynamics data provide strong and internally consistent evidence that the N-terminal region of MmCadA fulfills multiple regulatory functions, including a distinct autoinhibitory role mediated by its distal modules. Functional assays of the deletion variants demonstrate that removal of MBD-2 or MBD-2 together with its adjacent histidine-rich region (His-2) markedly increases ATPase activity (up to fivefold relative to the wild type) despite leaving metal specificity largely intact. These results indicate that MBD-2/His-2 acts as an inhibitory module rather than a canonical metal-binding domain, a conclusion reinforced by the lack of functional impact of CXXC→AXXA substitutions. In contrast, deletion of the entire N-terminus severely reduces activity while increasing apparent Zn^{2+} affinity, highlighting the essential contribution of the proximal histidine-rich segment (His-1) to metal delivery or stabilization of the high-affinity binding site. Notably, retention of His-1 (as in the ΔMBD-2-His-2-MBD-1 variant) restores wild-type activity and affinity, supporting its role as a facilitator of uptake, analogous to metallochaperone-dependent mechanisms described for PIB-1-ATPases.
The MD simulations further substantiate this dual N-terminal functionality by revealing extensive contacts between MBD-2/His-2 and both the N- and P-domains in the wild-type protein. These interactions constrain domain flexibility, consistent with an autoinhibitory clamp that limits the conformational rearrangements required for the E1→E2 transition. In the ΔMBD-2-His-2 variant, this contact network collapses, resulting in increased RMSD and RMSF values for both soluble domains and a global relaxation of the ATPase core, mechanistically explaining the enhanced catalytic turnover observed biochemically. In contrast, His-1 and MBD-1 remain closely associated with the ion-uptake region, supporting their positive regulatory functions.
Collectively, the convergence of biochemical and computational analyses provides a compelling model in which the N-terminus integrates inhibitory and stimulatory layers of regulation to fine-tune metal transport. Nonetheless, the precise physical interfaces proposed by MD simulations warrant direct experimental validation, for example, via site-specific crosslinking, Förster resonance energy transfer (FRET), or targeted surface mutagenesis to definitively confirm the autoinhibitory contacts and refine the proposed mechanism.
Reviewers have the right to view the data and code that underlie the work if it would help in the evaluation, even if these have not been provided with the submission (see this Nature editorial). If essential data are not available, please contact the editor to obtain them before submitting the report.

Analytical approach
The bioinformatic analyses are clearly articulated and provide a solid foundation for the authors’ structural and mechanistic interpretations. Nevertheless, the quantitative datasets presented in Figure 1b, Figure 4c, and Supplementary Figure 11

would benefit from more comprehensive statistical reporting. Specifically, explicit information regarding the number of biological and technical replicates, the measures of variability employed (e.g., s.e.m. vs s.d.), and the exact statistical tests used to compare conditions is currently missing. Such details are essential for evaluating the robustness of the reported differences in apparent metal affinity among the variants, particularly in relation to the proposed functional role of the His²–MBD2 segment. Strengthening the statistical transparency of these analyses would substantially increase confidence in the conclusions and ensure that the mechanistic model is grounded not only in structural inference but also in quantitatively rigorous experimental evidence.

Suggested improvements
The metal-stimulated ATPase data indicate that MmCadA responds to Pb^{2+}, Zn^{2+} and Cd^{2+}, broadly matching the substrate profiles previously reported for PIB-type ATPases. However, as ATP hydrolysis can be activated independently of productive ion transport. Indeed, the activity pattern in Fig. 1B suggests that Pb^{2+} yields the highest Vmax, a behavior typical of soft metals that bind strongly to cysteine-rich motifs such as CPC, and thus potentially reflecting high-affinity but non-physiological interactions. The responses to Zn^{2+} and Cd^{2+} also show shallow activation curves that may reflect enzymatic stimulation rather than efficient translocation. As stated above, we strongly suggest that confirmative determination of the transported ion(s) will require intracellular metal quantification, such as atomic absorption or ICP-MS, following controlled metal challenges in whole cells.

Clarity and context
The text is presented with sufficient clarity, and the figures are well referenced throughout the manuscript, allowing the reader to easily connect the visual data with the corresponding analyses. The authors provide adequate contextualization of their results, drawing effectively on previous studies in the field that report similar conclusions or observations. This integration of prior work supports the interpretation of the findings and helps situate the manuscript within the broader body of knowledge on P1B-type ATPases, thereby enhancing the overall accessibility and coherence of the narrative.

References
The references are up-to-date, relevant and appropriately presented and adequately support the ideas discussed in the manuscript, reinforcing the contextualization of the findings within the existing literature.

Your expertise
We were able to assess the majority of the manuscript, including the structural, biochemical, and bioinformatic components. However, certain highly specialized aspects of the biophysical analyses, particularly those involving advanced spectroscopic parameters and their quantitative modeling, fall partially outside the scope of my expertise.

Additionally, we would also like to briefly answer the following suggested questions for the review process:

• What are the noteworthy results?
The most noteworthy result of the manuscript is the newly proposed transport mechanism for this class of proteins. This mechanistic model provides a significant conceptual advance by offering a clearer explanation of how P1B-2-type ATPases may coordinate metal translocation and regulate activity. It stands out as the key contribution of the study and has the potential to guide future research on the functional and structural diversity of these transporters.

• Will the work be of significance to the field and related fields? How does it compare to the established literature? If the work is not original, please provide relevant references.
The structural insight provided here appears genuinely novel, as inward-open conformations and N-terminal regulatory mechanisms of Zn^{2+}-transporting P-type ATPases remain sparsely characterized. While related themes exist, no prior studies report this specific combination of structural, biochemical, and mechanistic observations.

• Does the work support the conclusions and claims, or is additional evidence needed?
The dataset of results presented support the conclusions

• Are there any flaws in the data analysis, interpretation and conclusions? Do these prohibit publication or require revision?
There are no major flaws that would prohibit publication. However, certain datasets, particularly those in Figures 1b, 4c, and Supplementary Figure 11, would benefit from clearer statistical detail.

• Is the methodology sound? Does the work meet the expected standards in your field?
Yes, the methodology is sound and meets the expected standards for structural and functional studies of P-type ATPases. The combination of mutagenesis, transport assays, structural modeling, and bioinformatic analysis provides a comprehensive and methodologically solid foundation for the study.

• Is there enough detail provided in the methods for the work to be reproduced?
The methods are described with sufficient detail to allow reproducibility. Experimental procedures, constructs, and analytical approaches are adequately reported. Providing additional information on specific statistical analyses at figures ATPase activity, such as the tests used and sample sizes, would further enhance reproducibility but does not affect the general clarity of the methods.

Reviewer #3

(Remarks to the Author)
I co-reviewed this manuscript with one of the reviewers who provided the listed reports. This is part of the Nature Communications initiative to facilitate training in peer review and to provide appropriate recognition for Early Career Researchers who co-review manuscripts.

Reviewer #4

(Remarks to the Author)
The manuscript by Lyu et al. describes the crystal structure of an inward-open Zn-transporting ATPase (P-type ATPase family (subtype IB-2)). The A- and N-domain are poorly resolved and the N-terminal tail with the metal binding domains (MBDs) and some histidine rich segments are not resolved in the structure. No metal ions are bound to the M-domain wherefore the structure is proposed to be an early inward-open state, primed for metal ion uptake. The ATP mimetic, AMPPNP, was also not observed in the resolved structure.

Biochemical characterisation of constructs with mutations or deletions in the N-terminal part illustrate that deletion of the entire N-term tail reduces ATPase activity while simultaneously increasing metal affinity whereas retaining the nearest histidine-rich stretch retained, the properties remain similar to WT. The nearest histidine-rich segment of the N-terminal tail is proposed to donate metal ions to a cysteine residue a conserved CPC-motif, which in turn is proposed to be more exposed in the absence of the N-term segment.

On the other hand, it is suggested that the further away MBD and histidine rich region of the N-terminal tail are autoinhibitory and release the inhibition when the intracellular metal concentration increases and thus protects against toxicity.

The data is appealing, the proposed mechanisms new and interesting, the figures generally nice and the manuscript is well written.

However, while I appreciate the attempt to generate a structural hypothesis for the effect of the N-terminal part, I do have some concerns about the molecular dynamics section, listed below. I appreciate that there is some discussion of the structural insights into MBD position etc from line 17 onwards on page 14, but I think it should be mentioned when presenting the results of the simulations to ensure the reader is aware of the potential caveats from the get-go.

1) While the text does mention that the model used is from alphafold, there is no discussion of the reliability of the model in the modelling section. I looked it up online, and, not surprisingly, the long “spaghetti”/unstructured regions have “very low” confidence. Furthermore, the interaction region between MBD-1 and M2/M3 are low confidence regions, which I think should also be mentioned in the text.

2) It is suggested that the N-term region interacts with the N-domain. I assume it is the low-confidence unstructured part that moves over towards the N-domain. While this may be the explanation for the functional effect of the N-terminal section, I again think it should be underlined that this based on a model for which this region has very low confidence.

3) The histidine segment closest to the core ATPase is proposed to shield the electronegative update pathway – “At the same time, the histidine segment closest to the core (His-1) shields the electronegative uptake pathway, preventing unwanted turnover.” (page 12, line 17-18). However, Figure 4D illustrates His-1 seemingly pointing into the solvent, far away from the core ATPase? How is this reconciled?

4) Page 13, line 31-32: “histidine-rich sequences flanking the MBDs take center stage: His-2 plays a key role in autoinhibition, whereas His-1 is crucial for metal delivery”. Again, I think these are located in the “very low” confidence part of the alphafold structure? I would consider again highlighting that the structure of these parts is so far elusive. It is indeed very curious how something that appears so unstructured play such important roles though.

5) The simulations seem very short to me (100 ns), especially to sample the long unstructured N-terminal section. Again, I think the limitations should be stated. Did the authors consider generating a few different alphafold models (e.g. with the colab server) to get different input models for better sampling of the unstructured parts? Or at least to see whether there is any consistency in the models proposed by alphafold for the unstructured parts?

6) Fig 4D: legend states “representative structure from the molecular dynamics (MD) simulations of MmCadA highlighting the structural basis of autoinhibition”. In line with my comments above, I think it should be mentioned that this is only a potential mechanism.

7) Fig S12: The protein is rotated between A and B which could be highlighted in the legend or with a small symbol between the two panels. Also, for colourblind people, panel A will be very difficult to get much information from.

a. Panel C: I appreciate it is down to the huge error bars, but it seems counter intuitive to have -50 contacts. I am not sure I think it fair to describe this as “The simulation results reveal extensive contacts between the N-terminal region preceding the ATPase core and the A-domain both for the wild-type and the ΔMBD-2-His-2 truncation, indicating that the location of MBD-1 is independent of MBD-2-His-2 in the analyzed E1 state (Supplementary Fig. 12c)”?

b. Is “number of contacts” defined as atom pairs within 0.35 nm, or residue pairs (or something else)?

c. Panel S12J : How was a “representative structure” selected?

Other minor comments:

These two sentences confuse me a bit:
Page 3, line 28-29 “A few inward-open E1 structures exist but only of PIB-1-ATPases […] and no structure is available of an E1P conformation”
Page 4, line 9 “… first E1 structure of a PIB-ATPase involved in divalent ion transport ….”
Should the second sentence refer to the IB-type 2 group? Or is it the monovalent/divalent difference that is referred to (I am not familiar with how transporters of monovalent/divalent ions are distributed over the subfamilies)

Page 3, line 19: histidines mentioned twice
Page 14, line 19-20: “… downstream structural and mechanistic studies will be indispensable for fully dissecting how the enigmatic tails contribute to copper transport and …” Should this be *metal* transport and not just *copper* transport? Or, if copper transport is referred to on purpose, I think it is a bit unclear that the comparison now refers to copper transporters.

Figure S11: It might be worth mentioning in the legend that the y-axis differs between the plots.

The authors do not need to comment on this final point but I wanted to mention that I really like the idea of engineering organisms with hyperaccumulation capacity – I am however wondering how one would make the organisms tolerable to toxic levels of metals? Anyway, that is probably a different discussion.

Overall an interesting manuscript, but I suggest that the modelling part and conclusions/suggestions drawn from that section undergo major revisions as, while I think this part is also interesting and valueable, to me remains quite speculative.

Reviewer #5

(Remarks to the Author)
Comments regarding the X-ray crystallography/structural biology portion of the manuscript:

1) It would be preferable to include the Wilson B-factor in Supplementary Table 1, as well as display the B-factor colour plot against the refined model to further indicate to the reader that several portions of the model are flexible/disordered.
Average B-factor for protein and ligands should be indicated in the table.
In addition, for Supplementary Table 1, please include the temperature at which data collection was performed and the wavelength used.
Also, please indicate number of reflection in the highest resolution shell in parenthesis.

2) Are there any statistics that can inform us of per-chain/domain model fitting into the density maps? It may also be useful to include such information.

3) Phase recovery using ‘traditional models’ was unsuccessful (page 5, line 19). Could the authors expand a little on this? Furthermore, please include a figure of the AlphaFold structure used for molecular replacement.

4) The figure callout for the final structure indicates Fig.1b (page 5, line 21), but I think Fig.1c is correct?

5) Supplementary Figure 5; for the inset diffraction spots, please label the resolution. Also, please add a scale bar for the crystal picture.

6) It is feasible and reasonable that crystal packing effects could drive the monomer-monomer interaction, and as mentioned, the dimer formation may in fact disfavor the coupling between the P and M domains.
Yet, I am just curious, from your previous cryo-EM attempts did you ever see particles with such an arrangement, even at low resolution, or did you ever perform native-PAGE of the SEC fractions?

7) P12 L12 “the first metal-bound structure” is an overstatement. Authors do not show any densities corresponding to the heavy metal binding in the crystal structure. This is just E1-ATP state which shows high affinity for transport cargo. Since crystal was made in the presence of Pb-acetate, author should provide anomalous difference Fourier map to indicate Pb-binding position in the crystal structure (e.g., Abe et al., 2021, Nat Commun, DOI : 10.1038/s41467-021-26024-1).

Other comments

1) Page 9, line 16-18. It is mentioned that the K868-D889 interaction is retained in the E1 structure. It might be good to show the accompanying electron density (i know it was shown in Supp.Fig.6) of these residues within Fig.3. Also, monomer B K868 density is almost non-existent, so, it might be worth mentioning this?

2) Supplementary Figure 11; the activity measurements of the N-terminal truncation variants mention that 20 mM L-cysteine was included in the activity measurement. What is the role/purpose of adding cysteine to the reaction?

3) Supplementary Figure 9, please label the A, N and P domains.

4) Figure 3, please include a unit for the electrostatic colour scale.

5) Please indicate the membrane bilayer boundary in Figures 2 and 3.

Reviewer #6

(Remarks to the Author)
I co-reviewed this manuscript with one of the reviewers who provided the listed reports. This is part of the Nature Communications initiative to facilitate training in peer review and to provide appropriate recognition for Early Career Researchers who co-review manuscripts.

Version 1:

Reviewer comments:

Reviewer #1

(Remarks to the Author)
The authors adequately responded to all the technical comments, and the revised manuscript has significantly improved. While I am not fully convinced by the authors' arguments regarding the importance of their low-resolution structure, it is certainly a solid work. Whether it meets Nature Comms standards for impact and significance in view of the comments in my review of the original manuscript is an editorial decision.

Reviewer #2

(Remarks to the Author)
We thank the authors for their detailed responses to our comments. In our view, the revised manuscript has been substantially improved. The additional in vivo metal challenge assays and the intracellular Zn^{2+} quantification by ICP-MS significantly strengthen the functional interpretation and address my main concern regarding the distinction between metal-stimulated ATP hydrolysis and productive metal transport. The extended MD simulations, additional AlphaFold confidence analyses, and double-cysteine validation experiments also provide stronger support for the proposed N-terminal regulatory model.

Overall, we consider that the authors have satisfactorily addressed the major concerns raised in our previous review. The manuscript is now considerably stronger, both experimentally and conceptually.

We have only a few minor points that should be addressed before publication:

1- The authors should specify more clearly that direct intracellular metal quantification by ICP-MS was successfully performed for Zn^{2+}, whereas Cd^{2+} and Pb^{2+} transport are supported mainly by metal-resistance phenotypes. Thus, statements implying direct intracellular quantification for all three metals should be moderated.
2- The authors should avoid referring to the ATPase activity measurements as biological replicates if they were obtained from three independent measurements using material from a single protein purification. This should be described as independent technical measurements from one preparation, unless independent purifications were performed.
3- The term “zinc-specific” in the Abstract may be too restrictive given the demonstrated Zn^{2+}/Cd^{2+}/Pb^{2+} activity and detoxification phenotype. A more precise wording would better reflect the promiscuous metal response of MmCadA while maintaining Zn^{2+} as the likely physiological substrate.
4- The translational implications remain somewhat strong relative to the experimental scope. we recommend slightly softening the language in the Abstract and Conclusions to indicate that the findings may inform future biotechnological or translational applications, rather than directly supporting them.

These are minor textual and interpretative issues. we do not consider that additional experiments are required. Subject to these clarifications, we would support publication of the manuscript.

Reviewer #3

(Remarks to the Author)
I co-reviewed this manuscript with one of the reviewers who provided the listed reports. This is part of the Nature Communications initiative to facilitate training in peer review and to provide appropriate recognition for Early Career Researchers who co-review manuscripts.

Reviewer #4

(Remarks to the Author)
I thank the authors for their work on improving the manuscript based on comments from all reviewers (and for further satisfying my curiosity about how to make cells tolerate toxic levels of metals). I appreciate the much improved modelling section and the extended MD simulations, which has improved the former somewhat hand-wavy interpretations.

The authors have answered all my requests satisfactorily and I only have minor comments to the current manuscript version, as follows.

Fig 4 d: I am not sure whether a label for MBD-1 has been deliberately omitted? Also, I wonder whether rotating the protein a bit towards the right would make it easier to see that MBD-2 is indeed a bit more peripheral relative to MBD-1? From the current orientation, MBD-2 seems to almost partly overlap with MBD-1 (when zooming in, one of the beta strands of MBD-2 seems to cross the second from the top beta strand of MBD-1) – I appreciate that this is an artifact of the orientation, but a slightly rotated orientation might help the reader.

In the track-changes version of the manuscript, page 13, line 4: “Similar smearing was not observed in the double mutant H99C/S643C (His-2/P-5 domain), but was seen in the double mutant (Fig. 4e)”. This sentence seems confusing.

Suppl fig 14, legend for b) MmCad*A* not *a*. For panel G, I would adjust the range of the y-axis to match panels C and E.

Minor inconsistency: Reporting Summary; Software and code states that MD simulations used GROMACS 2021 whereas Materials and Methods states GROMACS 2023.3.

To comply with the section 4a of the molecular dynamics reproducibility checklist, the authors should include either the full box dimensions (preferably) or the number of water molecules used for solvation, along with the ion concentration. Currently the only information given is the number of lipid molecules along with the number of counterions wherefore the box dimensions and the ion concentration cannot be extracted, reducing the reproducibility.

There are inconsistencies between section 4d of the MD checklist: “The initial structure was obtained from the AlphaFold model available through UniProt (ID: I4IY19). However, the prepared simulation systems, simulation input files, and final coordinate files are not currently provided as supplementary material or in a public repository.” And the Data availability section of the manuscript: “All files required for producing the trajectories of the MD simulations can be found at repository: https://zenodo.org/XXXX.”

Reviewer #5

(Remarks to the Author)
Even if Pb was present in the crystallization solution, it is meaningless unless its density is clearly visible in the structure. Especially for this protein, which is known for its heavy-metal transport properties, the authors' description of the structure as 'the first metal-induced' could still mislead readers into believing that it captures the bound transport ion. And in fact, this is not the first structure of heavy-metal containing P1B-ATPase. Cu-bound P1B-ATPase structure has already been published previously from the authors' group (PDB 7R0H, 2022, Nat Commun)
Therefore, it must be emphasized that this structure represents nothing more than a standard E1·ATP state. There is no structural evidence to suggest that this structure resulted from the functional action of heavy metals. The authors should clarify this point to avoid overinterpretation.

Reviewer #6

(Remarks to the Author)
I co-reviewed this manuscript with one of the reviewers who provided the listed reports. This is part of the Nature Communications initiative to facilitate training in peer review and to provide appropriate recognition for Early Career Researchers who co-review manuscripts.

Open Access This Peer Review File is licensed under a Creative Commons Attribution 4.0 International License, which permits use, sharing, adaptation, distribution and reproduction in any medium or format, as long as you give appropriate credit to the original author(s) and the source, provide a link to the Creative Commons license, and indicate if changes were made.
In cases where reviewers are anonymous, credit should be given to 'Anonymous Referee' and the source.
The images or other third party material in this Peer Review File are included in the article's Creative Commons license, unless indicated otherwise in a credit line to the material. If material is not included in the article's Creative Commons license and your intended use is not permitted by statutory regulation or exceeds the permitted use, you will need to obtain permission directly from the copyright holder.
To view a copy of this license, visit https://creativecommons.org/licenses/by/4.0/

Point-to-points answers to the comments of the reviewers
(Comments from the reviewers in black. Our answers in red.)

Reviewer #1 (Remarks to the Author):

The authors present a low-resolution X-ray structure of a metal transporting P1B-ATPAse from Mesorhizobium metallidurans in E1 state. They also conduct activity assays with several truncation variants of the N-terminal region of the protein, which contains two metal-binding domains that are not resolved in the structure. These measurements together with molecular dynamics simulations lead them to the conclusion that the N-terminus plays an autoinhibitory role through the interactions with the A- or N-domain. The structure misses the metal-binding domains and does not show a metal bound in the transmembrane site. This, together with the low resolution of 3.6 A, limits the novelty of the structure. In fact, it seems that the same conclusions could be obtained simply by analyzing the AlphaFold model of the protein, which only differs by 2.3 A RMSD from the experimental structure, and most of the difference is due to a slight turn of the N-domain. Considering the generally high fidelity of AlphaFold models and already known architecture of P1B-ATPases, I don't think an experimental confirmation of the model is of much value by itself in this case.

We thank the reviewer for the thoughtful feedback. We agree that the experimental structure is overall similar to the AlphaFold prediction, and we have now included a direct comparison in the revised manuscript (Supplementary Figure 6). However, we respectfully disagree with the assertion that the experimental structure lacks intrinsic value. While AlphaFold models are highly informative, they remain computational predictions rather than experimentally validated observations, a distinction that continues to be broadly recognized in the structural biology community (PMID: 38036854, 39133843, 38907110).

In this context, the presented structure provides an experimentally determined framework for a divalent metal-transporting P1B-ATPase captured in a metal-induced E1 state. This is important because to our knowledge, previously reported structures of Zn-transporting P1B-ATPases have been limited to E2P and E2.Pi states, making this structure a distinct contribution in terms of conformational coverage. As such, the structure reveals how metal uptake is achieved, which differs from the mechanism observed in the related copper-transporting P-type ATPases. Importantly, the map allows visualization of side-chain conformations within the transmembrane (M) domain, offering structural detail that cannot be assumed a priori from prediction alone. Even at 3.6 Å resolution, these features help to validate key aspects of the protein architecture and provide a reliable basis for interpreting functional data.

Moreover, the structural data gain additional significance when integrated with the biochemical assays and molecular dynamics simulations presented in this study. Together, these complementary approaches support a coherent mechanistic model for ion uptake and N-terminal autoinhibition that would be difficult to substantiate from a predictive model alone. We have clarified these points in the revised manuscript to better emphasize the contribution of the experimental structure within the overall study.

1. Why is the protein called Zn2+ ATPase, if a much stronger affinity stimulation is observed with Pb2+ (Fig. 1B)?

While we observe higher activity with Pb(II) in Figure 1B, the protein is still classified as a Zn(II)-ATPase based on both historical convention and biological relevance. P1B-type ATPases are generally named according to their presumed physiological cargo, which for this subfamily (P_{1B-2}-type) is Zn(II). Intracellular Zn(II) concentrations are generally significantly higher than those of Pb(II) under normal physiological conditions, as Pb(II) is not a biologically required metal.

The elevated activity observed with Pb(II) likely reflects the enzyme's ability to recognize and efficiently transport toxic, non-physiological metals when they are present. From a biological perspective, this can be advantageous: if Pb(II) enters the cell, rapid export would be beneficial to mitigate toxicity without requiring dedicated Pb-specific systems. In this context, higher apparent activity with Pb(II) in vitro may reflect a detoxification capability rather than primary substrate preference. In addition, also the underlying metal coordination chemistry properties likely also contribute to the higher observed turnover with Pb(II). While both Zn(II) and Pb(II) are thiophilic metals, Pb(II) binding/release to thiolate ligands (e.g. to the CPC motif) is kinetically labile (despite being thermodynamically very favourable), potentially contributing to faster translocation kinetics.

2. A more detailed comparison of the experimental structure with the AlphaFold model used for molecular replacement is needed. What is the new knowledge gained from the experimental structure compared to the model, keeping in mind the low resolution of the structure and the fact that the general architecture of P1B-ATPases is well known from several previously published X-ray and Cryo-EM structures?

See also our response to the overall comments. We have now added a detailed comparison between the AlphaFold model and the experimental structure in Supplementary Figure 6. As noted in the Introduction, P-type ATPases undergo substantial conformational changes during their catalytic cycle, and hence multiple experimentally determined states are required to understand their transport mechanism. In contrast to the reviewer's statement, structural coverage for Zn(II)-transporting P1B-ATPases remains limited: to our knowledge, available experimental structures are restricted to the E2P state and the subsequent dephosphorylation transition state (E2.Pi). The structure presented here therefore expands the conformational landscape by providing an experimentally determined E1 state.

This is particularly relevant given that the arrangement of the cytosolic domains in the E1 state of Cu-transporting P1B-ATPases differs from that observed in other P-type ATPases, raising the question of whether this architecture is conserved across non-copper P1B-ATPases. Our structure directly addresses this point and provides an experimental basis for comparison. In addition, as highlighted in the Abstract, the putative metal uptake pathway observed here differs from that described for Cu-ATPases, further supporting mechanistic divergence within the family.

Finally, the experimental structure provides insights that are not captured by the AlphaFold model. For example, in the model, MBD^{-1} is positioned such that it reduces the apparent electronegativity of the metal-binding region in the transmembrane (M) domain. In contrast, our combined structural and functional data suggest that maintaining a more electronegative environment in this region may be important for activity - an aspect that would not be apparent from the AlphaFold prediction alone.

Together, these points underscore that the experimental structure does not merely confirm the model but provides new mechanistic and functional insights that are not accessible from prediction alone, despite the moderate resolution.

3. The structure shows an ATPase dimer. Does the protein purify as a dimer? SEC-MALS may be informative, even though the detergent may complicate accurate MW determination.

MmCadA displays preparation-dependent SEC profiles (Supplementary Fig. 4), where the main peak could be consistent with a dimeric or higher-order assembly, which may reflect a property of this protein under detergent-solubilized conditions.
Supporting the presence of higher oligomeric species, we also detect higher-molecular-weight assemblies by native PAGE (Supplementary Fig. 8), and in the crystal lattice of the present structure. However, we acknowledge that all three approaches have limitations in unambiguously defining stable, physiologically relevant oligomeric states for membrane proteins, particularly under non-native or crystallizing conditions.
Oligomerization of P-type ATPases remains a long-standing and unresolved question in the field. To our knowledge, there is no definitive evidence demonstrating that P1B-type ATPases function as oligomers during the catalytic cycle. At the same time, transient or condition-dependent interactions cannot be excluded based on available data, and crystal contacts may also reflect packing artefacts rather than biological assemblies.
Given the relatively tight interface observed in our crystal structure, we cannot fully exclude the possibility of functionally relevant inter-monomer interactions under certain conditions. However, based on the current data, we consider the monomer to be the primary functional unit, while any higher-order assembly remains speculative and potentially transient.

4. The most interesting aspect of the data shown in Fig. 4B is that the deletion of MBD-2-His-2 fragment appears to change metal specificity: unlike the wild type and other mutation and truncation variants, the activity of this construct is stimulated by Cd2+ and Zn2+ much stronger than by Pb2+. How can the autoinhibition model explain this?

We agree that this is an intriguing observation. In the ΔMBD-2–His-2 construct, the regulatory and inhibitory contributions of this module are removed, which results in a system that is no longer uniformly autoinhibited across all tested metal ions.
In the wild-type protein, MBD-2–His-2 appears to have a dual role: (i) it contributes to autoinhibition, reducing overall turnover, and (ii) it likely participates in modulating metal handling, potentially by influencing delivery or pre-organization of specific metal ions toward the transmembrane binding site. Upon removal of this region, these regulatory effects are lost. This leads to a differential effect on metal-dependent activity. For Pb(II), the increased affinity caused by removal of MBD-2–His-2 reduces the catalytic efficiency relative to Zn(II) and Cd(II), as binding may now be too tight for efficient transport. In contrast, Zn(II) and Cd(II) benefit more from the removal of autoinhibition, resulting in a stronger apparent stimulation of activity.
Thus, the data suggest that MBD-2–His-2 does not only act as an autoinhibitory element but may also fine-tune cargo selection and/or metal delivery efficiency, adding an additional layer of regulation to the transport cycle. This is an interesting aspect that we aim to explore further in future work.

5. What antibody was used for the Western blot in Sup. Fig. 3?

We employed an antibody against the strep-tag. This has been specified in the manuscript now.

6. Sup. Fig. 5: The inset appears to show crystals, rather than diffraction spots. Please check the legend.

The legend was unclear and has been revised to correctly describe the inset as showing crystals rather than diffraction spots.

7. Fig. 1B does not show a protein structure (p.5). Please check figure numbering.

This was a numbering error. The correct reference is Fig. 1C, and this has now been corrected.

8. What do the numbers shown in Sup. Table 2 mean? A more informative table title and/or legend is needed.

The numbers correspond to RMSD values between the indicated structural models. The table title and legend have been revised to explicitly state this.

9. "Despite the shortage of structural information on metal-uptake and -binding in PIB-type ATPases, previous biochemical studies have identified a single high-affinity Zn2+ binding site [60]". Reference 60 is incorrect here.

We thank the reviewer for pointing this out. The correct references are PMID: 22387457 and PMID: 37293658, and the citation has been updated accordingly.

10. "The metal likely coordinated by the cysteine thiolates of the conserved CPC motif of the M4 segment and two oxygen ligands from an invariant aspartate of M6, corresponding to C562, C564 and D889 in MmCadA (Supplementary Fig. 9a)" Figure reference is wrong.

The figure reference is correct. We intend to highlight that the CPC motif is conserved across multiple P1B-2 ATPases, as shown in the sequence alignment in Supplementary Fig. 9a.

11. "In this work we present the first metal-bound structure of a PIB-2-type ATPase, complemented by functional analysis." This is a misleading statement. There is indeed a bound lead atom in the structure, but judging from its location, it is of no functional significance.

We agree with the reviewer that this statement was misleading. We have revised the wording to "the first metal-induced structure" to better reflect the experimental conditions, as Pb(II) was present during crystallization. In addition, we have re-evaluated the electron density and removed the previously modeled Pb(II) ion, as the density does not support a confidently assigned bound metal at this site.

Reviewer #2 (Remarks to the Author):

Key results

Current models posit that bacterial P-type Zn^{2+}-ATPases operate through an alternating-access mechanism in which Zn^{2+} binds the conserved CPC motif from the cytosolic side, followed by ATP-driven phosphorylation events that occlude and subsequently outwardly expose the ion-binding site to enable Zn^{2+} release and prevent cytosolic toxicity. This work advances that framework by providing a coherent set of structural and functional data, most notably the first well-resolved inward-open conformation of a Zn^{2+}-transporting P-type ATPase, thereby experimentally clarifying how Zn^{2+} gains access to the CPC motif (Mesorhizobium metallidurans MmCadA). The integration of biochemical assays and molecular dynamics supports a regulatory model in which N-terminal metal-binding domains act as Zn^{2+}-responsive auto-inhibitory elements, and the identification of an electronegative uptake region together with a plausible histidine-mediated ion-donation pathway introduces regulatory and mechanistic features not captured in prevailing models. Although the manuscript overstates potential translational implications, the mechanistic insights are substantial, internally consistent, and scientifically meaningful.

Thank you for the very positive summary of our work! Following the suggestion of the reviewer we have toned down the final paragraph of the conclusions.

Validity

The experimental evidence presented, including targeted mutagenesis, detailed transport assays, molecular dynamics simulations, and supporting biophysical measurements, substantially reinforces the proposed mechanistic model and lends strong validity to the authors' conclusions. The structural and biochemical analyses are executed with commendable rigor, and the convergence of multiple, independent methodological approaches greatly enhances the robustness and credibility of the findings. Overall, the mechanistic framework advanced by the authors is both coherent and compelling, and it aligns convincingly with the structural features and functional behaviors documented throughout the study.

Significance

The manuscript presents conclusions with strong potential and substantial impact on the field of P-type ATPases. By identifying and characterizing structural features specific to P1B-2 ATPases, the study makes a meaningful contribution to our understanding of metal-transport pathways and the molecular determinants underlying substrate specificity and functional regulation.

Importantly, the proposal of a new transport mechanism represents a particularly valuable aspect of the work. Introducing a mechanistic model of this kind can significantly advance the field, as it provides novel hypotheses for how these proteins coordinate metal translocation and how structural elements modulate activity. Such mechanistic insights are especially relevant given that P1B-type ATPases are emerging as key molecular targets in the study of pathogenic organisms that rely on similar transporters during infection. By offering a refined structural and functional framework, the manuscript opens opportunities to better understand the role of these proteins in host–pathogen interactions and to explore their potential as targets for therapeutic intervention.

Overall, the findings have broad implications for structural biochemistry, membrane-transport biology, and the study of metal homeostasis, and they establish a strong foundation for future work on P-type ATPase function and evolution.

Data and methodology

The overexpression and purification strategy is broadly appropriate for studying bacterial Zn^{2+}-transporting P-type ATPases. Screening multiple PIB-2 candidates and selecting MmCadA based on expression is sound, and the membrane preparation, C12E8 solubilization, and Strep-based purification are methodologically solid. The mutagenesis strategy is well designed for probing regulatory elements. Expression in E. coli may also alter metalation or folding relative to the native system. Despite these concerns. Please, clarify this item. The strategy is conceptually appropriate and likely sufficient for structural and biochemical interrogation.

While we appreciate the reviewer's concern, we respectfully disagree that expression in E. coli represents a significant limitation in this case. The protein is clearly folded and functionally competent, as demonstrated by robust ATPase activity across multiple independent assays (Figures 1B, 4B, and 4C), as well as by protective effects observed in *in vivo* metal susceptibility assays reported in the revised version of the manuscript (*vide infra*). In addition, the overall architecture of the structure is consistent with previously determined P1B-type ATPase structures, supporting correct global folding.

Expression in E. coli is a well-established and widely used strategy for the biochemical and structural characterization of bacterial P-type ATPases, including members of the P1B subfamily. In many cases, heterologous expression is in fact necessary to obtain sufficient material for purification and structural studies, and has repeatedly yielded proteins that are both active and structurally faithful to their native counterparts.

We also note that the observed metal-dependent activity and regulatory behavior would be unlikely to arise from gross misfolding or non-native assembly. Instead, the consistency between functional assays, structural data, and known features of related ATPases supports that MmCadA adopts a native-like and functionally relevant conformation under the conditions used.

Taken together, we therefore consider the expression system appropriate for the aims of this study, and do not see evidence suggesting that folding or metalation is significantly compromised in a way that would affect the main conclusions.

The functional characterization strategy is, in principle, appropriate for probing metal-stimulated ATP hydrolysis in PIB-2-type ATPases. The Baginski assay is a valid choice for quantifying phosphate release, and the authors examine a broad panel of metal ions and use a systematic set of N-terminal mutants and truncations, which is conceptually aligned with dissecting regulatory and metal-handling roles.

The metal-stimulated ATPase data indicate that MmCadA responds to Pb^{2+}, Zn^{2+} and Cd^{2+}, broadly matching the substrate profiles previously reported for PIB-type ATPases. However, as ATP hydrolysis can be activated independently of productive ion transport. Indeed, the activity pattern in Fig. 1B suggests that Pb^{2+} yields the highest Vmax, a behavior typical of soft metals that bind strongly to cysteine-rich motifs such as CPC, and thus potentially reflecting high-affinity but non-physiological interactions. The responses to Zn^{2+} and Cd^{2+} also show shallow activation curves that may reflect enzymatic stimulation rather than efficient translocation. We strongly suggest that confirmative determination of the transported ion(s) will require intracellular metal quantification, such as atomic absorption or ICP-MS, following controlled metal challenges in whole cells.

We note the specific activity of the detergent-solubilized sample is comparable to what has been detected for other Zn-ATPases previously (PMID: 25132545 and 37293658), and hence we believe the risk for such artefacts is limited, perhaps even more so on a specific aspect of

the function such as autoinhibition. Nonetheless, we sincerely appreciate the reviewer's comment and suggestions. In agreement, we have performed cell growth assays upon metal exposure at increasing metal concentrations (new Supplementary Fig. 5 and additional main text), which unambiguously show that the recombinant expression of MmCadA in *E. coli* leads to increased cellular resistance against exposure to all metal substrates (Zn^{2+}, Cd^{2+} and Pb^{2+}). Growth-based metal susceptibility assays substantiate that MmCadA confers significant resistance under Zn^{2+}, Cd^{2+}-, and Pb^{2+}- stress conditions when compared to the D606N inactive mutant and control cells (Supplementary Fig. 5j–l), supporting a functional in vivo role in metal detoxification. Moreover, the concentration range and protective effect observed for Pb^{2+} (Supplementary Fig. 5l), is consistent with its broader intracellular reactivity and the higher concentrations required to induce toxicity in *E. coli*, as also reported in the literature (e.g., V. Adam et al., *Electrochim. Acta*, 2014). In addition, we also performed intracellular zinc quantification using ICP-MS demonstrating that *E. coli* cells expressing MmCadA feature reduced intracellular Zn^{2+} accumulation under high zinc exposure (Supplementary Fig. 5m), consistent with active CadA-mediated zinc efflux. To note, metal quantification was conducted also upon exposure to Cd^{2+}/Pb^{2+} but inconsistencies were observed due to intrinsic experimental incompatibilities. For example, under the conditions required for CadA expression (LB media, with IPTG induction) Pb^{2+} precipitation artifacts were observed preventing exact metal quantification arising from precipitation of insoluble Pb complexes with cell pellets, making the correct quantification of intracellular Pb^{2+} virtually impossible. Overall, as suggested by the reviewer, all the obtained additional *in vivo* data fully support the conclusion that MmCadA functions in cellular metal detoxification, with Zn^{2+} likely being its primary physiological endogenous substrate.

Regarding the protein crystallization, the protocol is generally sound and appropriate for membrane-protein crystallization, but key details are missing. Index, the SEC buffer composition, and final protein concentration in crystallization drops should be specified. Clarification of the rationale for Pb^{2+} incubation would also improve reproducibility and methodological clarification.

SEC buffer composition is specified in the last paragraph of "Overproduction and purification of MmCadA": "(20 mM MOPS-KOH, pH 8.0, 80 mM KCl, 20%(v/v) glycerol, 3 mM $MgCl_2$, 5 mM βME, 0.015% (w/v) $C_{12}E_8$)". Protein concentration in the crystallization drop has been added. We are unsure what the reviewer means by missing index?

The MD workflow is generally appropriate for exploring conformational differences between the wild-type protein and the ΔMBD/His-2 variant. The use of CHARMM-GUI for bilayer construction, POPC membranes of adequate size, TIP3P water, and GROMACS 2021 is technically sound. Replica exchange across closely spaced temperatures improves sampling, albeit modestly. The multi-step equilibration scheme is standard and correctly documented. However, several limitations reduce the mechanistic strength of the conclusions. First, relying on an AlphaFold model, particularly for a large membrane ATPase, introduces structural uncertainty that should be acknowledged or validated (e.g., via available homologous structures). Second, the 100 ns trajectories, even with three replicas, are relatively short for capturing domain rearrangements relevant to P-type ATPase regulation.

We thank the reviewer for this comment. While it is true that AlphaFold models can introduce uncertainty, in the present case this is mitigated by the strongly conserved architecture of P1B-type ATPases and the close agreement between the model and our experimentally determined structure, which supports the overall correctness of the fold. The main regions of higher

uncertainty are confined to the N-terminal extensions, including the MBD-containing segments. Importantly, there is very limited experimental structural information available for these regions, making the model a reasonable and currently necessary starting point.

Nonetheless, we agree that both the quality of the starting structure and sufficient conformational sampling are critical for robust mechanistic conclusions. In the revised manuscript, we have addressed these points in two ways.

First, we extended all production MD simulations from 100 ns to 500 ns per replica. This substantial increase in sampling improves the exploration of conformational space and provides a more reliable basis for evaluating domain rearrangements relevant to P-type ATPase regulation.

Second, we more rigorously assessed the robustness of the starting structures. We evaluated confidence metrics (PAE and pLDDT) for the full-length MmCadA model (UniProt: I4IY19), confirming high confidence in the core ATPase domains and localized uncertainty in the flexible N-terminal region. In addition, we generated five independent AlphaFold models using different random seeds and full multiple sequence alignments derived from comprehensive sequence databases. Comparison of these models shows a high degree of structural consistency across the core domains, supporting the stability of the starting conformation used in the simulations.

The structural characterization of the PIB-2 ATPase MmCadA combines experimental crystallography with complementary bioinformatic modelling to elucidate its architecture and conformational state. Crystals obtained under conditions favoring an inward-open E1 conformation, high lipid and detergent content, Pb^{2+}, Mg^{2+} and AMPPNP, enabled the determination of the structure at 3.6 Å via molecular replacement using an AlphaFold-predicted model. The resulting structure reveals a conserved P-type ATPase core comprising the A-, P- and N-domains and an eight-helix transmembrane region (MA–M6), whereas the flexible N-terminal MBDs and histidine-rich segments are unresolved, consistent with their intrinsic disorder. The well-defined P- and M-domains support the assignment of an early metal-uptake state, while subtle intra-domain shifts suggest conformational heterogeneity typical of transport intermediates. Although crystal packing generated a non-physiological dimer, the monomeric fold aligns with known PIB and SERCA structures, reinforcing confidence in the biological relevance of the captured state.

The combined structural and comparative analyses strongly support that the crystallized MmCadA adopts an inward-open E1 conformation closely resembling that of PIB-1 ATPases. (Figures 1 and 2). The exposure of the conserved metal-binding residues in M4 and M6 toward the cytoplasm, together with the absence of bound metal in the M-domain, is characteristic of early E1 states and mirrors the behavior of SERCA in analogous intermediates. Structural superpositions further reinforce this assignment: both MmCadA monomers align closely with the inward-open E1 structure of AfCopA, displaying low RMSD values and nearly identical orientations of the A-, N-, and P-domains, whereas they diverge substantially from outward-open E2P states such as that of SsZntA. The pronounced interdomain rearrangements required for the E1/E2P transition, as well as the helix displacement patterns revealed by the transmembrane distance-difference matrix, match previously described two-bundle dynamics in PIB-1 ATPases, contrasting with the more complex behaviour of PII pumps like SERCA. The displacement of the A-domain relative to SERCA further reflects the absence of the A/M1 linker and the distinct communication logic of PIB enzymes. Together, these structural, topological, and comparative data coherently indicate that MmCadA is captured in an E1 inward-open state primed for metal uptake, consistent with the mechanistic architecture of PIB-1-type ATPases.

The structural data presented in Figure 3 also provide compelling evidence that MmCadA employs a wide, surface-exposed electronegative pathway to deliver Zn^{2+} directly to the conserved CPC motif, thereby refining previous mechanistic models of metal uptake in PIB-2 ATPases. The electrostatic surface representation (Fig. 3A) clearly reveals a continuous negatively charged cavity extending from the cytosolic environment toward the canonical binding site defined by C562, C564, and D889. In contrast to the "funnel" mechanism previously proposed for SsZntA, the putative gate residues M357 and F380 (Fig. 3B) are positioned far from this electronegative corridor and do not appear structurally capable of regulating ion access, which is consistent with mutagenesis data indicating minimal functional impact. Moreover, the identification of additional acidic residues (E354 and E387) adjacent to the CPC-site and positioned within coordination distance suggests the existence of a transitional uptake site, analogous, but not equivalent, to the Cu^{+}-entry platform described in PIB-1 ATPases such as AfCopA. Because Zn^{2+} trafficking lacks dedicated metallochaperones, the observed architectural features plausibly support a chaperone-independent diffusion-driven uptake mechanism. The persistence of the K868–D889 ion-pair across E1/E2 states (Fig. 3C–E) further hints at a direct structural coupling between early metal entry and catalytic transitions. Collectively, these findings convincingly support an inward-open, CPC-directed metal-uptake model specific to PIB-2 ATPases.

The combined biochemical, structural, and molecular dynamics data provide strong and internally consistent evidence that the N-terminal region of MmCadA fulfills multiple regulatory functions, including a distinct autoinhibitory role mediated by its distal modules. Functional assays of the deletion variants demonstrate that removal of MBD-2 or MBD-2 together with its adjacent histidine-rich region (His-2) markedly increases ATPase activity (up to fivefold relative to the wild type) despite leaving metal specificity largely intact. These results indicate that MBD-2/His-2 acts as an inhibitory module rather than a canonical metal-binding domain, a conclusion reinforced by the lack of functional impact of CXXC→AXXA substitutions. In contrast, deletion of the entire N-terminus severely reduces activity while increasing apparent Zn^{2+} affinity, highlighting the essential contribution of the proximal histidine-rich segment (His-1) to metal delivery or stabilization of the high-affinity binding site. Notably, retention of His-1 (as in the ΔMBD-2-His-2-MBD-1 variant) restores wild-type activity and affinity, supporting its role as a facilitator of uptake, analogous to metallochaperone-dependent mechanisms described for PIB-1-ATPases.

The MD simulations further substantiate this dual N-terminal functionality by revealing extensive contacts between MBD-2/His-2 and both the N- and P-domains in the wild-type protein. These interactions constrain domain flexibility, consistent with an autoinhibitory clamp that limits the conformational rearrangements required for the E1→E2 transition. In the ΔMBD-2-His-2 variant, this contact network collapses, resulting in increased RMSD and RMSF values for both soluble domains and a global relaxation of the ATPase core, mechanistically explaining the enhanced catalytic turnover observed biochemically. In contrast, His-1 and MBD-1 remain closely associated with the ion-uptake region, supporting their positive regulatory functions.

Collectively, the convergence of biochemical and computational analyses provides a compelling model in which the N-terminus integrates inhibitory and stimulatory layers of regulation to fine-tune metal transport. Nonetheless, the precise physical interfaces proposed by MD simulations warrant direct experimental validation, for example, via site-specific

crosslinking, Förster resonance energy transfer (FRET), or targeted surface mutagenesis to definitively confirm the autoinhibitory contacts and refine the proposed mechanism.

The MD simulations have been further supplemented by double cysteine mutations to assess interactions between domains (inserted in Fig. 4), suggesting interaction between His^{-2} and the A-domain, with no interaction detected with the P-domain.

Analytical approach

The bioinformatic analyses are clearly articulated and provide a solid foundation for the authors' structural and mechanistic interpretations. Nevertheless, the quantitative datasets presented in Figure 1b, Figure 4c, and Supplementary Figure 11 would benefit from more comprehensive statistical reporting. Specifically, explicit information regarding the number of biological and technical replicates, the measures of variability employed (e.g., s.e.m. vs s.d.), and the exact statistical tests used to compare conditions is currently missing. Such details are essential for evaluating the robustness of the reported differences in apparent metal affinity among the variants, particularly in relation to the proposed functional role of the His^2–MBD2 segment. Strengthening the statistical transparency of these analyses would substantially increase confidence in the conclusions and ensure that the mechanistic model is grounded not only in structural inference but also in quantitatively rigorous experimental evidence.

We agree that clear reporting of statistical methodology is important for interpretation. As can be seen in the figure legends for Figure 1B, Figure 4C, and Supplementary Figure 11, the experiments were performed from three independent measurements using material derived from a single protein purification, which specifies both the technical and biological replication level in this experimental setup. We agree, however, that this information should be more explicitly emphasized in the main text and has therefore been clarified in the revised manuscript.

In addition, we have revised the description of variability to improve statistical transparency: all instances of "standard error of the mean" have been corrected to "standard deviation," ensuring that the reported error bars are accurately and consistently defined across all relevant figures.

Suggested improvements

The metal-stimulated ATPase data indicate that MmCadA responds to Pb^{2+}, Zn^{2+} and Cd^{2+}, broadly matching the substrate profiles previously reported for PIB-type ATPases. However, as ATP hydrolysis can be activated independently of productive ion transport. Indeed, the activity pattern in Fig. 1B suggests that Pb^{2+} yields the highest Vmax, a behavior typical of soft metals that bind strongly to cysteine-rich motifs such as CPC, and thus potentially reflecting high-affinity but non-physiological interactions. The responses to Zn^{2+} and Cd^{2+} also show shallow activation curves that may reflect enzymatic stimulation rather than efficient translocation. As stated above, we strongly suggest that confirmative determination of the transported ion(s) will require intracellular metal quantification, such as atomic absorption or ICP-MS, following controlled metal challenges in whole cells.

Again, we note the specific activity of the detergent-solubilized sample is comparable to what has been detected for other Zn-ATPases previously (PMID: 25132545 and 37293658), and hence we believe the risk for such artefacts is limited. Moreover, in our view, ATP turn-over is generally tightly coupled to cellular work, such as transport in this particular case. Nonetheless, we have performed growth assays upon metal exposure (new Supplementary Fig. 5 and

additional main text), which show that the presence of MmCadA increases metal resistance and lowers intracellular Zn^{2+} concentrations upon exposure to metal challenges.

Clarity and context
The text is presented with sufficient clarity, and the figures are well referenced throughout the manuscript, allowing the reader to easily connect the visual data with the corresponding analyses. The authors provide adequate contextualization of their results, drawing effectively on previous studies in the field that report similar conclusions or observations. This integration of prior work supports the interpretation of the findings and helps situate the manuscript within the broader body of knowledge on P1B-type ATPases, thereby enhancing the overall accessibility and coherence of the narrative.

References
The references are up-to-date, relevant and appropriately presented and adequately support the ideas discussed in the manuscript, reinforcing the contextualization of the findings within the existing literature.

Your expertise
We were able to assess the majority of the manuscript, including the structural, biochemical, and bioinformatic components. However, certain highly specialized aspects of the biophysical analyses, particularly those involving advanced spectroscopic parameters and their quantitative modeling, fall partially outside the scope of my expertise.

Additionally, we would also like to briefly answer the following suggested questions for the review process:
• What are the noteworthy results?
The most noteworthy result of the manuscript is the newly proposed transport mechanism for this class of proteins. This mechanistic model provides a significant conceptual advance by offering a clearer explanation of how P1B-2-type ATPases may coordinate metal translocation and regulate activity. It stands out as the key contribution of the study and has the potential to guide future research on the functional and structural diversity of these transporters.

We thank the reviewer for the positive evaluation of our work.

• Will the work be of significance to the field and related fields? How does it compare to the established literature? If the work is not original, please provide relevant references.

The structural insight provided here appears genuinely novel, as inward-open conformations and N-terminal regulatory mechanisms of Zn^{2+}-transporting P-type ATPases remain sparsely characterized. While related themes exist, no prior studies report this specific combination of structural, biochemical, and mechanistic observations.

• Does the work support the conclusions and claims, or is additional evidence needed?
The dataset of results presented support the conclusions.

• Are there any flaws in the data analysis, interpretation and conclusions? Do these prohibit publication or require revision?
There are no major flaws that would prohibit publication. However, certain datasets, particularly those in Figures 1b, 4c, and Supplementary Figure 11, would benefit from clearer statistical detail.

See our previous response to this reviewer concerning this issue.

• Is the methodology sound? Does the work meet the expected standards in your field?
Yes, the methodology is sound and meets the expected standards for structural and functional studies of P-type ATPases. The combination of mutagenesis, transport assays, structural modeling, and bioinformatic analysis provides a comprehensive and methodologically solid foundation for the study.

• Is there enough detail provided in the methods for the work to be reproduced?
The methods are described with sufficient detail to allow reproducibility. Experimental procedures, constructs, and analytical approaches are adequately reported. Providing additional information on specific statistical analyses at figures ATPase activity, such as the tests used and sample sizes, would further enhance reproducibility but does not affect the general clarity of the methods.

Reviewer #3 (Remarks to the Author):

I co-reviewed this manuscript with one of the reviewers who provided the listed reports. This is part of the Nature Communications initiative to facilitate training in peer review and to provide appropriate recognition for Early Career Researchers who co-review manuscripts.

Thank you for your assistance with improving the manuscript.

Reviewer #4 (Remarks to the Author):

The manuscript by Lyu et al. describes the crystal structure of an inward-open Zn-transporting ATPase (P-type ATPase family (subtype IB-2)). The A- and N-domain are poorly resolved and the N-terminal tail with the metal binding domains (MBDs) and some histidine rich segments are not resolved in the structure. No metal ions are bound to the M-domain wherefore the structure is proposed to be an early inward-open state, primed for metal ion uptake. The ATP mimetic, AMPPNP, was also not observed in the resolved structure.

Biochemical characterisation of constructs with mutations or deletions in the N-terminal part illustrate that deletion of the entire N-term tail reduces ATPase activity while simultaneously increasing metal affinity whereas retaining the nearest histidine-rich stretch retained, the properties remain similar to WT. The nearest histidine-rich segment of the N-terminal tail is proposed to donate metal ions to a cysteine residue a conserved CPC-motif, which in turn is proposed to be more exposed in the absence of the N-term segment.

On the other hand, it is suggested that the further away MBD and histidine rich region of the N-terminal tail are autoinhibitory and release the inhibition when the intracellular metal concentration increases and thus protects against toxicity.

The data is appealing, the proposed mechanisms new and interesting, the figures generally nice and the manuscript is well written.

Thank you for the very positive summary of our work!

However, while I appreciate the attempt to generate a structural hypothesis for the effect of the N-terminal part, I do have some concerns about the molecular dynamics section, listed below. I appreciate that there is some discussion of the structural insights into MBD position etc from line 17 onwards on page 14, but I think it should be mentioned when presenting the results of the simulations to ensure the reader is aware of the potential caveats from the get-go.

We have hopefully addressed your concerns in the following sections.

1) While the text does mention that the model used is from alphafold, there is no discussion of the reliability of the model in the modelling section. I looked it up online, and, not surprisingly, the long "spaghetti"/unstructured regions have "very low" confidence. Furthermore, the interaction region between MBD-1 and M2/M3 are low confidence regions, which I think should also be mentioned in the text.

We thank the reviewer for raising this important point. We agree that the confidence of the AlphaFold model should be explicitly addressed. In the revised manuscript, we now include an analysis of both the Predicted Aligned Error (PAE) and per-residue pLDDT scores for the wild-type MmCadA model obtained from UniProt (ID: I4IY19). As noted by the reviewer, the extended "spaghetti-like" regions show low confidence and are now described as likely flexible or disordered segments. We now explicitly acknowledge these regions should be interpreted with caution. To further assess robustness, we compared the reference model with multiple independently generated models using AlphaFold2 (different random seeds), which show consistent folding of the protein.

2) It is suggested that the N-term region interacts with the N-domain. I assume it is the low-confidence unstructured part that moves over towards the N-domain. While this may be the explanation for the functional effect of the N-terminal section, I again think it should be underlined that this based on a model for which this region has very low confidence.

We have edited this section to point out the uncertainty caused by the low confidence model. Moreover, in the revised manuscript, we have attempted to avoid over-interpretation of interactions between the N-terminal and the N-domain. We have also re-run all simulations to 500 ns per replica, which improved sampling and allowed us to focus on more stable, persistent and consistent interactions. In this context, we now emphasize only those contacts that are consistently observed during the simulations, rather than transient or variable interactions.

3) The histidine segment closest to the core ATPase is proposed to shield the electronegative update pathway – "At the same time, the histidine segment closest to the core (His-1) shields the electronegative uptake pathway, preventing unwanted turnover." (page 12, line 17-18). However, Figure 4D illustrates His-1 seemingly pointing into the solvent, far away from the core ATPase? How is this reconciled?

We conclude that this conclusion was drawn a bit hastily. In the revised manuscript, we have re-run all simulations to 500 ns per replica, so, we have updated Figure 4D by replacing the previous representative snapshot with a centroid MmCadA structure from the RMSD-based cluster corresponding to 20% of the molecular dynamics (MD) simulation frames. Collectively, it appears more likely that the His^{-1} segment is involved in assisting with metal delivery to the high-affinity site, which would account for its position in the model and the lowered activity (despite lowered affinity) when deleted. We have modified the text to reflect this.

4) Page 13, line 31-32: "histidine-rich sequences flanking the MBDs take center stage: His-2 plays a key role in autoinhibition, whereas His-1 is crucial for metal delivery". Again, I think these are located in the "very low" confidence part of the alphafold structure? I would consider again highlighting that the structure of these parts is so far elusive. It is indeed very curious how something that appears so unstructured play such important roles though.

We agree that this is curious. While it is known that crucial function and lower flexibility are not necessarily linked, such as in intrinsically disordered proteins, it has not been studied in P-type ATPases. We have added a line pointing out the high flexibility (and thus implied lower confidence) of these regions in the indicated section of the text.

5) The simulations seem very short to me (100 ns), especially to sample the long unstructured N-terminal section. Again, I think the limitations should be stated. Did the authors consider generating a few different alphafold models (e.g. with the colab server) to get different input models for better sampling of the unstructured parts? Or at least to see whether there is any consistency in the models proposed by alphafold for the unstructured parts?

We thank the reviewer for this useful comment. We agree that 100 ns is relatively short, and therefore we have re-run the simulations to 500 ns per replica. We also note that this region has low confidence in the AlphaFold2 model and is expected to be highly dynamic. In addition, we generated multiple independent AlphaFold models (using different seeds and full database searches) to assess structural consistency.

6) Fig 4D: legend states "representative structure from the molecular dynamics (MD) simulations of MmCadA highlighting the structural basis of autoinhibition". In line with my comments above, I think it should be mentioned that this is only a potential mechanism.

This has been edited in the figure legend, along with previously mentioned changes to the main text to emphasise model uncertainty.

7) Fig S12: The protein is rotated between A and B which could be highlighted in the legend or with a small symbol between the two panels. Also, for colourblind people, panel A will be very difficult to get much information from.

We have now addressed these concerns in the revised manuscript.

a. Panel C: I appreciate it is down to the huge error bars, but it seems counter intuitive to have -50 contacts. I am not sure I think it fair to describe this as "The simulation results reveal extensive contacts between the N-terminal region preceding the ATPase core and the A-domain both for the wild-type and the ΔMBD-2-His-2 truncation, indicating that the location of MBD-1 is independent of MBD-2-His-2 in the analyzed E1 state (Supplementary Fig. 12c)"?

The negative values were not real contacts, but came from plotting the mean with standard deviation (SD). We have now updated the analysis to use the standard error of the mean (SEM) instead of SD and ensured the values are kept ≥ 0. This removes the non-physical negative values while keeping the correct uncertainty, thanks for the comment!

b. Is "number of contacts" defined as atom pairs within 0.35 nm, or residue pairs (or something else)?

All contact numbers were calculated based on atom pairs within 0.35 nm.

c. Panel S12J : How was a "representative structure" selected?

The representative structure has been updated and represents a centroid structure from the RMSD-based cluster (cutoff of 0.2 nm) corresponding to 20% of the molecular dynamics (MD) simulation frames

Other minor comments:

These two sentences confuse me a bit:

Page 3, line 28-29 "A few inward-open E1 structures exist but only of PIB-1-ATPases […] and no structure is available of an E1P conformation"

Page 4, line 9 "… first E1 structure of a PIB-ATPase involved in divalent ion transport …."

Should the second sentence refer to the IB-type 2 group? Or is it the monovalent/divalent difference that is referred to (I am not familiar with how transporters of monovalent/divalent ions are distributed over the subfamilies).

This refers to the fact that all currently available E1 structures are of P_{IB-1} ATPases that transport monovalent copper (the text has been edited to include this). This, instead is the first

E1 structure of any P_{1B}-ATPase that transports divalent metal ions, which several other P_{1B} subfamilies do.

Page 3, line 19: histidines mentioned twice

This is intentional, as the second "histidines" is part of a group with cysteines and aspartic acid. We have edited the text for increased clarity.

Page 14, line 19-20: "… downstream structural and mechanistic studies will be indispensable for fully dissecting how the enigmatic tails contribute to copper transport and …" Should this be *metal* transport and not just *copper* transport? Or, if copper transport is referred to on purpose, I think it is a bit unclear that the comparison now refers to copper transporters.

We agree that this should refer to metal transport, and have edited the text accordingly.

Figure S11: It might be worth mentioning in the legend that the y-axis differs between the plots.

This has been amended.

The authors do not need to comment on this final point but I wanted to mention that I really like the idea of engineering organisms with hyperaccumulation capacity – I am however wondering how one would make the organisms tolerable to toxic levels of metals? Anyway, that is probably a different discussion.

We agree that this is a very interesting prospect. For eukaryotic cells, one option would be to ensure that the hyperaccumulated metals are sequestered in vacuoles or similar intracellular compartments to reduce their effect on organism viability. Insights can also be drawn from engineered metal-sensitive strains, which highlight the importance of specific resistance pathways. For instance, the deletion of zinc efflux systems *zitB* and z*ntA* in the Zn-sensitive *E. coli* strain GG48 (https://doi.org/10.1016/j.resmic.2014.02.001). The deletion of copper efflux systems *copA* and *cusCFBA* but also multicopper oxidase gene *cueO* in the Cu-sensitive *E. coli* strain ECA464 (10.1128/AEM.02842-14), Also, the introduction of storage systems like metal chaperones and storage metalloprotein, such as metallothioneins, are also effective system in cells to deal with metal accumulation (https://doi.org/10.1016/j.electacta.2014.06.091). In conclusion, a hyperaccumulation system would exploit a combination of these three mechanisms: efflux, oxidative stress protection, and storage/chaperoning.

Overall an interesting manuscript, but I suggest that the modelling part and conclusions/suggestions drawn from that section undergo major revisions as, while I think this part is also interesting and valueable, to me remains quite speculative.

We thank you for the positive evaluation and hope that we revised version of the manuscript will handle the critique from the reviewer.

Reviewer #5 (Remarks to the Author):

Comments regarding the X-ray crystallography/structural biology portion of the manuscript:

1) It would be preferable to include the Wilson B-factor in Supplementary Table 1, as well as display the B-factor colour plot against the refined model to further indicate to the reader that several portions of the model are flexible/disordered. Average B-factor for protein and ligands should be indicated in the table. In addition, for Supplementary Table 1, please include the temperature at which data collection was performed and the wavelength used. Also, please indicate number of reflection in the highest resolution shell in parenthesis.

Thank you for pointing this out. The requested information has been added to Table 1, and a figure colored by b-factor included in Supplementary fig. 8.

2) Are there any statistics that can inform us of per-chain/domain model fitting into the density maps? It may also be useful to include such information.

While there are statistical tools available to quantify per-chain or per-domain fit to the density, we did not include a detailed breakdown of such analyses in the present manuscript. The primary reason is that, in this case, the independently fitted monomeric model shows a highly consistent and uniform agreement with the electron density across the structured regions, with no indication of domain-specific deviations that would necessitate additional segmentation-level validation.

Given the overall low-to-moderate resolution of the data (3.6 Å) and the strong structural similarity between the model and the map, we focused on global refinement statistics and overall map-model agreement metrics, which are the most meaningful and robust descriptors at this resolution regime. We therefore consider additional per-domain statistical decomposition unlikely to provide further interpretative value beyond what is already evident from the refined model and the presented density.

3) Phase recovery using 'traditional models' was unsuccessful (page 5, line 19). Could the authors expand a little on this? Furthermore, please include a figure of the AlphaFold structure used for molecular replacement.

We agree that this was unclear, and have replaced "traditional models" with "previously determined, homologous P_{IB}-type ATPase structures". The AlphaFold model used for molecular replacement has been added in the new Supplementary figure 6.

4) The figure callout for the final structure indicates Fig.1b (page 5, line 21), but I think Fig.1c is correct?

It should be figure 1c, this has been corrected in the revised version of the manuscript.

5) Supplementary Figure 5; for the inset diffraction spots, please label the resolution. Also, please add a scale bar for the crystal picture.

The resolution for the inset diffraction spots has now been addressed in the figure legend and a scale bar for the crystal has been added.

6) It is feasible and reasonable that crystal packing effects could drive the monomer-monomer interaction, and as mentioned, the dimer formation may in fact disfavor the coupling between the P and M domains. Yet, I am just curious, from your previous cryo-EM attempts did you ever see particles with such an arrangement, even at low resolution, or did you ever perform native-PAGE of the SEC fractions?

While we have performed cryo-EM previously, these attempts were not successful, and the particle quality was insufficient to determine dimer or monomer arrangement, as can be seen from micrographs and 2D-classes inserted here for the reviewer's consideration (Fig 1). In native-PAGE there are bands indicating a dimer, and possibly also a tetramer, as added in supplementary figure 8, and the size of the SEC peak may also indicate a dimer. However, based on our and previous data there is little to suggest functional relevance of such oligomers.

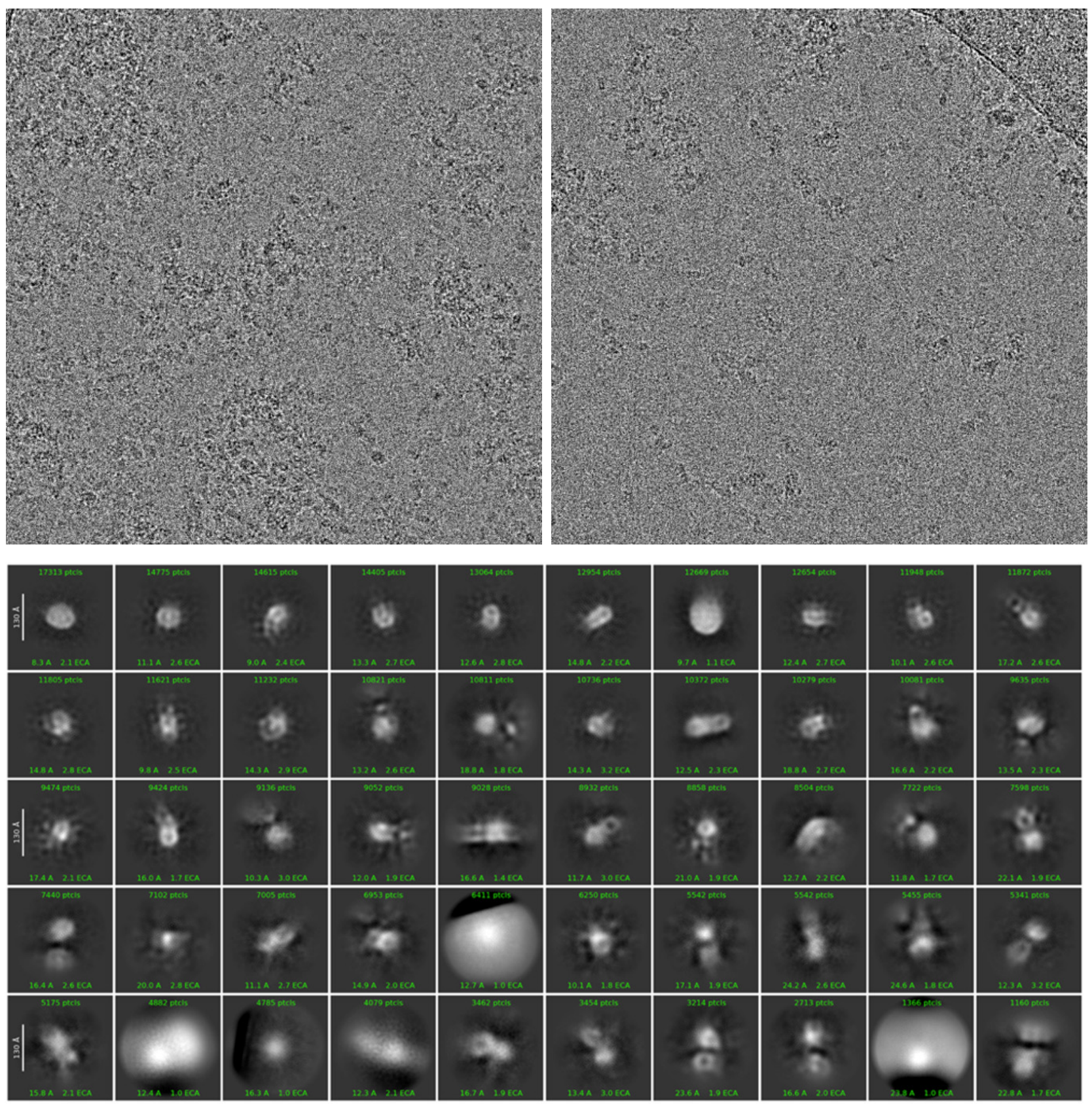

Figure 1 Cryo-EM attempts for MmCadA, with representative micrographs (top) and associated 2D classes (bottom).

7) P12 L12 "the first metal-bound structure" is an overstatement. Authors do not show any densities corresponding to the heavy metal binding in the crystal structure. This is just E1-ATP state which shows high affinity for transport cargo. Since crystal was made in the presence of Pb-acetate, author should provide anomalous difference Fourier map to indicate Pb-binding position in the crystal structure (e.g., Abe et al., 2021, Nat Commun, DOI : 10.1038/s41467-021-26024-1).

Upon further analysis for the data, we agree with the reviewer that this statement is misleading, and have revised it to "the first metal-induced" structure, as Pb was present during

crystallisation. We have also removed the lead previously seen in the structure, as the density is not strong enough to support this placement.

Other comments

1) Page 9, line 16-18. It is mentioned that the K868-D889 interaction is retained in the E1 structure. It might be good to show the accompanying electron density (i know it was shown in Supp.Fig.6) of these residues within Fig.3. Also, monomer B K868 density is almost non-existent, so, it might be worth mentioning this?

We have revised the Results section to more explicitly describe the difference in electron density for K868 between the two monomers, including the substantially weaker density observed for monomer B. This clarification helps better reflect the level of confidence in side-chain placement across the two protomers.
Regarding the suggestion to include the corresponding electron density in Figure 3, we consider that adding these maps to the main figure would introduce additional visual complexity and reduce clarity, particularly given the already dense structural annotation in Figure 3. We therefore opted to retain the density in the supplementary material and instead strengthen the cross-referencing in the main text to guide the reader to the relevant evidence.
We believe this approach preserves both transparency and readability, while still clearly documenting the differing local confidence in the two monomers.

2) Supplementary Figure 11; the activity measurements of the N-terminal truncation variants mention that 20 mM L-cysteine was included in the activity measurement. What is the role/purpose of adding cysteine to the reaction?

We acknowledge that the inclusion of 20 mM L-cysteine in these assays is not required for the Zn(II)/Cd(II)/Pb(II)-dependent activity measurements reported here. The reason is historical: in our laboratory, L-cysteine has been routinely included in ATPase activity assays developed for Cu-transporting P1B-type ATPases, where it helps maintain copper in the appropriate redox state and prevents non-specific oxidation effects.

During adaptation of this protocol to the present Zn(II)-ATPase system, this component was inadvertently retained even though it is not functionally necessary for divalent non-redox-active metals. Importantly, because all variants were measured under identical conditions, its presence does not affect the relative comparisons or the conclusions drawn from the truncation analysis.

3) Supplementary Figure 9, please label the A, N and P domains.

This has been amended.

4) Figure 3, please include a unit for the electrostatic colour scale.

The unit and source for calculating the image has been added to the figure legend.

5) Please indicate the membrane bilayer boundary in Figures 2 and 3.

Membrane bilayer boundaries have been added.

Reviewer #6 (Remarks to the Author):

I co-reviewed this manuscript with one of the reviewers who provided the listed reports. This is part of the Nature Communications initiative to facilitate training in peer review and to provide appropriate recognition for Early Career Researchers who co-review manuscripts.

Thank you for your assistance with improving the manuscript.

Point-to-points answers to the comments of the reviewers
(Comments from the reviewers in black. Our answers in red.)

Reviewer #1 (Remarks to the Author):

The authors adequately responded to all the technical comments, and the revised manuscript has significantly improved. While I am not fully convinced by the authors' arguments regarding the importance of their low-resolution structure, it is certainly a solid work. Whether it meets Nature Comms standards for impact and significance in view of the comments in my review of the original manuscript is an editorial decision.

We thank the reviewer for the careful evaluation of our revised manuscript and for recognizing that we have adequately addressed the technical comments and significantly improved the manuscript. We appreciate the reviewer's thoughtful assessment of the work.

Reviewer #2 (Remarks to the Author):

We thank the authors for their detailed responses to our comments. In our view, the revised manuscript has been substantially improved. The additional in vivo metal challenge assays and the intracellular Zn^{2+} quantification by ICP-MS significantly strengthen the functional interpretation and address my main concern regarding the distinction between metal-stimulated ATP hydrolysis and productive metal transport. The extended MD simulations, additional AlphaFold confidence analyses, and double-cysteine validation experiments also provide stronger support for the proposed N-terminal regulatory model.

Overall, we consider that the authors have satisfactorily addressed the major concerns raised in our previous review. The manuscript is now considerably stronger, both experimentally and conceptually.

We sincerely thank the reviewer for the careful evaluation of our revised manuscript and for the positive assessment of our revisions. We greatly appreciate the recognition that the additional in vivo metal challenge assays, intracellular Zn^{2+} quantification by ICP-MS, extended MD simulations, AlphaFold confidence analyses, and double-cysteine validation experiments have strengthened both the functional interpretation and the proposed regulatory model. We are grateful for the reviewer's constructive comments throughout the review process, which have significantly improved the quality of our manuscript.

We have only a few minor points that should be addressed before publication:

1- The authors should specify more clearly that direct intracellular metal quantification by ICP-MS was successfully performed for Zn^{2+}, whereas Cd^{2+} and Pb^{2+} transport are supported mainly by metal-resistance phenotypes. Thus, statements implying direct intracellular quantification for all three metals should be moderated.

The sentence regarding this has been amended to: These cells also show MmCadA-dependent resistance to intracellular metal Zn2+ accumulation (Supplementary Fig. 5m).

2- The authors should avoid referring to the ATPase activity measurements as biological replicates if they were obtained from three independent measurements using material from a single protein purification. This should be described as independent technical measurements from one preparation, unless independent purifications were performed.

This has been amended for all the relevant figures.

3- The term "zinc-specific" in the Abstract may be too restrictive given the demonstrated $Zn^{2+}/Cd^{2+}/Pb^{2+}$ activity and detoxification phenotype. A more precise wording would better reflect the promiscuous metal response of MmCadA while maintaining Zn^{2+} as the likely physiological substrate.

The sentence has been revised to: Here, we present the inward-open structure of a dimeric zinc-transporting specific PIB-type ATPase, resolved at 3.6 Å resolution.

4- The translational implications remain somewhat strong relative to the experimental scope. we recommend slightly softening the language in the Abstract and Conclusions to indicate that

the findings may inform future biotechnological or translational applications, rather than directly supporting them.

The sentence in the abstract has been revised to: Collectively, our findings shed critical new light on the regulation and transport mechanism of PIB-ATPases, with implications for both fundamental biology and the potential to inform future biotechnological and translational applications.

These are minor textual and interpretative issues. we do not consider that additional experiments are required. Subject to these clarifications, we would support publication of the manuscript.

We thank the reviewer for the positive assessment of our revised manuscript and for the constructive feedback throughout the review process. We appreciate the reviewer's recognition that no additional experiments were required and that the remaining issues were minor. We have carefully addressed all textual and interpretative comments in the revised manuscript and hope that the changes satisfactorily resolve the reviewer's remaining concerns.

Reviewer #3 (Remarks to the Author):

I co-reviewed this manuscript with one of the reviewers who provided the listed reports. This is part of the Nature Communications initiative to facilitate training in peer review and to provide appropriate recognition for Early Career Researchers who co-review manuscripts.

We thank the reviewer and co-reviewer for their time and careful evaluation of our manuscript.

Reviewer #4 (Remarks to the Author):

I thank the authors for their work on improving the manuscript based on comments from all reviewers (and for further satisfying my curiosity about how to make cells tolerate toxic levels of metals). I appreciate the much improved modelling section and the extended MD simulations, which has improved the former somewhat hand-wavy interpretations.

The authors have answered all my requests satisfactorily and I only have minor comments to the current manuscript version, as follows.

We sincerely thank the reviewer for the thoughtful and encouraging comments and for the careful evaluation of our revised manuscript. We greatly appreciate the recognition that the additional modelling, extended MD simulations, and other revisions have strengthened the manuscript and addressed the previous concerns. We are also pleased that our additional experiments helped satisfy the reviewer's curiosity regarding cellular tolerance to toxic metal levels. We thank the reviewer for the constructive feedback throughout the review process and have carefully addressed the remaining minor comments in the revised manuscript.

Fig 4 d: I am not sure whether a label for MBD-1 has been deliberately omitted? Also, I wonder whether rotating the protein a bit towards the right would make it easier to see that MBD-2 is indeed a bit more peripheral relative to MBD-1? From the current orientation, MBD-2 seems to almost partly overlap with MBD-1 (when zooming in, one of the beta strands of MBD-2 seems to cross the second from the top beta strand of MBD-1) – I appreciate that this is an artifact of the orientation, but a slightly rotated orientation might help the reader.

This has been amended.

In the track-changes version of the manuscript, page 13, line 4: “Similar smearing was not observed in the double mutant H99C/S643C (His-2/P-5 domain), but was seen in the double mutant (Fig. 4e)”. This sentence seems confusing.

The sentence has been changed to: In contrast, no similar smearing was observed for the H99C/S643C mutant (His-2/P-domains), but was seen in the quadruple mutant (A91C/H99C/A489C/S643C) (Fig. 4e).

Suppl fig 14, legend for b) MmCad*A* not *a*. For panel G, I would adjust the range of the y-axis to match panels C and E.

This has been corrected.

Minor inconsistency: Reporting Summary; Software and code states that MD simulations used GROMACS 2021 whereas Materials and Methods states GROMACS 2023.3.

The correct software version is GROMACS 2023.3.

To comply with the section 4a of the molecular dynamics reproducibility checklist, the authors should include either the full box dimensions (preferably) or the number of water molecules used for solvation, along with the ion concentration. Currently the only information given is the number of lipid molecules along with the number of counterions wherefore the box dimensions and the ion concentration cannot be extracted, reducing the reproducibility.

This information is provided now.

There are inconsistencies between section 4d of the MD checklist: “The initial structure was obtained from the AlphaFold model available through UniProt (ID: I4IY19). However, the prepared simulation systems, simulation input files, and final coordinate files are not currently provided as supplementary material or in a public repository.” And the Data availability section of the manuscript: “All files required for producing the trajectories of the MD simulations can be found at repository: https://zenodo.org/XXXX.”

We have now uploaded the MD reproducibility files (initial coordinate files, topology and parameter files, MD input files, and final coordinate files) to Zenodo.

Reviewer #5 (Remarks to the Author):

Even if Pb was present in the crystallization solution, it is meaningless unless its density is clearly visible in the structure. Especially for this protein, which is known for its heavy-metal transport properties, the authors' description of the structure as 'the first metal-induced' could still mislead readers into believing that it captures the bound transport ion. And in fact, this is not the first structure of heavy-metal containing P1B-ATPase. Cu-bound P1B-ATPase structure has already been published previously from the authors' group (PDB 7R0H, 2022, Nat Commun)

Therefore, it must be emphasized that this structure represents nothing more than a standard E1·ATP state. There is no structural evidence to suggest that this structure resulted from the functional action of heavy metals. The authors should clarify this point to avoid overinterpretation.

We agree with the reviewer and have revised the text accordingly. However, the structure remains the first inward-open structure of a zinc-transporting P1B-ATPase.

Reviewer #6 (Remarks to the Author):

I co-reviewed this manuscript with one of the reviewers who provided the listed reports. This is part of the Nature Communications initiative to facilitate training in peer review and to provide appropriate recognition for Early Career Researchers who co-review manuscripts.

We thank the reviewer and co-reviewer for their time and careful evaluation of our manuscript.
